# UFLC-Q-TOF-MS/MS-Based Screening and Identification of Flavonoids and Derived Metabolites in Human Urine after Oral Administration of Exocarpium Citri Grandis Extract

**DOI:** 10.3390/molecules23040895

**Published:** 2018-04-12

**Authors:** Xuan Zeng, Weiwei Su, Yuying Zheng, Hong Liu, Panlin Li, Weijian Zhang, Yuting Liang, Yang Bai, Wei Peng, Hongliang Yao

**Affiliations:** Guangdong Engineering & Technology Research Center for Quality and Efficacy Reevaluation of Post-Market Traditional Chinese Medicine, Guangdong Key Laboratory of Plant Resources, School of Life Sciences, Sun Yat-sen University, No. 135, Xingang Xi Road, Guangzhou 510275, China; zengx6@mail2.sysu.edu.cn (X.Z.); lsssww@126.com (W.S.); vicky_0224@126.com (Y.Z.); beauty19880711@163.com (H.L.); lipanlin@gmail.com (P.L.); zhweij6@mail2.sysu.edu.cn (W.Z.); lyting1906@126.com (Y.L.); white1504@163.com (Y.B.); pweiyu929@126.com (W.P.)

**Keywords:** flavonoids, metabolites, human urine, UFLC-Q-TOF-MS/MS, Exocarpium Citri Grandis

## Abstract

Exocarpium Citri grandis (ECG) is an important Traditional Chinese Medicine (TCM) for the treatment of cough and phlegm, and the flavonoids contained were considered the main effective components. To date, the systematic chemical profiling of these flavonoids and derived in vivo metabolites in human have not been well investigated. ECG was extracted using boiling water and then provided to volunteers for oral administration. Following the ingestion, urine samples were collected from volunteers over 48 h. The extract and urine samples were analyzed using ultra-fast liquid chromatography/quadrupole-time-of-flight tandem mass spectrometry (UFLC-Q-TOF-MS/MS) system to screen and identify flavonoids and derived in vivo metabolites. A total of 18 flavonoids were identified in the ECG extract, and 20 metabolites, mainly glucuronide and sulfate conjugates, were screened in urine samples collected post consumption. The overall excretion of naringenin metabolites corresponded to 5.45% of intake and occurred mainly within 4–12 h after the ingestion. Meanwhile, another 29 phenolic catabolites were detected in urine. Obtained data revealed that flavonoids were abundant in the ECG extract, and these components underwent extensive phase II metabolism in humans. These results provided valuable information for further study of the pharmacology and mechanism of action of ECG.

## 1. Introduction

Flavonoids, which are widely distributed in plants, have been proven to exert a wide range of biological and pharmacological activities [1,2,3]. The rapid screening and identification of flavonoids and derived in vivo metabolites have attracted significant attention and played a vital role in the pharmaceutical discovery process [4,5,6].

Exocarpium Citri grandis (ECG) (Huajuhong in Chinese), the epicarp of *C. grandis* ‘Tomentosa’, was officially listed in the first section of the 2015 edition of “Pharmacopoeia of the People’s Republic of China” and used as a primary ingredient in many famous Traditional Chinese Medicine (TCM) prescriptions [7,8]. ECG has been used as an effective antitussive and expectorant for many years [9,10]. Modern studies have demonstrated that ECG exhibited its antitussive effect via peripheral nerves [11,12,13]. In addition, contained flavonoids have been proven to be the main effective components in ECG [14,15,16]. Until now, the determination of flavonoids in ECG was mainly performed in HPLC, and only a few compounds were detected [17,18,19]. Existing results on in vivo metabolism of flavonoids in ECG are restricted in rats [20,21]. Furthermore, investigation on the systematic chemical profiling of these flavonoids and derived in vivo metabolites in humans is non-existent. Therefore, it is essential to determine the number of flavonoids in ECG and how these components are metabolized and then excreted.

The main objective of this work was to identify flavonoids in the ECG extract and derived metabolites in human urine after oral administration. With the ultra-fast liquid chromatography/quadrupole-time-of-flight tandem mass spectrometry (UFLC-Q-TOF-MS/MS) system, flavonoids in the ECG extract were systematically identified and quantified. After the consumption of 250 mL of the extract above, urine samples were collected from volunteers and then analyzed to identify metabolites. Furthermore, urinary metabolites were quantified to clarify the excretive profiles of flavonoids. The results obtained in this study would be valuable for further study of the pharmacology and mechanism of action of ECG.

## 2. Results and Discussion

### 2.1. Identification and Quantification of Flavonoids in ECG Extract

With the high resolution UFLC-Q-TOF-MS/MS system, a total of 18 flavonoids were identified in the ECG extract. These flavonoids were derived from eight aglycones, including naringenin, apigenin, diosmetin, eriodictyol, luteolin, hesperitin, isosakuranetin, and kaempferol. The detailed information, including compound description, formula, retention time (RT), and characteristic fragment ions were showed in Table 1 (Structures and product ion spectra were provided in Part I of Supplementary Materials). Among them, the main components (naringenin and hesperetin derivatives) were quantified. These derivatives were naringin (331.51 μmol/250 mL), naringin-4′*-O-*glucoside (2.89 μmol), naringenin (8.36 μmol), narirutin (1.40 μmol), narirutin-4′*-O-*glucoside (1.97 μmol), and hesperidin (0.08 μmol). In total, there are 346 μmol glycosides of naringenin and 0.08 μmol hesperidin in the 250 mL ECG extract.

Abundant naringin, which was considered the main effective component in ECG [14], was detected in the aqueous extract. These results reveal that a traditional boiling water extraction is a simple and feasible method to take advantage of ECG.

**Figure 1:**
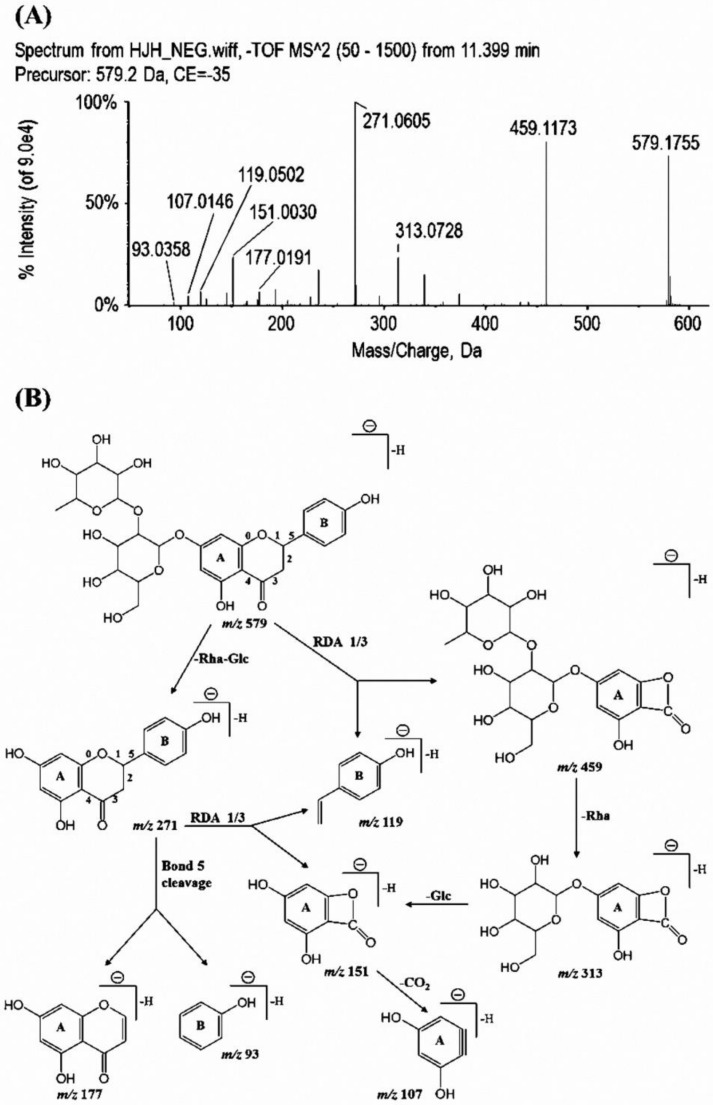
Product ion spectra (**A**) and proposed fragmentation pattern (**B**) of deprotonated naringin.

As shown in Table 1, three flavonoid aglycones (naringenin, kaempferol, and apigenin), and twelve flavonoid*-O-*glycosides (mainly flavonoid-7*-O-*glycosides, including naringin, hesperidin, neoeriocitrin, et al.) were identified in the ECG extract. Retro Diels-Alder (RDA) reactions were common in the MS fragmentation of flavonoids [22,23]. In the case of naringin, the signal at *m*/*z* 459 and 119 could be explained by cleavage of bonds 1 and 3 in the C ring. The fragment ion at *m*/*z* 313 and 151 were yielded by the neutral loss of a rhamnose moiety and subsequent neutral loss of a glucose moiety from the ion at *m*/*z* 459. The characteristic ion at *m*/*z* 271, which was proposed as deprotonated naringenin, was formed by the successive neutral loss of a rhamnose and a glucose moiety from naringin. An RDA reaction related the cleavage of bonds 1 and 3, and deprotonated naringenin also gave the fragment ion at *m*/*z* 151 and 119. Meanwhile, as a result of the cleavage of bond 5, the product ion at *m*/*z* 177 and 93 was generated from deprotonated naringenin. The product ion spectra and proposed fragmentations of deprotonated naringin were shown in Figure 1. Besides the twelve flavonoid*-O-*glycosides above, three flavonoid-*C*-glycosides (vicenin-2, lucenin-2,4′-methyl ether, and luteolin-6-*C*-glucoside) were also detected. A loss of 120 Da (C_4_H_8_O_4_), which was proposed as a fragment of glucose moiety, was detected in these three flavonoid-*C*-glycosides. However, the neutral loss of glycoside moiety was only identified in the fragmentation of luteolin-6-*C*-glucoside. Different from flavonoid*-O-*glycosides, RDA reactions were not observed in these flavonoid-*C*-glycosides. These results indicated that the glycosides bonding with carbon atoms exert a greater impact on structural characteristics of flavonoid-glycosides than that when bonding with oxygen atoms.

### 2.2. Identification and Quantification of Metabolites in Urine

**Figure 2:**
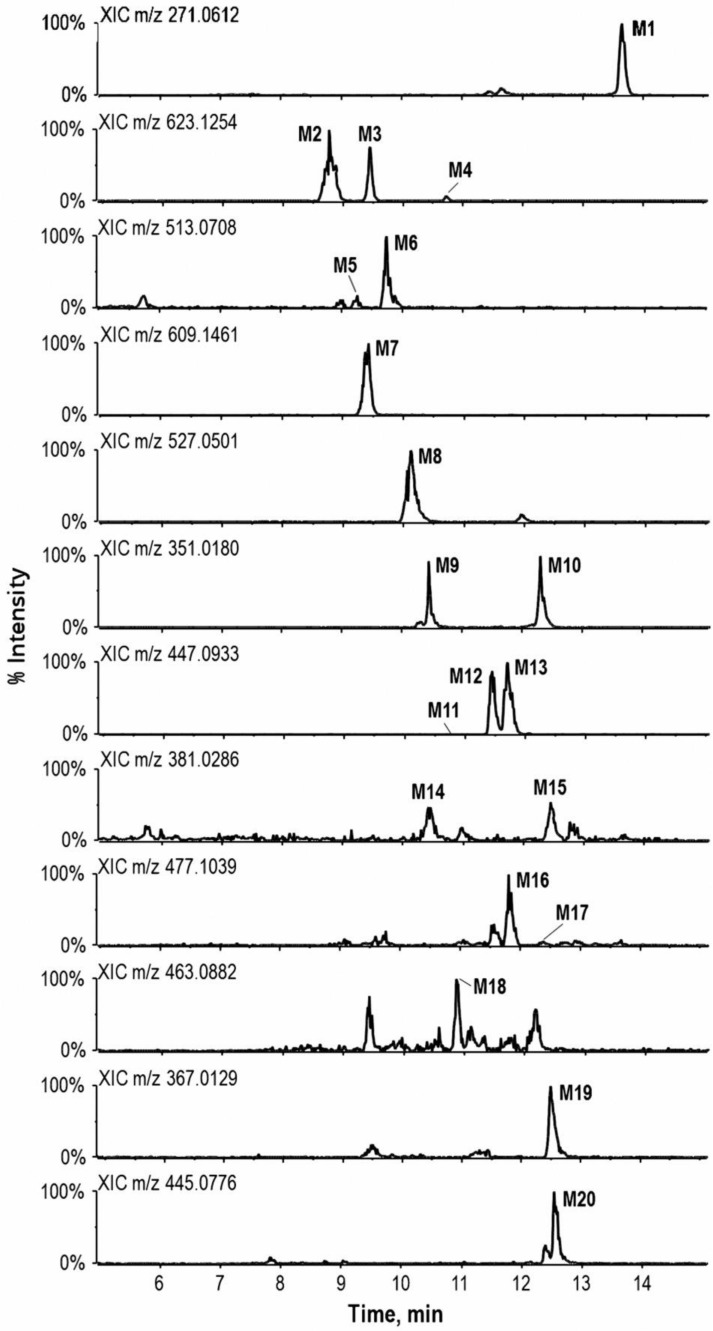
Extracted ion chromatograms of metabolites in urine samples. For peak identification, see Table 2.

After acute intake of 250 mL ECG extract, a total of 20 metabolites were detected and identified in urine samples. Table 2 showed the compound description, elemental composition, elution time, and characteristic fragment ions of these metabolites (structures and product ion spectra were provided in Part III of Supplementary Materials). In addition, typical extracted ion chromatograms of identified metabolites were illustrated in Figure 2.

Three metabolites (M11, M12, and M13), which yielded deprotonated molecular ion of *m*/*z* 447 and characteristic fragments of *m*/*z* 271, were detected in urine (shown in Table 2). With authentic standards, M12 and M13 were unambiguously identified as naringenin-7*-O-*glucuronide and naringenin-4′*-O-*glucuronide, respectively. Due to its low acidity, the 5-OH was generally considered to be the least reactive among the three possible conjunct sites for naringenin (7-, 4′-, and 5-OH) [24]. Furthermore, combined with an early study [25], M11 was tentatively characterized as naringenin-5*-O-*glucuronide. After the loss of a moiety weighted 176 Da (glucuronyl moiety), M2, M3, and M4 all gave characteristic fragments at *m*/*z* 271, 175, 151, which were similar to that of naringenin*-O-*glucuronides. With reported results [26,27], M2, M3, and M4 were identified as naringenin-4′,7*-O-*diglucuronide, naringenin-5,7*-O-*diglucuronide, and naringenin-4′,5*-O-*diglucuronide, correspondingly. M9 and M10, which gave the deprotonated ion at *m*/*z* 351 (80 Da (SO_3_) more than that of naringenin) and exhibited a similar fragmentation pattern as naringenin, were supposed as naringenin*-O-*sulfates. Furthermore, given the least reactivity of 5-OH for naringenin [24] and the relative retention times of naringenin sulfates, M9 and M10 were further identified as naringenin-4′*-O-*sulfate and naringenin-7*-O-*sulfate, respectively. In addition to the above metabolites, naringenin*-O-*glucoside*-O-*sulfates, naringenin*-O-*glucoside*-O-*glucuronide, and naringenin*-O-*glucuronide*-O-*sulfate were also detected in some urine samples. However, due to the unavailability of reference standards for these metabolites, other methodologies will be needed to elucidate their final structure.

As for hesperetin, eriodictyol, and apigenin, probably due to little ingestion, several metabolites were identified. Co-chromatography with an authentic standard established that M14 was hesperetin-3′*-O-*sulfate. According to a study carried out by Brand and co-researchers [28], hesperetin was mainly conjugated at positions 7- and 3′-OH, and hesperetin-3′*-O-*sulfate was eluted earlier than hesperetin-7*-O-*sulfate. Thus, M15 could be tentatively identified as hesperetin-7*-O-*sulfate. Compared with reference standards, M16 and M17 were definitely identified as hesperetin-7*-O-*glucuronide and hesperetin-3′*-O-*glucuronide, respectively. Similar to the fragmentation pattern of naringenin glucuronides, the hesperetin glucuronides produced two major characteristic ions via the loss of the glucuronyl moiety (giving an ion of *m*/*z* 301) or the aglycon hesperetin (giving an ion of *m*/*z* 175), respectively. Following the loss of respective ligand (glucuronide or sulfate), M18 and M19 both gave characteristic fragments at *m*/*z* 151 and 135, which were proposed as the products of Retro Diels-Alder (RDA) reactions. With reported results [29], M18 and M19 were identified as eriodictyol*-O-*glucuronide and eriodictyol*-O-*sulfate, respectively. However, it is difficult to assign the specified conjunct sites of ligands for these two metabolites due to the four binding sites of eriodictyol (5-, 7-, 3′-, and 4′-OH). Based on an early report [30], M20 was tentatively identified as apigenin glucuronide. Nevertheless, given three hydroxyls on the apigenin skeleton, M20 could be tentatively identified as the 5-, 7-, or 4′-glucuronide.

Mediated by lactase-phlorizin hydrolase and intestinal microbiota, the hydrolysis of flavonoid*-O-*glycosides was generally considered the first and determinant step in the absorption of flavonoids [31]. Consistent with reported results [32], free flavonoid*-O-*glycosides (naringin, narirutin, hesperidin, neoeriocitrin, et al.) were not detected in urine samples. Following the hydrolysis of flavonoid*-O-*glycosides, corresponding aglycones were generated and subsequently engaged in glucuronidation and sulfation, giving rise to a series of conjunct metabolites. As shown in Table 3, flavonoid aglycone glucuronides and sulfates (mainly naringenin glucuronides and sulfates) were the major metabolites in urine after the intake of a 250 mL ECG extract, aligned with reported studies [33,34,35]. Based on identified metabolites shown in Table 2, it was rational to speculate that flavonoids derived from ECG underwent extensive phase II metabolism in the human body. Different with the metabolism profile in rats [20,21], naringin glucuronides, naringin sulfates, and other naringin derivatives were not detected in human urine, showing species variations.

In a study conducted by Zhang and Brodbelt [24], naringenin glucuronides, naringenin sulfates, naringenin glucuronide sulfates, and a naringenin diglucuronide were screened as the metabolites of naringin and narirutin in urine after consumption of grapefruit juice. However, the specific conjunct sites of ligands for naringenin and the urinary excretion were undefined. As to the current work, with more authentic standards and updated findings, a total of 19 conjugative metabolites that were derived from naringenin, hesperetin, eriodictyol, and apigenin were identified in urine collected after the consumption of ECG extracts. Meanwhile, the urinary excretions of major metabolites were quantified along with the metabolite identification.

Naringenin and hesperetin metabolites, whose content was much higher than other metabolites, were quantified in urine of 0–48 h after the supplementation (shown in Table 3). There no metabolites detected in the urine collected prior to the ingestion. As shown in Table 3, naringenin-7*-O-*glucuronide, and naringenin-4′*-O-*glucuronide were the predominated metabolites of naringenin in urine after the intake of ECG extract, followed by naringenin*-O-*glucoside*-O-*sulfate (M6, RT = 9.9 min), free naringenin, naringenin*-O-*glucuronide*-O-*sulfate, and naringenin*-O-*glucoside*-O-*glucuronide. The overall excretion of naringenin metabolites corresponded to 5.45% of intake and occurred mainly within 4–12 h after the ingestion (Table 3). Several hesperetin metabolites, including hesperetin*-O-*glucuronides and hesperetin*-O-*sulfates, were excreted in little amounts, probably due to little ingestion of hesperidin. However, the overall recovery of hesperetin was higher than that of naringenin, which was equivalent to 58.7% of intake (Table 3).

Catalyzed by intestinal microflora, unabsorbed flavonoids were further metabolized into phenolic catabolites [36,37]. In this work, a total of 29 phenolic catabolites were screened and identified in urine samples collected after the oral administration of ECG extract. (Detailed information, structures, and product ion spectra were shown in part III of Supplementary Materials). However, most of these catabolites were also detected in urine samples collected before the oral administration. Generally, phenolic catabolites maintain a high concentration level in humans [38]. Abundant polyphenols in diets inevitably yield a number of catabolites in human intestines [39]. Meanwhile, several phenolic acids are generated in the metabolic processes of the human body [27]. For example, 3-(4′-hydroxy)-phenylpropionic acid is a major metabolite of tyrosine in humans [40]. Therefore, although a low-flavonoid diet was followed by the volunteers, phenolic catabolites were also detected in urine samples collected before the oral administration. Hence, without other methodologies, it is difficult to speculate about the exact amounts of phenolic catabolites derived from specific flavonoids in ECG extract.

## 3. Experimental

### 3.1. Chemicals and Reagents

The reference standards naringin, hesperidin, and apigenin were obtained from the National Institute for the Control of Pharmaceutical and Biological Products (Beijing, China). Naringenin, hesperetin, and MS grade formic acid were purchased from Sigma-Aldrich (St. Louis, MO, USA). Hesperetin-7*-O-*glucuronide, hesperetin-3′*-O-*glucuronide, and hesperetin-7*-O-*sulfate were acquired from Toronto Research Chemicals (Toronto, ON, Canada). Naringenin-7*-O-*glucuronide was purchased from Cayman Chemical Company (Ann Arbor, MI, USA). Naringenin-4′*-O-*glucuronide was obtained from Shanghai ZZBIO Co., Ltd. (Shanghai, China). Naringenin-7*-O-*glucoside was acquired from ChromaDex Inc. (Irvine, CA, USA). Rhoifolin was purchased from Shanghai Tauto Biotech Co., Ltd. (Shanghai, China). And kaempferol was obtained from Shanghai Yuanye Biological Co., Ltd. (Shanghai, China). The stable isotope labeled internal standard, naringin-d4, was supplied from Artis-Chem Co. Ltd. (Shanghai, China).

MS grade methanol was purchased from Fisher Scientific Inc. (Fair Lawn, NJ, USA). Acetonitrile of HPLC grade was obtained from Honeywell B&J Chemicals Inc. (Morristown, NJ, USA). Milli-Q grade water was purified by reverse-osmosis and filtered through a 0.22 μm membrane filter before use.

ECG was obtained from Huazhou Huajuhong Medicinal Materials Development Co., Ltd. (with GMP certificate). These samples were authenticated by Prof. Wenbo Liao from Sun Yat-sen University. In addition, the voucher specimens were deposited in our laboratory.

### 3.2. Preparation of ECG Extract

The ECG extract used in the feeding study was prepared with common processing methods in China. Briefly, ECG was cut into small pieces. In addition, 60-g fragmented samples were weighed and extracted with 1500 mL boiling water for 30 min. After filtering, the aqueous extract without residue was used in the study and a partial aqueous extract was stored at −80 °C for chemical profile analysis.

### 3.3. Study Design

Five healthy volunteers (three men and two women), aged 23–28 years and with BMI (Body Mass Index, in kg/m^2^) from 19.4 to 22.8 were recruited. These volunteers were nonsmokers, non-pregnant female, and not taking medication. All subjects were informed about the objectives, method, and risks of this study. All of them were asked to sign informed consent before their inclusion in the trial. Volunteers were required to follow a diet low in flavonoids, which excluded citrus fruit derived food, cruciferous vegetables, tomato, soybean, fresh ginger, chamomile, apple, grape, and beverages such as coffee, tea, coke, soda water, fruit juice, and wine, for 48 h before the ingestion.

On the day of supplementation, after an overnight fast, each volunteer drank 250 mL ECG extract. Volunteers were provided with a light breakfast (bread, eggs, and water) 2 h after the supplementation, and remained on a low-flavonoid diet for a further 48 h until the final urine samples were collected. Urine samples were collected once prior to drinking and over six time periods (0–4, 4–8, 8–12, 12–24, 24–36, 36–48 h) after consumption. Urine excreted in each time period was mixed, measured for its volume, and then stored at −80 °C before analysis.

### 3.4. Sample Preparation

All reference standards were accurately weighed, dissolved in methanol, stored at 4 °C and brought to room temperature before use. The stable isotope labeled internal standard naringin-d4 was dissolved in acetonitrile and prepared at 15 μg/mL used for protein precipitation. A sample preparation processes for ECG extract and urine were the same. An aliquot of 100 μL of the liquid sample above was transferred into a 1.5 mL polypropylene tube. After adding 200 μL volume acetonitrile for dissolving naringin-d4, the sample was vortex-mixed for 3 min and centrifuged at 15,000× *g* for 30 min at 25 °C. Finally, an aliquot of 10 μL supernatant was injected into the UFLC-Q-TOF-MS/MS for analysis.

### 3.5. UFLC-Q-TOF-MS/MS Analysis

Sample analysis was carried out on a Shimadzu UFLC XR instrument (Shimadzu Corp., Kyoto, Japan) equipped with an on-line degasser, a binary pump, and an autosampler. Chromatographic separation was performed on a Phenomenex Kinetex C_18_ column (3.0 × 150 mm, 2.6 μm, 100 Å; Phenomenex, Torrance, CA, USA) at 40 °C and eluted at a flow rate of 0.3 mL/min. The mobile phase was composed of 0.1% aqueous formic acid (*v*/*v*) (A) and methanol with 0.1% formic acid (*v*/*v*) (B). The following gradient elution program was used: linear gradient from 5% to 65% B (0–10 min), 65–100% B (10–30 min), and isocratic 100% B for 10 min. A 5-min post-run time was set to equilibrate the column.

Without splitting, the UFLC effluent was introduced directly to a hybrid triple quadrupole time-of-flight mass spectrometer (Triple TOF^TM^ 5600 plus; AB Sciex, Foster City, CA, USA) equipped with an electrospray ionization source. The main instrumental conditions were as follows: ion source gas 1 and gas 2 were both 55 psi, curtain gas was 35 psi, ion source temperature was 550 °C, ion spray voltage floating was 5500 V in positive mode while 4500 V in negative mode, collision energy was 35 V, collision energy spread was 25 V, and declustering potential was 80 V. Nitrogen was used as nebulizer and auxiliary gas. Aqueous extract of ECG was analyzed in both positive and negative ionization modes, and the TOF-MS scan range was from *m*/*z* 100 to 1500. While urine samples were determined only in negative ionization mode for stronger signal response. Data acquisition was carried out using Analyst^®^ TF 1.6 software (AB Sciex, Foster City, CA, USA) in IDA (information-dependent acquisition) mode. Identifications were based on chromatographic elution time, chemical composition, MS fragmentation pattern, and comparisons with available standards and references, as well as the mass spectral library (LibraryView, Version 1.0; AB Sciex, Foster City, CA, USA).

Naringenin and hesperetin derivatives, including partial compounds in ECG extract, as well as metabolites in urine, were quantified with chromatographic peak areas acquired in TOF-MS full scan and expressed relative to available authentic standards. Meanwhile, subjected to the unavailability of authentic standards, partial derivatives were prudently quantified as corresponding structural analogues, aligned with another study [32]. Naringin-4′*-O-*glucoside, narirutin, and narirutin-4′*-O-*glucoside were quantified as naringin. Naringenin-5*-O-*glucuronide, naringenin*-O-*diglucuronide, and naringenin*-O-*glucuronide*-O-*sulfate were quantified as naringenin-7*-O-*glucuronide. Naringenin*-O-*glucoside*-O-*glucuronide, and naringenin*-O-*glucoside*-O-*sulfate were quantified as naringenin-7*-O-*glucoside. Hesperetin-5*-O-*glucuronide was quantified as hesperetin-7*-O-*glucuronide. Naringenin-4′*-O-*sulfate, naringenin-7*-O-*sulfate, and hesperetin-3′*-O-*sulfate were quantified as hesperetin-7*-O-*sulfate.

Calibration curves that were used in the quantification of constituents in ECG extract were prepared from reference compounds dissolved in 50% methanol (*v*/*v*), while that used in the quantification of urinary metabolites was prepared from stock solutions by diluting with urine collected before the administration. All of these calibration curves covered the concentration range from 5 to 500 ng/mL. Calibration curves were constructed and fitted by linear regression analysis (*R*^2^ > 0.99) to plot the peak area ratio of analyte relative to the internal standard against the analyte concentrations. The intra-run precision, which ranges from 1.4% to 10.7%, was considered acceptable. Partial samples, whose concentrations exceeded the range of calibration curve, were diluted according to the actual situation.

## 4. Conclusions

In summary, using the UFLC-Q-TOF-MS/MS system, a total of 18 flavonoids was detected in the ECG extract, and naringenin derivatives as well as hesperidin contained within were quantified. Five volunteers were recruited and required to drink 250 mL ECG extract. Urine samples were collected after the supplementation and then analyzed with UFLC-Q-TOF-MS/MS. Finally, a total of 20 metabolites were identified and partly quantified in urine. Based on detected metabolites, flavonoids derived from ECG were considered to undergo extensive in vivo phase II metabolism (mainly hydrolysis, glucuronidation, and sulfation). Although 29 phenolic catabolites were detected in urine, it is hard to determine the exact amounts of phenolic catabolites derived from specific flavonoids due to multiple interferences. These results could be helpful for further study of the pharmacology and mechanism of action of ECG.

## Figures and Tables

**Table 1:** Identification of flavonoids in the ECG extract by UFLC-Q-TOF-MS/MS.

No.	Identified Compounds	Formula	RT (min)	[M + H]^+^ (Error, ppm)	[M − H]^−^ (Error, ppm)	Fragment Ions in Positive (+) Ion Mode ^a^	Fragment Ions in Negative (−) Ion Mode
	**Apigenin derivatives**						
F1	Vicenin-2	C_27_H_30_O_15_	9.5	595.1604 (−1.7)	593.1552 (2.4)	577.1506[M + H-H_2_O]^+^, 559.1432[M + H-2H_2_O]^+^, 379.1026, 337.0692, 325.0690, 295.0585	575.1548[M − H-H_2_O]^−^, 503.1236, 473.1113[M − H-C_4_H_8_O_4_]^−^, 383.1025, 353.0673[M − H-2C_4_H_8_O_4_]^−^, 325.0730, 297.0821
F2	Rhoifolin ^b,c^	C_27_H_30_O_14_	12.1	579.1640 (2.3)	577.1625 (4.1)	433.1226[M + H-Rha]^+^, 271.0569[M + H-Rha-Glc]^+^	413.1364[M − H-Rha-H_2_O]^−^, 269.0465[M − H-Rha-Glc]^−^
F3	Apigenin ^b^	C_15_H_10_O_5_	14.9	271.0575 (1.9)	269.0476 (5.1)	153.0182[M + H-C_8_H_6_O]^+^, 119.0203[M + H-C_7_H_4_O_4_]^+^, 91.0126	227.0379, 151.0231[M − H-C_8_H_6_O]^−^, 11.03567[M − H-C_7_H_4_O_4_]^−^, 107.0411[M − H-C_8_H_6_O-CO_2_]^−^
	**Naringenin derivatives**						
F4	Narirutin ^c^	C_27_H_32_O_14_	9.7	581.1848 (3.3)	579.1782 (2.6)	527.1632,419.1288[M + H-ORha]^+^, 383.1061, 339.1104, 315.0832[M + H-Rha-C_8_H_8_O]^+^, 273.0724[M + H-Rha-Glc]^+^, 195.0269, 153.0924[M + H-Rha-Glc-C_8_H_8_O]^+^, 85.0293	459.1205[M − H-C_8_H_8_O]^−^, 313.1028[M − H-C_8_H_8_O-Rha]^−^, 271.0622[M − H-Rha-Glc]^−^, 151.0035[M − H-Rha-Glc-C_8_H_8_O]^−^, 119.0856[M − H-Rha-Glc-C_7_H_4_O_4_]^−^
F5	Narirutin-4′*-O-*glucoside	C_33_H_42_O_19_	9.7	743.2304 (−2.1)	741.2358 (4.9)	581.1742[M + H-Glc]^+^, 435.1284[M + H-Glc-Rha]^+^, 315.0793[M + H-Glc-Rha-C_8_H_8_O]^+^, 273.0721[M + H-Rha-2Glc]^+^, 195.0278, 85.0453	579.1770[M − H-Glc]^−^, 459.1195[M − H-Glc-C_8_H_8_O]^−^, 433.1068[M − H-Glc-Rha]^−^, 271.0618[M − H-Rha-2Glc]^−^, 151.0425[M − H-Rha-2Glc-C_8_H_8_O]^−^
F6	Naringin-4′*-O-*glucoside	C_33_H_42_O_19_	10.4	743.2147 (-1.8)	741.2335 (4.3)	581.1750[M + H-Glc]^+^, 459.1250, 435.1136[M + H-Glc-Rha]^+^, 417.1221[M + H-Glc-Rha-H_2_O]^+^, 315.0954[M + H-Glc-Rha-C_8_H_8_O]^+^, 297.0732[M + H-Glc-Rha-C_8_H_8_O-H_2_O]^+^, 273.0726[M + H-Rha-2Glc]^+^, 219.0273, 153.0451[M + H-Rha-2Glc-C_8_H_8_O]^+^, 129.0577	621.1738[M − H-C_4_H_8_O_4_]^−^, 459.1203[M − H-Glc-C_8_H_8_O]^−^, 271.0624[M − H-Rha-2Glc]^−^, 177.0857[M − H-Rha-2Glc-C_6_H_6_O]^−^, 151.0031[M − H-Rha-2Glc-C_8_H_8_O]^−^
F7	Naringin ^b, c^	C_27_H_32_O_14_	11.4	581.1808 (1.3)	579.1755 (−1.9)	435.1426[M + H-Rha]^+^, 419.1300[M + H-ORha]^+^, 383.1098[M + H-Orha-2H_2_O]^+^, 339.0828, 315.0869[M + H-Rha-C_4_H_8_O_4_]^+^, 273.0725[M + H-Rha-Glc]^+^, 195.0269, 153.0167[M + H-Rha-Glc-C_8_H_8_O]^+^, 129.0630, 85.0581	459.1173[M − H-C_8_H_8_O]^−^, 373.0950, 339.0924, 313.0728[M − H-C_8_H_8_O-Rha]^−^, 271.0605[M − H-Rha-Glc]^−^, 235.0855, 193.0628, 177.0069[M − H-Rha-Glc-C_6_H_6_O]^−^, 151.0030[M − H-Rha-Glc-C_8_H_8_O]^−^, 119.0502[M − H-Rha-Glc-C_7_H_4_O_4_]^−^, 107.0421[M − H-Rha-Glc-C_8_H_8_O-CO_2_]^−^, 93.0087[M − H-Rha-Glc-C_9_H_7_O_4_]^−^
F8	Melitidin	C_33_H_40_O_18_	12.4	725.2217 (2.5)	723.2220 (3.3)	671.0857[M + H-3H_2_O]^+^, 603.1236, 579.1659[M + H-Rha]^+^, 561.1258[M + H-Rha-H_2_O]^+^, 509.1124[M + H-3H_2_O-ORha]^+^, 461.1407, 417.0954[M + H-Rha-C_6_H_10_O_5_]^+^, 381.0322[M + H-Rha-Glc-2H_2_O-C_6_H_10_O_5_]^+^, 339.1008, 315.0861, 273.0741[M + H-Rha-Glc-C_6_H_8_O_4_]^+^, 195.0862, 153.1204[M + H-Rha-Glc-C_6_H_8_O_4_-C_8_H_8_O]^+^, 127.0382, 85.0562	661.1856[M − H-CO_2_-H_2_O]^−^, 621.1882, 579.1767[M − H-C_6_H_8_O_4_]^−^, 541.1564[M − H-Rha-2H_2_O]^−^, 501.1284, 459.1204[M − H-C_6_H_8_O_4_-C_8_H_8_O]^−^, 339.0739, 271.0615[M − H-Rha-Glc-C_6_H_8_O_4_]^−^, 151.0034[M − H-Rha-Glc-C_6_H_8_O_4_-C_8_H_8_O]^−^
F9	Naringenin ^b,c^	C_15_H_12_O_5_	13.7	273.0734 (−1.7)	271.0606 (−2.8)	153.0171[M + H-C_8_H_8_O]^+^, 147.0433, 123.0510, 119.0486, 91.0548	177.0187[M − H-C_6_H_6_O]^−^, 151.0029[M − H-C_8_H_8_O]^−^, 119.0501[M − H-C_7_H_4_O_4_]^−^, 107.0147[M − H-C_8_H_8_O-CO_2_]^−^, 93.0360[M − H-C_9_H_6_O_4_]^−^, 83.0522
	**Diosmetin derivatives**						
F10	Lucenin-2,4′-methyl ether	C_28_H_32_O_16_	9.8	625.1724 (2.9)	623.1686 (2.6)	607.1573[M + H-H_2_O]^+^, 487.1167[M + H-H_2_O-C_4_H_8_O_4_]^+^, 439.1033, 409.0986, 355.0809, 317.0643	533.1122[M − H-C_3_H_6_O_3_]^−^, 503.1242[M − H-C_4_H_8_O_4_]^−^, 413.0992[M − H-C_4_H_8_O_4_-C_3_H_6_O_3_]^−^, 383[.0800M − H-2C_4_H_8_O_4_]^−^, 312.0662
F11	Neodiosmin	C_28_H_32_O_15_	12.2	609.1778 (−1.3)	607.1754 (0.3)	301.0677[M + H-Rha-Glc]^+^, 286.0449[M + H-Rha-Glc-CH_3_]^+^	299.0580[M − H-Rha-Glc]^−^, 284.0338[M − H-Rha-Glc-CH_3_]^−^
	**Eriodictyol derivatives**						
F12	Eriocitrin	C_27_H_32_O_15_	10.2	597.1753 (2.6)	595.1723 (2.9)	579.1625[M + H-H_2_O]^+^, 451.1230[M + H-Rha]^+^, 435.1149, 289.0689[M + H-Rha-Glc]^+^, 235.0622, 169.0093, 147.0524, 85.0323	475.1138, 431.1033[M − H-Rha-H_2_O]^−^, 287.0576[M − H-Rha-Glc]^−^, 166.9979
F13	Neoeriocitrin	C_27_H_32_O_15_	10.7	597.1748 (3.5)	595.1709 (2.3)	451.1224[M + H-Rha]^+^, 435.1285[M + H-ORha]^+^, 399.1005[M + H-Orha-2H_2_O]^+^, 331.0988, 315.0838[M + H-Orha-C_4_H_8_O_4_]^+^, 289.0685[M + H-Rha-Glc]^+^, 273.1004[M + H-Rha-OGlc]^+^, 219.0854, 195.0270, 153.0162[M + H-Rha-OGlc-C_8_H_8_O_2_]^+^, 129.0065, 85.0425	475.1038[M − H-C_4_H_8_O_4_]^−^, 459.1162[M − H-C_8_H_8_O_2_]^−^, 339.0729[M − H-C_4_H_8_O_4_-C_8_H_8_O_2_]^−^, 287.0564[M − H-Rha-Glc]^−^, 235.0822, 193.0321, 151.0034[M − H-Rha-Glc-C_8_H_8_O_2_]^−^, 135.0244[M − H-Rha-Glc-C_7_H_4_O_4_]^−^, 107.0626[M − H-Rha-Glc-C_8_H_8_O_2_-CO_2_]^−^
	**Luteolin derivatives**						
F14	Luteolin-6-*C*-glucoside	C_21_H_20_O_11_	10.5	449.1021 (−4.3)	447.0958 (0.9)	431.1036[M + H-H_2_O]^+^, 413.0884[M + H-2H_2_O]^+^, 353.0615, 329.0612[M + H-C_4_H_8_O_4_]^+^, 299.0529, 287.0614[M + H-Glc]^+^, 243.0261	429.0800[M − H-H_2_O]^−^, 411.0784[M − H-2H_2_O]^−^, 369.0551, 357.0621, 327.0528[M − H-C_4_H_8_O_4_]^−^, 297.0419, 285.0403[M − H-Glc]^−^, 229.0552, 133.0311
F15	Veronicastroside	C_27_H_30_O_15_	11.9	595.1625 (4.2)	593.1586 (1.8)	449.1057[M + H-Rha]^+^, 287.0533[M + H-Rha-Glc]^+^	285.0422[M − H-Rha-Glc]^−^
	**Hesperitin derivatives**						
F16	Hesperidin ^b,c^	C_28_H_34_O_15_	11.6	ND ^d^	609.1852 (4.9)	ND	459.1656[M − H-C_9_H_10_O_2_]^−^, 301.0713[M − H-Rha-Glc]^−^, 235.0662, 151.0016[M − H-Rha-Glc-C_9_H_10_O_2_]^−^
	**Isosakuranetin derivatives**						
F17	Poncirin	C_28_H_34_O_14_	13.9	ND	593.1955 (−3.9)	ND	549.1722[M − H-CO-CH_4_]^−^, 491.1596, 449.1469, 285.0889[M − H-Rha-Glc]^−^, 227.0724, 143.0356, 125.0249, 99.0468
	**Kaempferol derivatives**						
F18	Kaempferol ^b^	C_15_H_10_O_6_	14.7	ND	285.0411 (3.3)	ND	267.0355[M − H-H_2_O]^−^, 256.0218, 228.0296

^a^ The losses are: Rha = rhamnose moiety, Glc = glucose moiety. ^b^ Confirmation in comparison with authentic standards. ^c^ Confirmation in comparison with mass spectral library (Natural Products HR-MS/MS Spectral Library, Version 1.0, AB Sciex). ^d^ ND: not detected.

**Table 2:** Identification of flavonoid metabolites in human urine after the consumption of 250 mL ECG extract by UFLC-Q-TOF-MS/MS.

No.	Identified Metabolites	Formula	RT (min)	[M − H]^−^ (Error, ppm)	Fragment Ions in Negative (−) Ion Mode ^a^
	**Naringenin metabolites**				
M1	Naringenin ^b^	C_15_H_12_O_5_	13.6	271.0623 (3.5)	177.0184[M − H-C_6_H_6_O]^−^, 151.0031[M − H-C_8_H_8_O]^−^, 119.0505[M − H-C_7_H_4_O_4_]^−^, 107.0145[M − H-C_8_H_8_O-CO_2_]^−^, 93.0356[M − H-C_9_H_6_O_4_]^−^
M2	Naringenin-4′,7*-O-*diglucuronide	C_27_H_28_O_17_	8.8	623.1321 (3.7)	447.0966[M − H-GlcUA]^−^, 313.0733[M − H-GlcUA-C_4_H_6_O_5_]^−^, 271.0621[M − H-2GlcUA]^−^, 175.0301[M − H-NE-GlcUA]^−^, 151.0046[M − H-2GlcUA-C_8_H_8_O]^−^, 113.0233[M − H-NE-GlcUA-CO_2_-H_2_O]^−^
M3	Naringenin-5,7*-O-*diglucuronide	C_27_H_28_O_17_	9.4	623.1348 (-1.2)	447.0989[M − H-GlcUA]^−^, 271.0641[M − H-2GlcUA]^−^, 175.0259[M − H-NE-GlcUA]^−^, 151.0054[M − H-2GlcUA-C_8_H_8_O]^−^, 113.0258[M − H-NE-GlcUA-CO_2_-H_2_O]^−^
M4	Naringenin-4′,5*-O-*diglucuronide	C_27_H_28_O_17_	10.7	623.1338 (2.9)	447.0966[M − H-GlcUA]^−^, 313.0709[M − H-GlcUA-C_4_H_6_O_5_]^−^, 271.0619[M − H-2GlcUA]^−^, 175.0320[M − H-NE-GlcUA]^−^, 151.0028[M − H-2GlcUA-C_8_H_8_O]^−^, 113.0198[M − H-NE-GlcUA-CO_2_-H_2_O]^−^
M5	Naringenin*-O-*glucoside*-O-*sulfate	C_21_H_22_O_13_S	9.3	513.0744 (0.6)	433.1198[M − H-SO_3_]^−^, 313.0704[M − H-SO_3_-C_4_H_8_O_4_]^−^, 271.0635[M − H-SO_3_-Glc]^−^, 151.0025[M − H-SO_3_-Glc-C_8_H_8_O]^−^
M6	Naringenin*-O-*glucoside*-O-*sulfate	C_21_H_22_O_13_S	9.9	513.0761 (1.5)	433.1186[M − H-SO_3_]^−^, 351.0199[M − H-Glc]^−^, 313.0727[M − H-SO_3_-C_4_H_8_O4]^−^, 271.0624[M − H-SO_3_-Glc]^−^, 151.0031[M − H-SO_3_-Glc-C_8_H_8_O]^−^, 119.0108[M − H-SO_3_-Glc-C_7_H_4_O_4_]^−^
M7	Naringenin*-O-*glucoside*-O-*glucuronide	C_27_H_30_O_16_	9.4	609.1517 (2.9)	489.1078[M − H-C_4_H_8_O_4_]^−^, 447.0964[M − H-Glc]^−^, 429.0871[M − H-Glc-H_2_O]^−^, 313.0716[M − H-Glc-C_4_H_6_O_5_]^−^, 271.0622[M − H-Glc-GlcUA]^−^, 175.0248[M − H-NE-Glc]^−^, 151.0040[M − H-Glc-GlcUA-C_8_H_8_O]^−^, 113.0249[M − H-NE-Glc-CO_2_-H_2_O]^−^, 99.0554, 85.0336[M − H-NE-Glc-CO_2_-H_2_O-CO]^−^
M8	Naringenin*-O-*glucuronide*-O-*sulfate	C_21_H_20_O_14_S	10.1	527.0545 (0.7)	447.0957[M − H-SO_3_]^−^, 351.0192[M − H-GlcUA]^−^, 271.0611[M − H-SO3-GlcUA]^−^, 175.0222[M − H-NE-SO_3_]^−^, 151.0030[M − H-SO3-GlcUA-C_8_H_8_O]^−^, 113.0250[M − H-NE-SO_3_-CO_2_-H_2_O]^−^
M9	Naringenin-4′*-O-*sulfate	C_15_H_12_O_8_S	10.3	351.0192 (-0.8)	271.0615[M − H-SO_3_]^−^, 177.0203[M − H-SO_3_-C_6_H_6_O]^−^, 151.0034[M − H-SO_3_-C_8_H_8_O]^−^, 119.0322[M − H-SO_3_-C_7_H_4_O_4_]^−^, 107.0156[M − H-SO_3_-C_8_H_8_O-CO_2_]^−^, 93.0244[M − H-SO_3_-C_9_H_6_O_4_]^−^
M10	Naringenin-7*-O-*sulfate	C_15_H_12_O_8_S	12.3	351.0198 (1.3)	271.0620[M − H-SO_3_]^−^, 177.0198[M − H-SO_3_-C_6_H_6_O]^−^, 151.0043[M − H-SO_3_-C_8_H8O]^−^, 119.0509[M − H-SO_3_-C_7_H_4_O_4_]^−^, 107.0144[M − H-SO_3_-C_8_H_8_O-CO_2_]^−^, 93.0212[M − H-SO_3_-C_9_H_6_O_4_]^−^
M11	Naringenin-5*-O-*glucuronide	C_21_H_20_O_11_	10.7	447.0942 (2.5)	326.2070,271.0619[M − H-GlcUA]^−^, 151.0038[M − H-GlcUA-C_8_H_8_O]^−^, 125.1002, 107.0114[M − H-GlcUA-C_8_H_8_O-CO_2_]^−^
M12	Naringenin-7*-O-*glucuronide ^b^	C_21_H_20_O_11_	11.4	447.0952 (0.9)	271.0607[M − H-GlcUA]^−^, 175.0243[M − H-NE]^−^, 151.0029[M − H-GlcUA-C_8_H_8_O]^−^, 119.0323[M − H-GlcUA-C_7_H_4_O_4_]^−^, 113.0248[M − H-NE-CO_2_-H_2_O]^−^, 85.0384[M − H-NE-CO_2_-H_2_O-CO]^−^
M13	Naringenin-4′*-O-*glucuronide ^b^	C_21_H_20_O_11_	11.7	447.0943 (-1.1)	429.0864[M − H-H_2_O]^−^, 385.0948[M − H-H_2_O-CO_2_]^−^, 271.0608[M − H-GlcUA]^−^, 175.0242[M − H-NE]^−^, 151.0025[M − H-GlcUA-C_8_H_8_O]^−^, 119.0354[M − H-GlcUA-C_7_H_4_O_4_]^−^, 113.0243[M − H-NE-CO_2_-H_2_O]^−^, 85.0311[M − H-NE-CO_2_-H_2_O-CO]^−^
	**Hesperetin metabolites**				
M14	Hesperetin-3′*-O-*sulfate ^b^	C_16_H_14_O_9_S	10.4	381.0301 (0.8)	301.0712[M − H-SO_3_]^−^, 286.0487[M − H-SO_3_-CH_3_]^−^, 177.0188[M − H-SO_3_-C_7_H_8_O_2_]^−^, 151.0029[M − H-SO_3_-C_9_H_10_O_2_]^−^, 107.0145[M − H-SO_3_-C_9_H_10_O_2_-CO_2_]^−^, 83.0308
M15	Hesperetin-7*-O-*sulfate	C_16_H_14_O_9_S	12.6	381.0311 (2.3)	301.0730[M − H-SO_3_]^−^, 286.0498[M − H-SO_3_-CH_3_]^−^, 242.0581, 199.0603, 164.0117, 151.0032[M − H-SO_3_-C_9_H_10_O_2_]^−^, 134.0373
M16	Hesperetin-7*-O-*glucuronide ^b^	C_22_H_22_O_12_	11.8	477.1083 (-2.2)	379.0833, 301.0737[M − H-GlcUA]^−^, 286.0489[M − H-GlcUA-CH_3_]^−^, 175.0242[M − H-HE]^−^, 113.0252[M − H-HE-CO_2_-H_2_O]^−^, 96.0085
M17	Hesperetin-3′*-O-*glucuronide ^b^	C_22_H_22_O_12_	12.3	477.1064 (1.1)	301.0734[M − H-GlcUA]^−^, 175.0226[M − H-HE]^−^, 113.0248[M − H-HE-CO_2_-H_2_O]^−^, 85.0355[M − H-HE-CO_2_-H_2_O-CO]^−^
	Eriodictyol metabolites				
M18	Eriodictyol*-O-*glucuronide	C_21_H_20_O_12_	10.9	463.0929 (2.3)	287.0563[M − H-GlcUA]^−^, 255.0668, 175.0233[M − H-EY]^−^, 151.0030[M − H-GlcUA-C_8_H_8_O_2_]^−^, 135.0451[M − H-GlcUA-C_7_H_4_O_4_]^−^, 113.0226[M − H-EY-CO_2_-H_2_O]^−^, 85.0326[M − H-EY-CO_2_-H_2_O-CO]^−^
M19	Eriodictyol*-O-*sulfate	C_15_H_12_O_9_S	12.5	367.0169 (4.2)	287.0572[M − H-SO_3_]^−^, 151.0037[M − H-SO_3_-C_8_H_8_O_2_]^−^, 135.0451[M − H-SO_3_-C_7_H_4_O_4_]^−^, 107.0144[M − H-SO_3_-C_8_H_8_O_2_-CO_2_]^−^
	**Apigenin metabolites**				
M20	Apigenin*-O-*glucuronide	C_21_H_18_O_11_	12.6	445.2089 (0.6)	269.0463[M − H-GlcUA]^−^, 175.0240[M − H-AE]^−^, 151.0028[M − H-GlcUA-C_8_H_6_O]^−^, 117.0240[M − H-GlcUA-C_7_H_4_O_4_]^−^, 113.0250[M − H-AE-CO_2_-H_2_O]^−^, 85.0312[M − H-AE-CO_2_-H_2_O-CO]^−^

^a^ The losses are: Glc = glucose moiety, GlcUA = glucuronyl moiety, NE = naringenin, NEG = naringenin glucuronide, HE = hesperetin, EY = eriodictyol, AE = apigenin. ^b^ Confirmation in comparison with authentic standards.

**Table 3:** Quantification of metabolites of naringenin and hesperetin in the urine after the ingestion of 250 mL ECG extract containing glycosides of naringenin (346 μmol), and hesperetin (0.08 μmol).

No.	Metabolites (nmol) ^a^	0–4 h	4–8 h	8–12 h	12–24 h	24–36 h	36–48 h	Total
M1	Naringenin	26.4 ± 8.5	276 ± 62	227 ± 39	182 ± 61	13.5 ± 6.1	<LD ^b^	725 ± 80
M2	Naringenin-4′,7*-O-*diglucuronide	0.6 ± 0.3	7.2 ± 2.5	146 ± 46	32.3 ± 6.1	<LD	<LD	186 ± 50
M3	Naringenin-5,7*-O-*diglucuronide	1.2 ± 0.5	228 ± 92	124 ± 20	26.1 ± 8.5	2.0 ± 0.9	<LD	382 ± 109
M4	Naringenin-4′,5*-O-*diglucuronide	<LD	22.0 ± 9.8	7.9 ± 3.0	<LD	<LD	<LD	29.9 ± 12.8
M5	Naringenin*-O-*glucoside*-O-*sulfate	<LD	<LD	7.4 ± 2.2	<LD	<LD	<LD	7.4 ± 2.2
M6	Naringenin*-O-*glucoside*-O-*sulfate	<LD	328 ± 104	329 ± 68	119 ± 46	13.4 ± 6.0	<LD	789 ± 126
M7	Naringenin*-O-*glucoside*-O-*glucuronide	<LD	295 ± 92	242 ± 34	80.1 ± 29.7	7.1 ± 3.2	<LD	624 ± 109
M8	Naringenin*-O-*glucuronide*-O-*sulfate	<LD	429 ± 174	221 ± 24	55.9 ± 19.7	7.1 ± 3.2	<LD	713 ± 179
M9	Naringenin-4′*-O-*sulfate	<LD	93.1 ± 26.8	114 ± 19	20.0 ± 6.9	3.0 ± 1.3	<LD	230 ± 22
M10	Naringenin-7*-O-*sulfate	<LD	73.4 ± 19.2	86.0 ± 15.4	15.8 ± 6.1	<LD	<LD	175 ± 20
M11	Naringenin-5*-O-*glucuronide	<LD	26.8 ± 10.2	18.3 ± 2.2	5.2 ± 1.7	<LD	<LD	50.4 ± 10.9
M12	Naringenin-7*-O-*glucuronide	43.8 ± 6.8	3113 ± 860	2564 ± 302	829 ± 283	87.4 ± 32.3	5.8 ± 2.6	8296 ± 986
M13	Naringenin-4′*-O-*glucuronide	62.2 ± 8.9	3590 ± 885	3450 ± 344	1068 ± 322	115 ± 33	10.1 ± 3.0	6643 ± 806
	Total naringenin metabolites	134 ± 21	8483 ± 2303	7536 ± 739	2434 ± 785	249 ± 85	15.8 ± 5.3	18852 ± 2370
	*% Recovery*	0.039 ± 0.006	2.45 ± 0.67	2.18 ± 0.21	0.70 ± 0.23	0.072 ± 0.025	0.005 ± 0.002	5.45 ± 0.68
M14	Hesperetin-3′*-O-*glucuronide	<LD	2.8 ± 1.2	1.7 ± 0.8	<LD	<LD	<LD	4.5 ± 2.0
M15	Hesperetin-7*-O-*glucuronide	3.9 ± 1.8	7.0 ± 3.1	15.0 ± 1.9	2.8 ± 1.2	<LD	<LD	28.7 ± 5.7
M16	Hesperetin -7*-O-*sulfate	<LD	1.5 ± 0.7	4.1 ± 0.8	<LD	<LD	<LD	5.6 ± 0.6
M17	Hesperetin-3′*-O-*sulfate	<LD	1.5 ± 0.7	4.9 ± 0.6	1.7 ± 0.7	<LD	<LD	8.1 ± 1.6
	Total hesperetin metabolites	3.9 ± 1.8	12.8 ± 5.7	25.8 ± 2.7	4.4 ± 1.3	<LD	<LD	47.0 ± 9.5
	*% Recovery*	4.9 ± 2.2	16.0 ± 7.2	32.2 ± 3.4	5.6 ± 1.6	<LD	<LD	58.7 ± 11.9

^a^ Data expressed as mean ± standard error (*n* = 5). ^b^ <LD (limit of detection).

## Supplementary Materials

The following are available online at http://www.mdpi.com/1420-3049/23/4/895/s1.
