# Supplementary material for: UFLC-Q-TOF-MS/MS-Based Screening and Identification of Flavonoids and Derived Metabolites in Human Urine after Oral Administration of Exocarpium Citri Grandis Extract
Source: Molecules. 2018 Apr 12;23(4):895. doi: 10.3390/molecules23040895 (PMC6017061; doi:10.3390/molecules23040895)

Electronic Supplementary Material

Journal name

Molecules

Article title

UFLC-Q-TOF-MS/MS-based screening and identification of flavonoids and derived metabolites in human urine after oral administration of Exocarpium *Citri grandis* extract

Contents

Part I. Structures and product ion spectra of identified flavonoids in Exocarpium *Citri grandis* extract *(Page 2-19)*

Part II. Structures and product ion spectra of identified metabolites in urine after the consumption of 250 mL Exocarpium *Citri grandis* extract *(Page 20-36)*

Part III. Detailed information, structures, and product ion spectra of identified phenolic catabolites in urine after the consumption of 250 mL Exocarpium *Citri grandis* extract *(Page 37-71)*

Part IV. Structures and product ion spectra of naringin-d4 (stable isotope labeled internal standard) *(Page 72)*

Part I. Structures and product ion spectra of identified flavonoids in Exocarpium *Citri grandis* extract

F1. Vicenin-2 (RT=9.5 min)

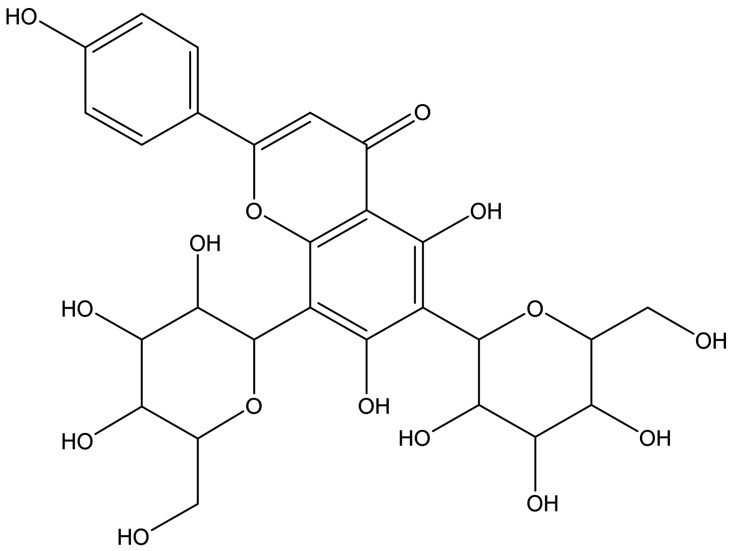

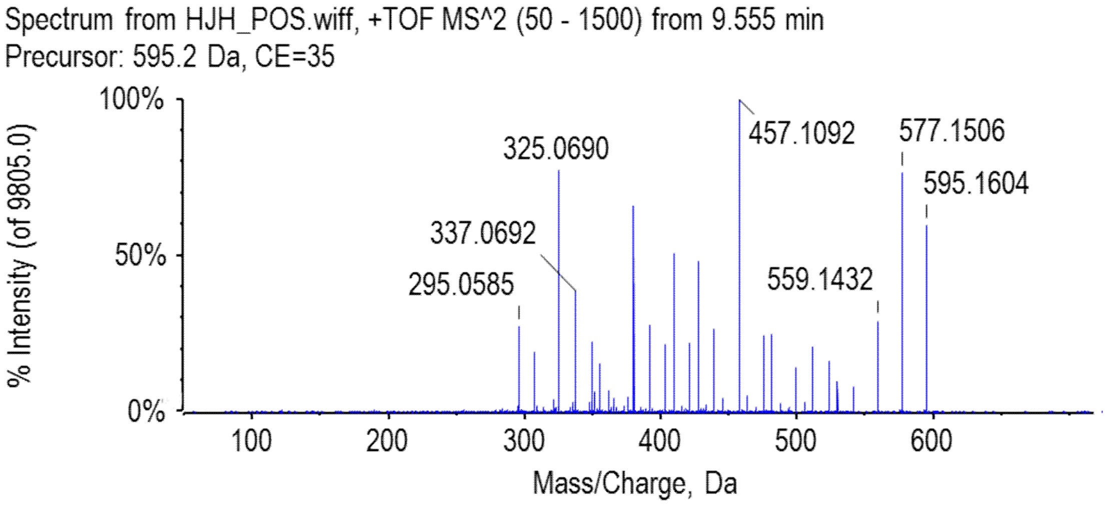

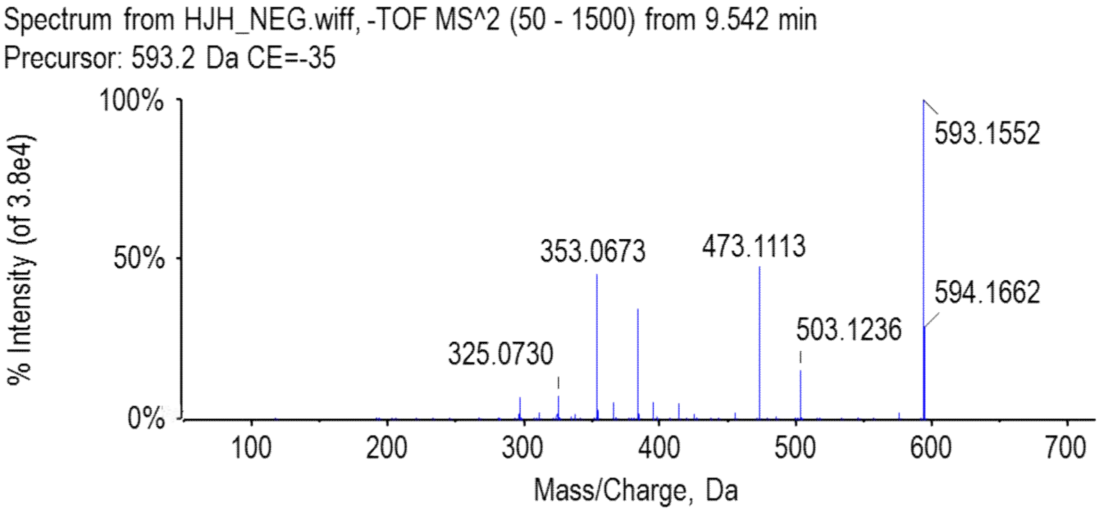

F2. Rhoifolin (RT=12.1 min)

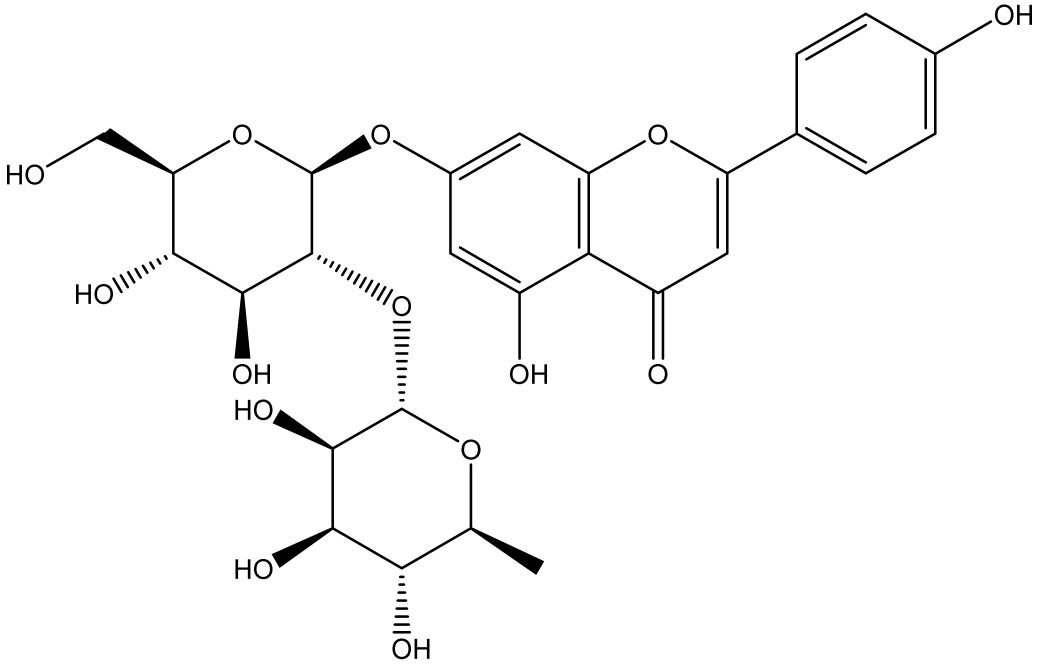

Spectrum from HJH_POS.wiff, +TOF MS^2 (50 - 1500) from 12.058 min
Precursor: 579.2 Da, CE=35

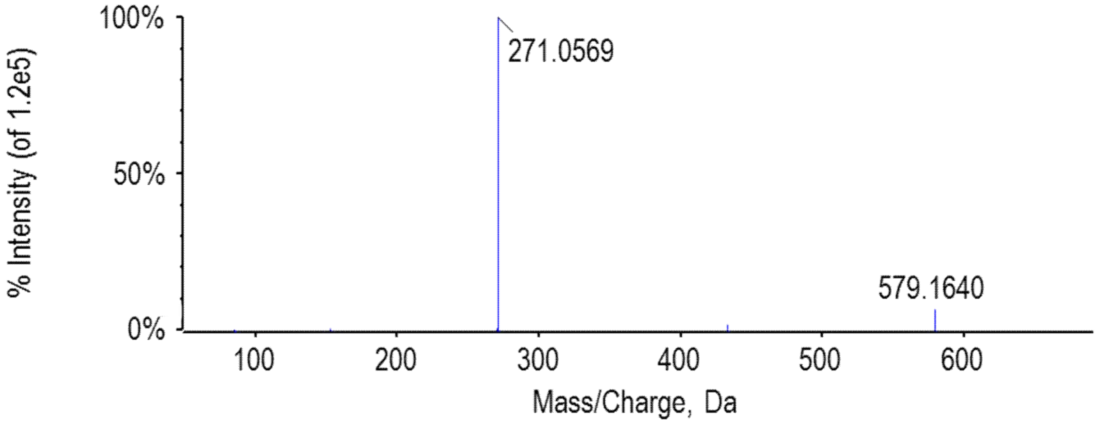

Spectrum from HJH_NEG.wiff, -TOF MS^2 (50 - 1500) from 12.060 min
Precursor: 577.2 Da CE=-35

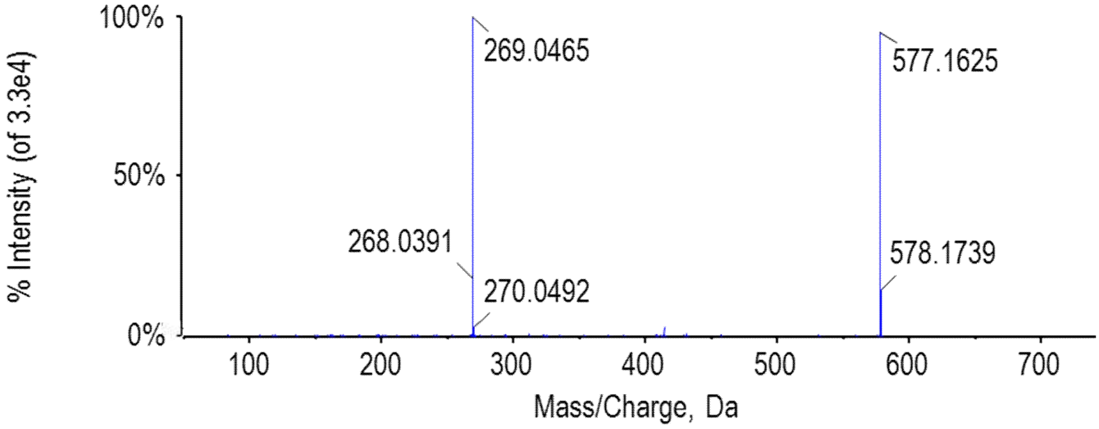

F3. Apigenin (RT=14.9 min)

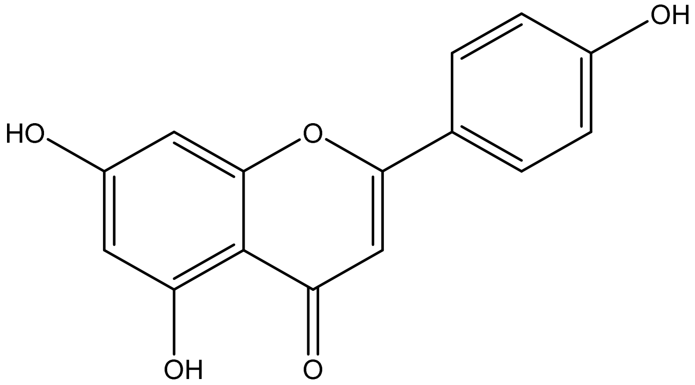

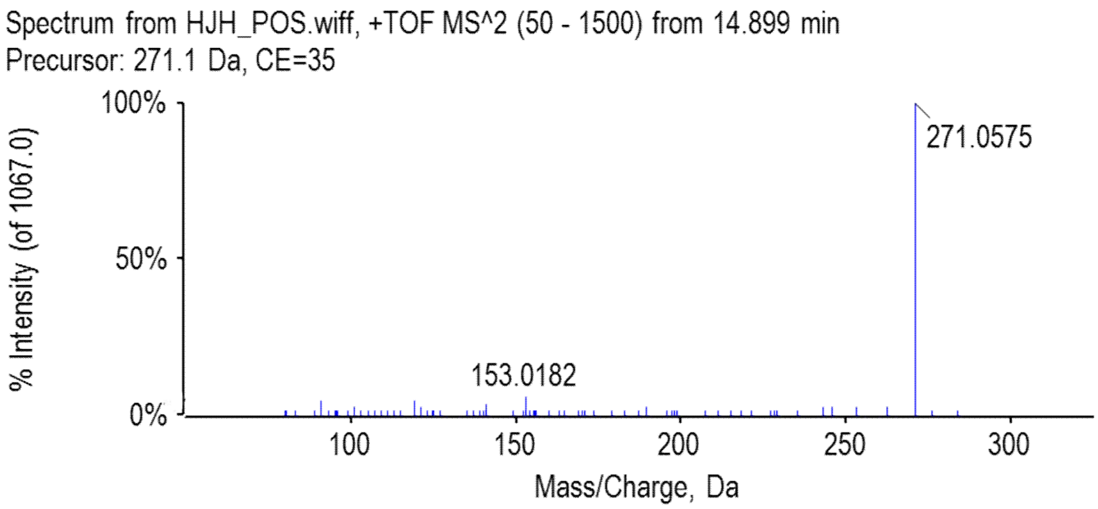

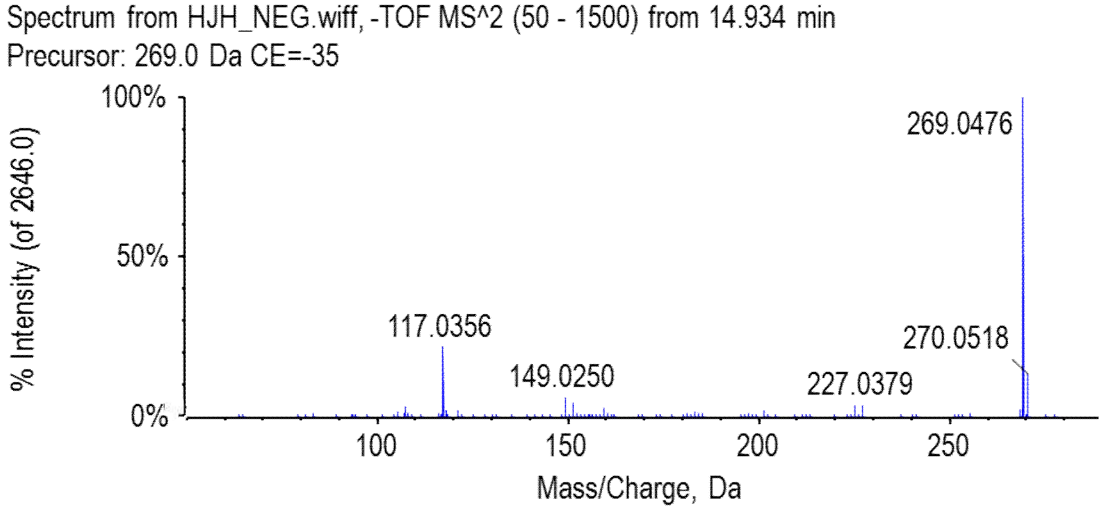

F4. Narirutin (RT=9.7 min)

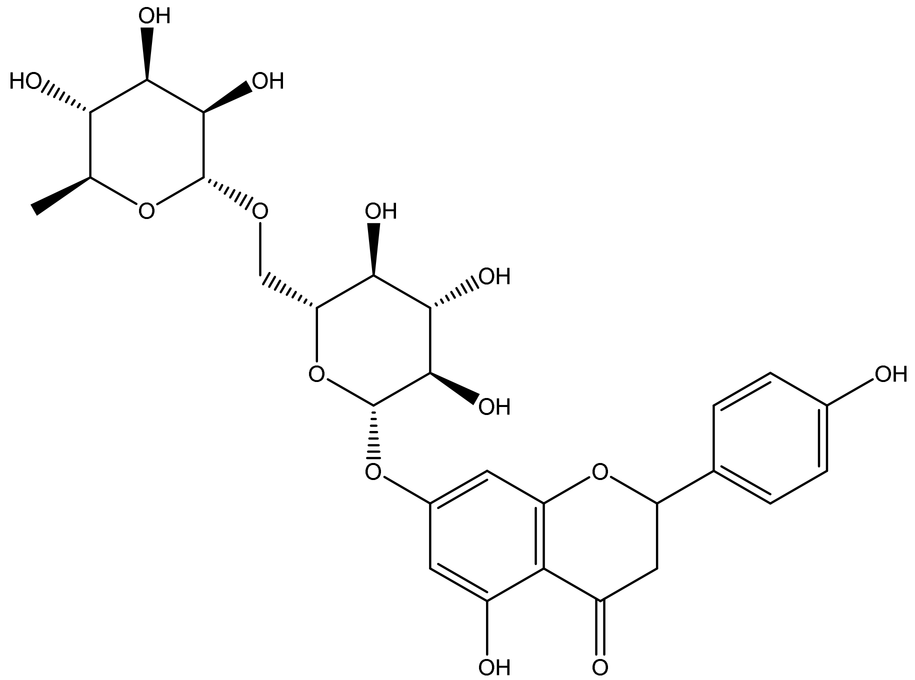

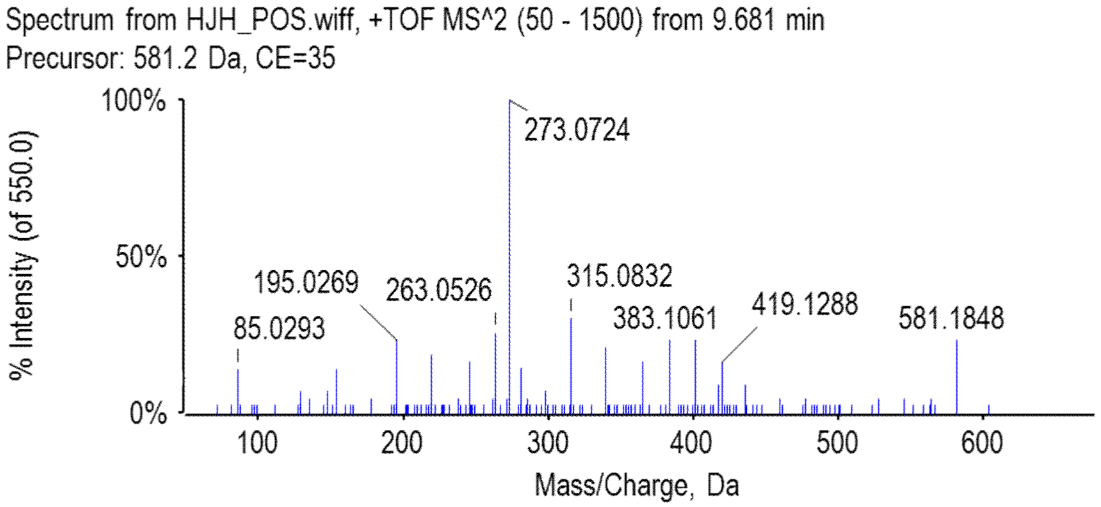

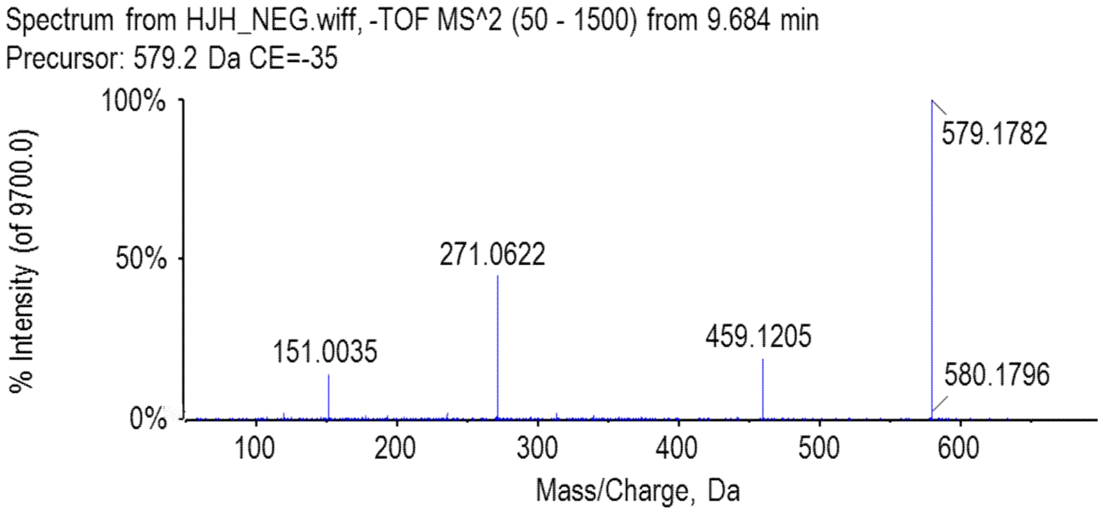

F5. Narirutin-4'-O-glucoside (RT=9.7 min)

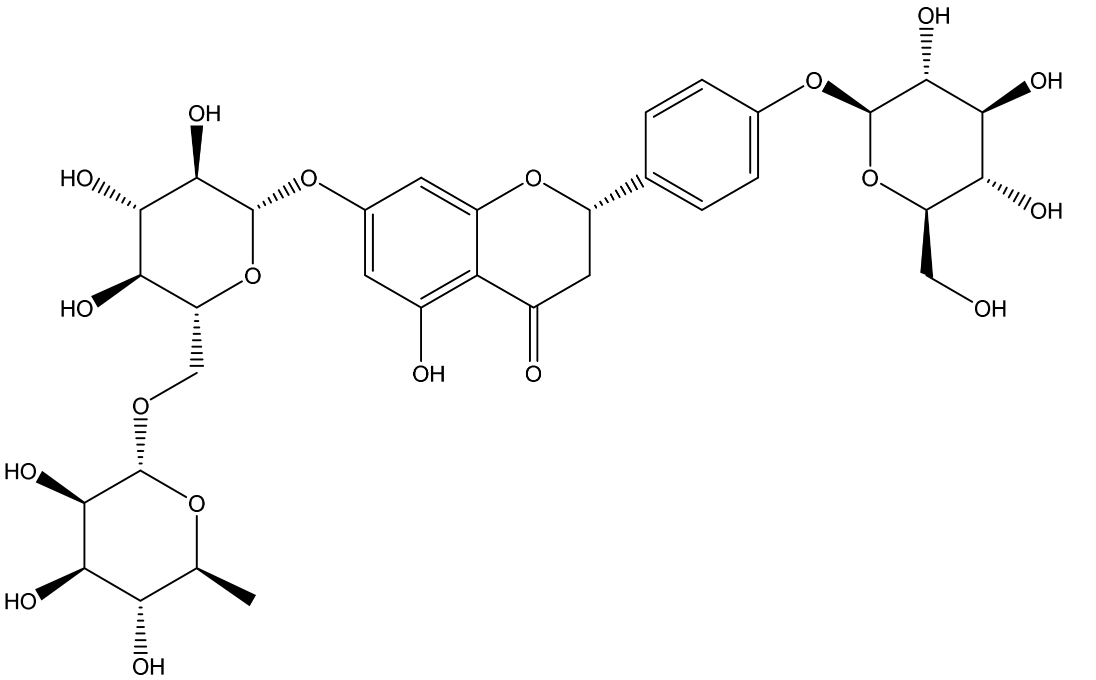

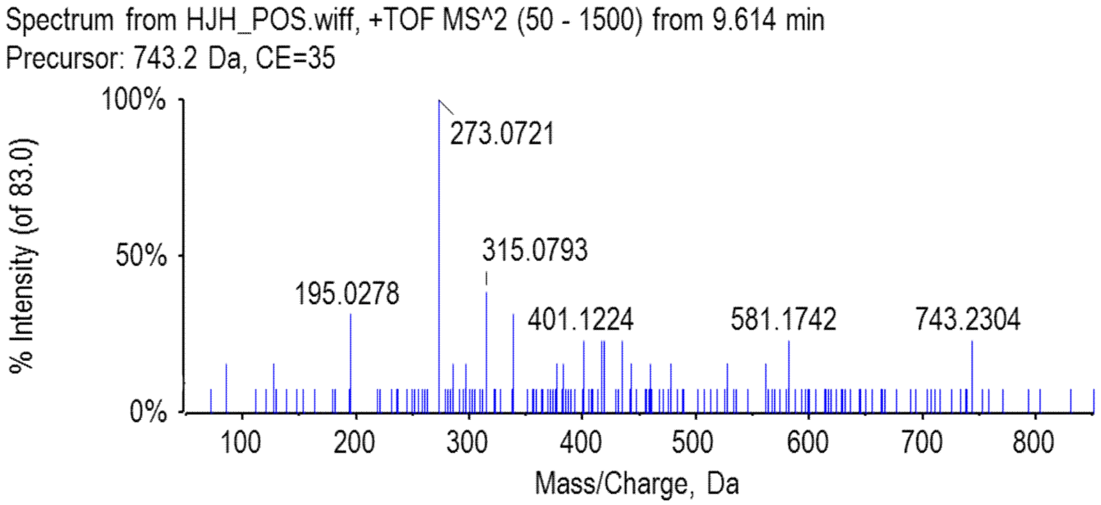

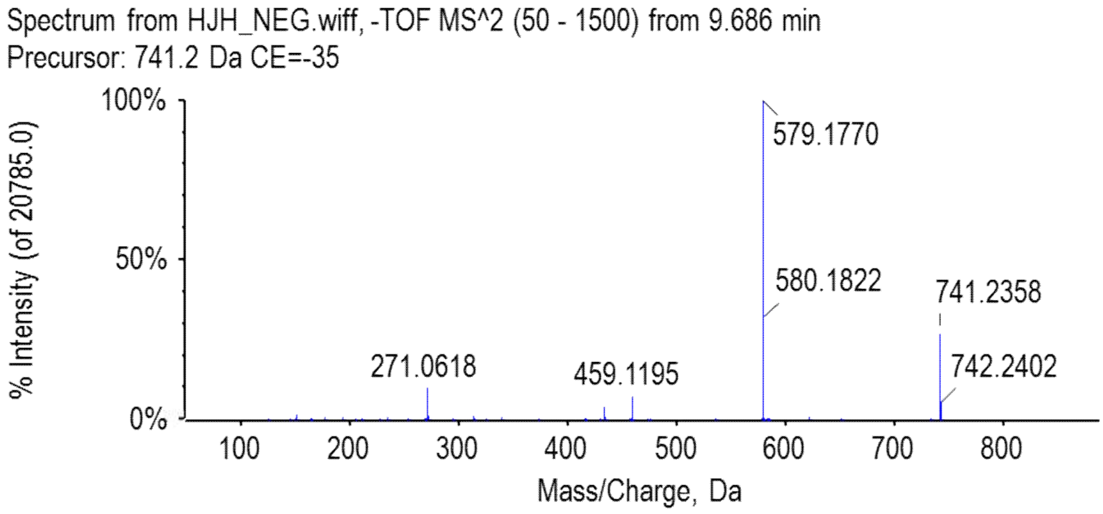

F6. Naringin-4'-O-glucoside (RT=10.4 min)

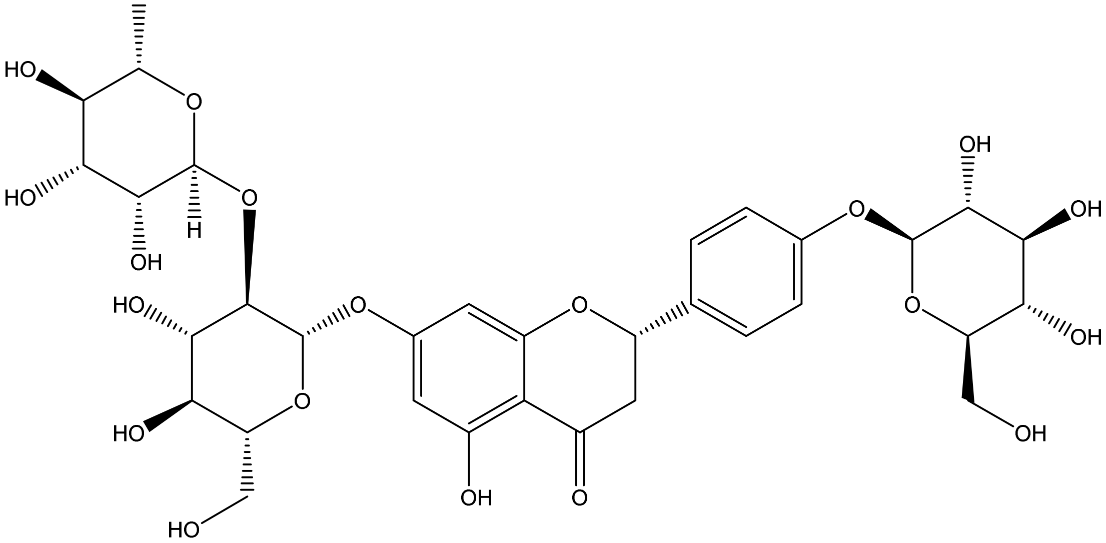

Spectrum from HJH_POS.wiff, +TOF MS^2 (50 - 1500) from 10.297 min
Precursor: 743.2 Da, CE=35

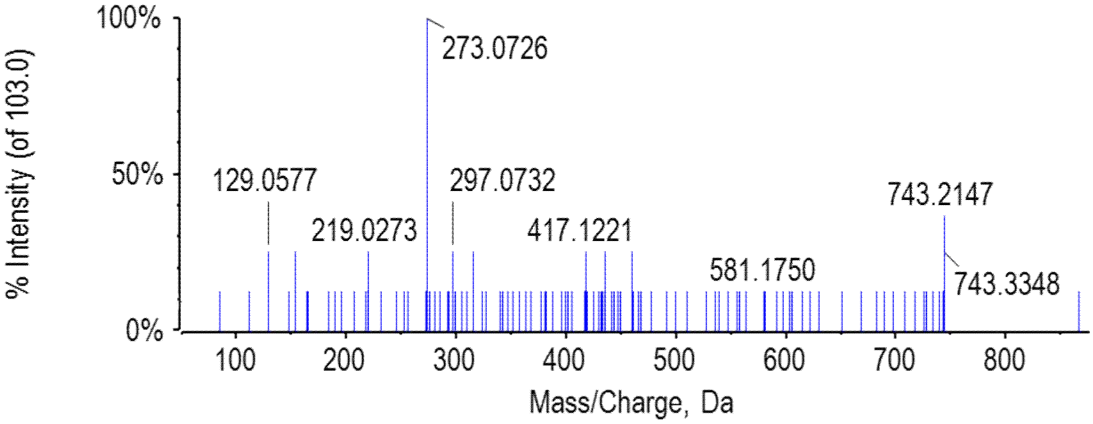

Spectrum from HJH_NEG.wiff, -TOF MS^2 (50 - 1500) from 10.365 min
Precursor: 741.2 Da CE=-35

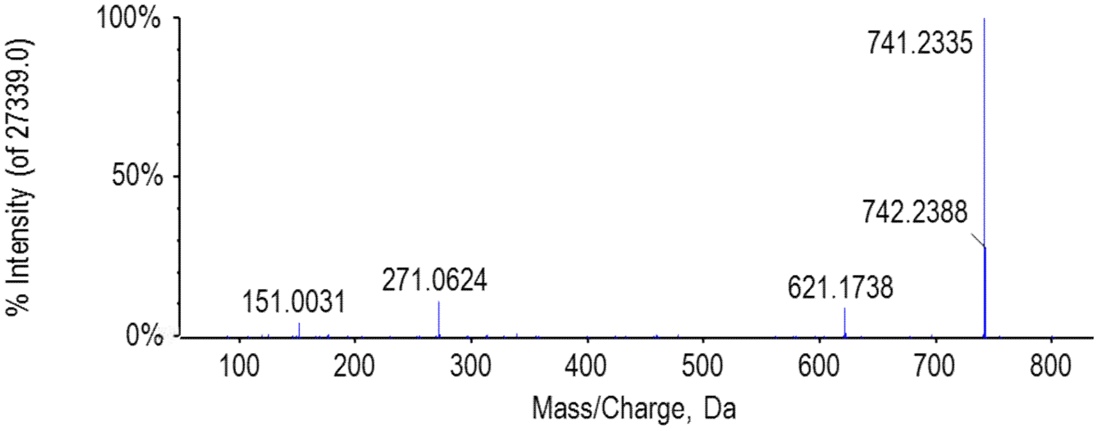

F7. Naringin (RT=11.4 min)

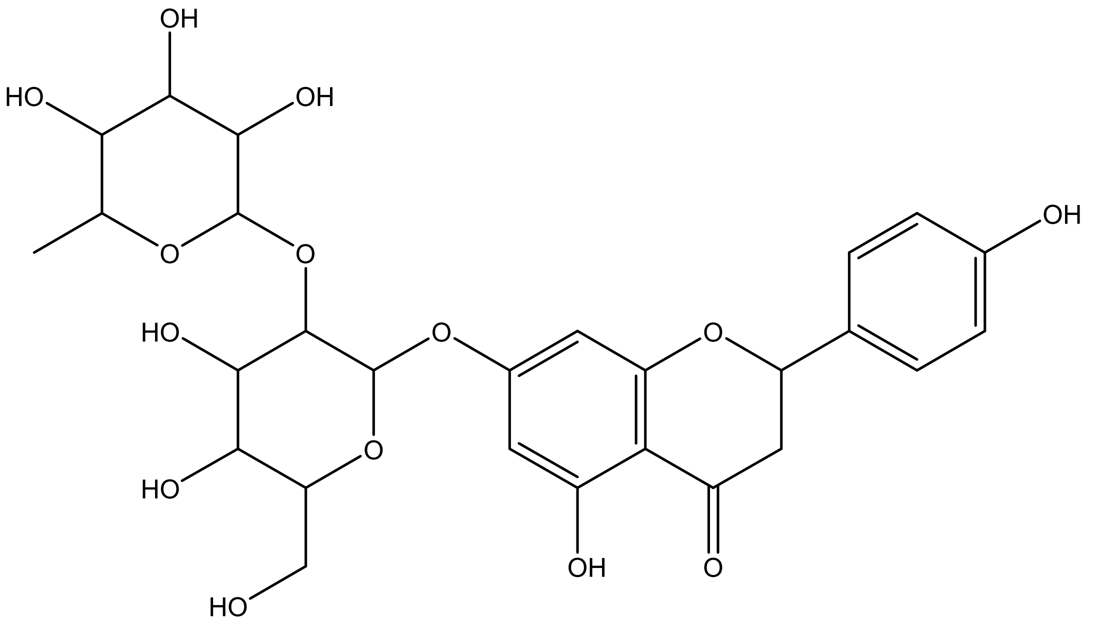

Spectrum from HJH_POS.wiff, +TOF MS^2 (50 - 1500) from 11.436 min
Precursor: 581.2 Da, CE=35

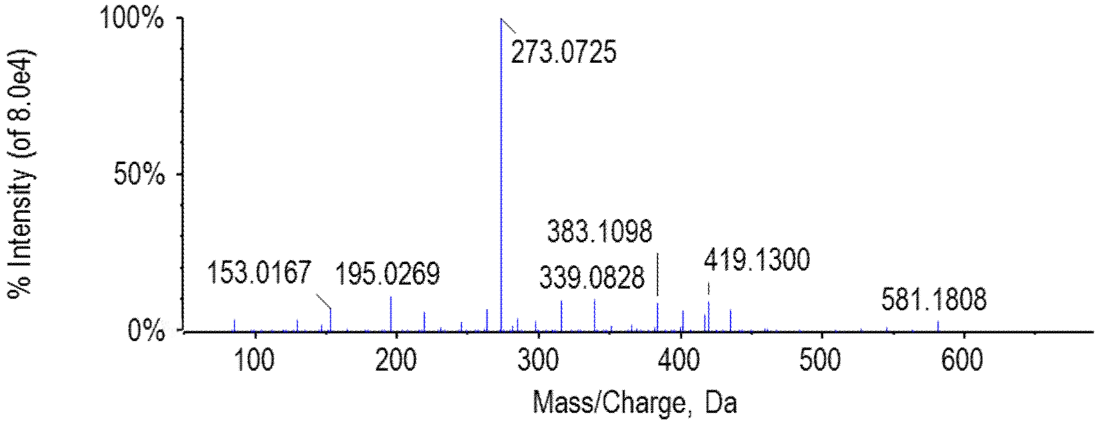

Spectrum from HJH_NEG.wiff, -TOF MS^2 (50 - 1500) from 11.399 min
Precursor: 579.2 Da CE=-35

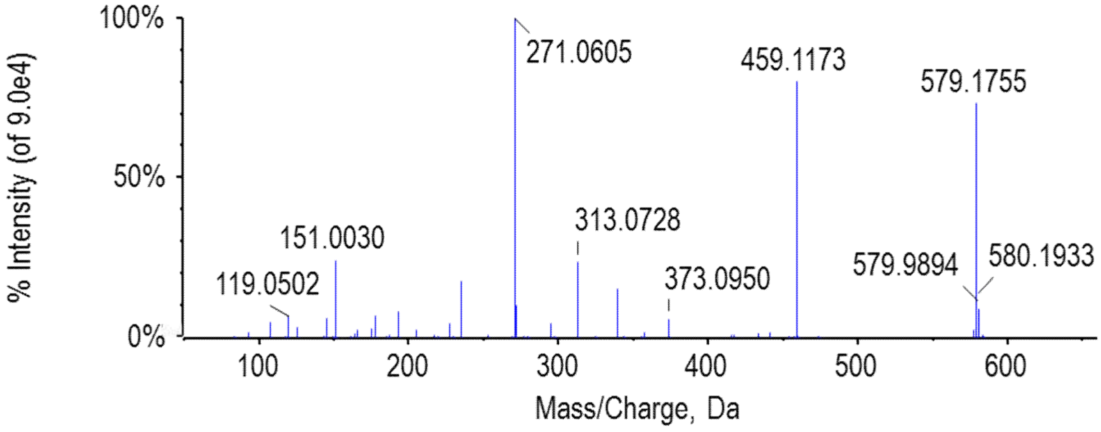

F8. Melitidin (RT=12.4 min)

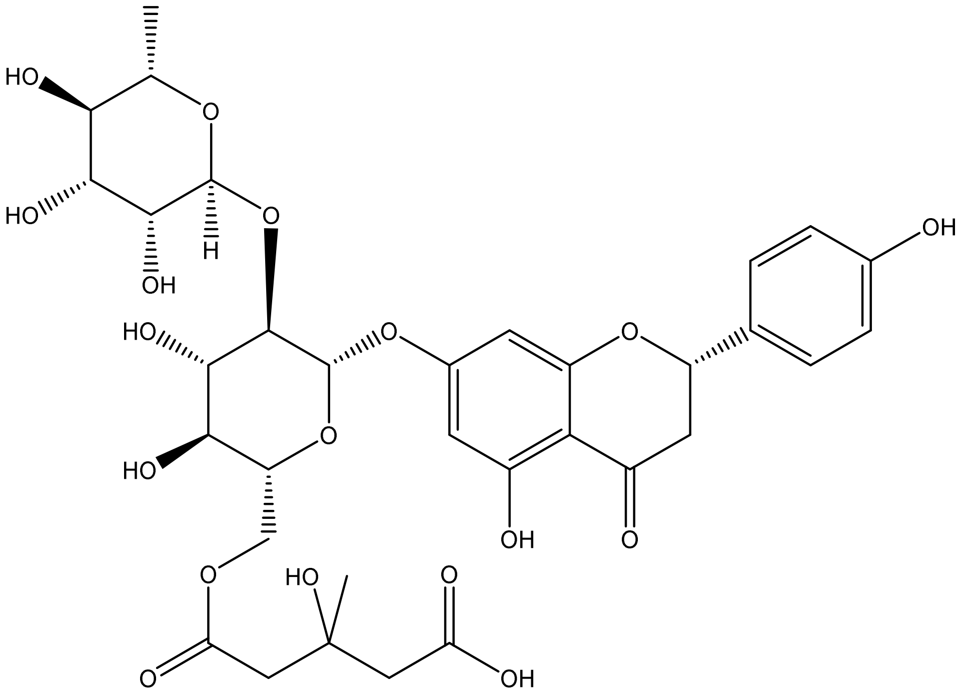

Spectrum from HJH_POS.wiff, +TOF MS^2 (50 - 1500) from 12.431 min
Precursor: 725.2 Da, CE=35

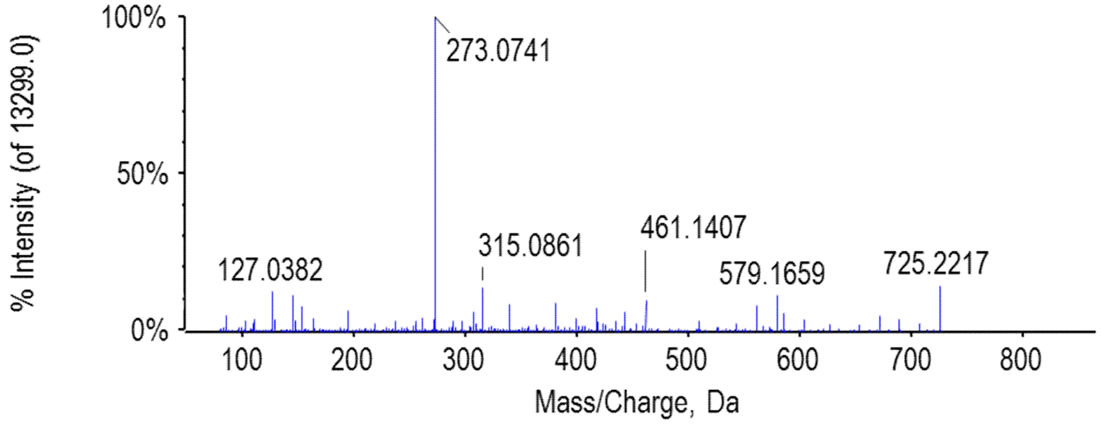

Spectrum from HJH_NEG.wiff, -TOF MS^2 (50 - 1500) from 12.402 min
Precursor: 723.2 Da CE=-35

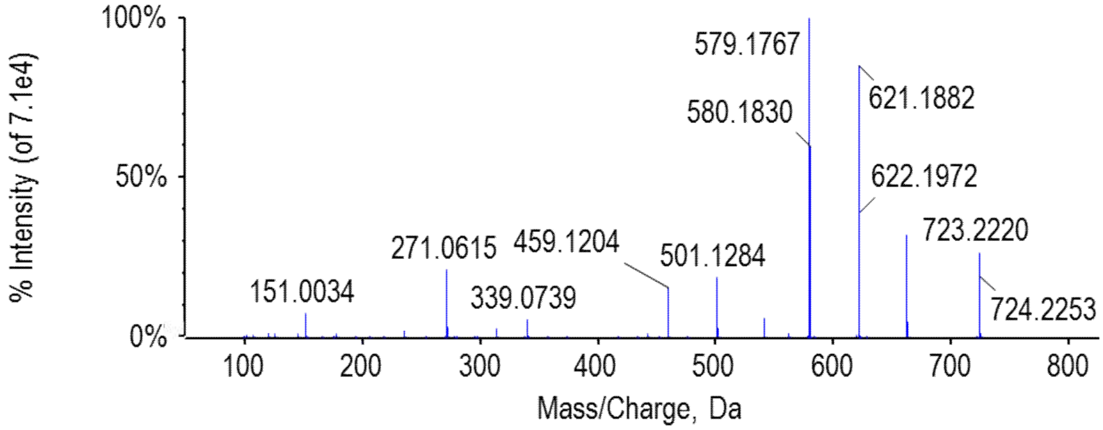

F9. Naringenin (RT=13.7 min)

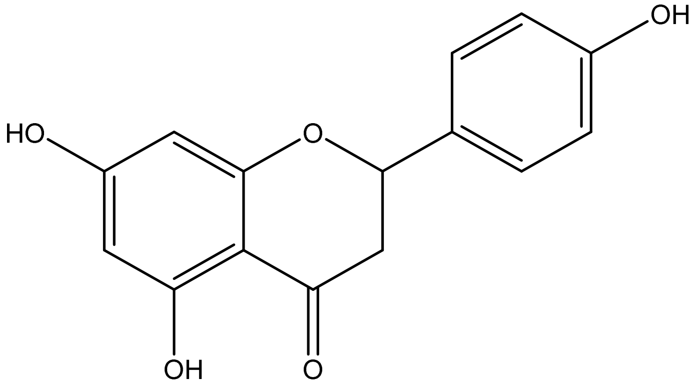

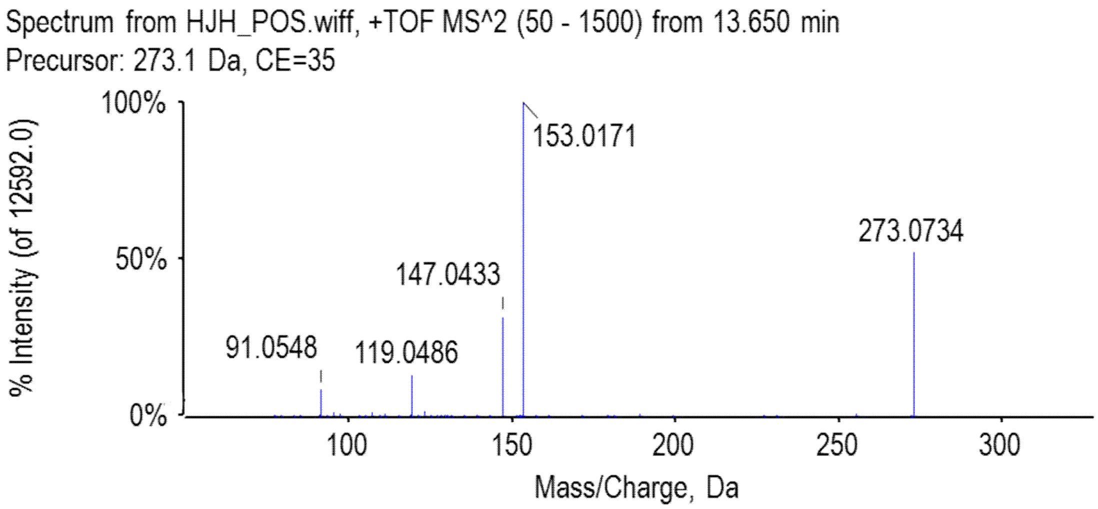

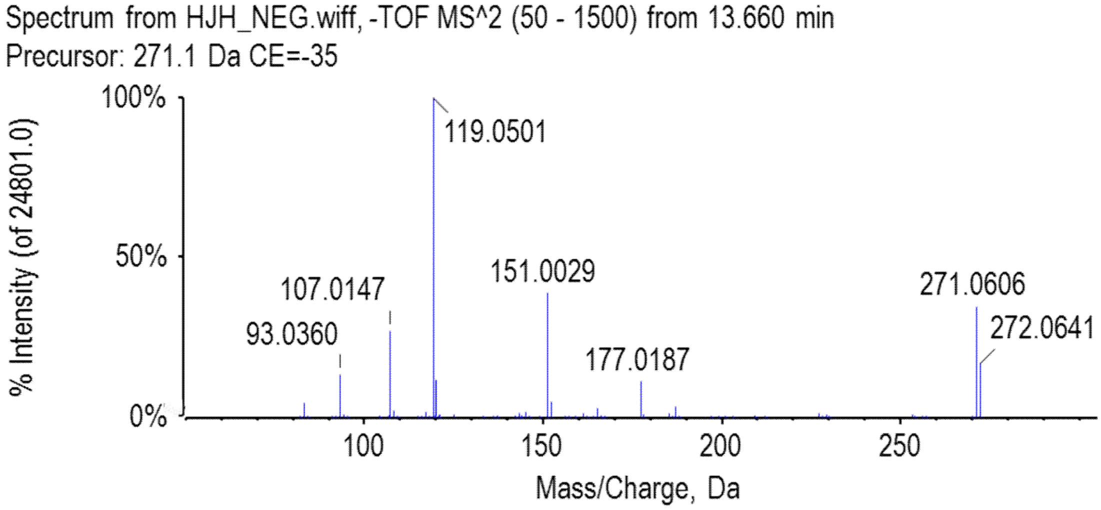

F10. Lucenin-2,4'-methyl ether (RT=9.8 min)

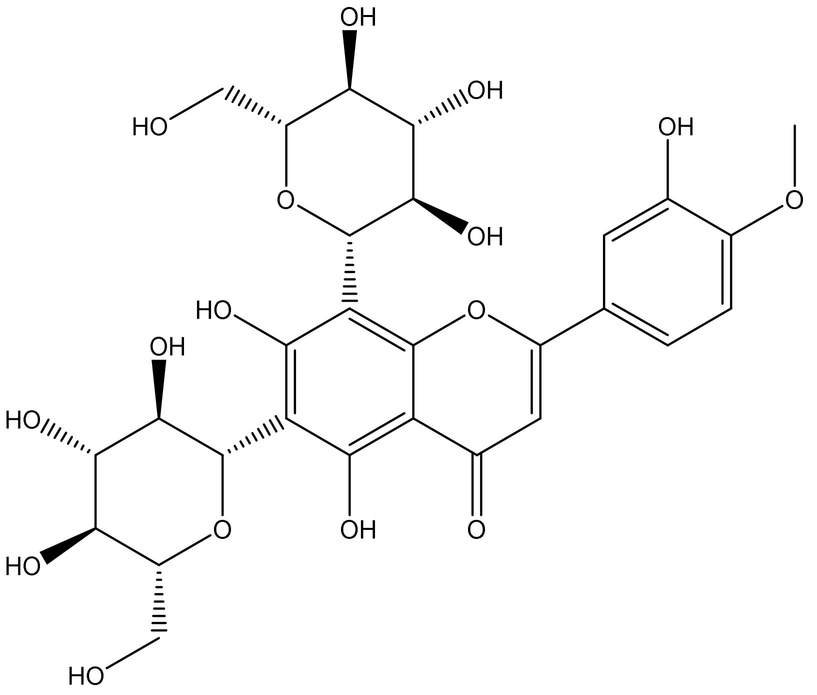

Spectrum from HJH_POS.wiff, +TOF MS^2 (50 - 1500) from 9.752 min
Precursor: 625.2 Da, CE=35

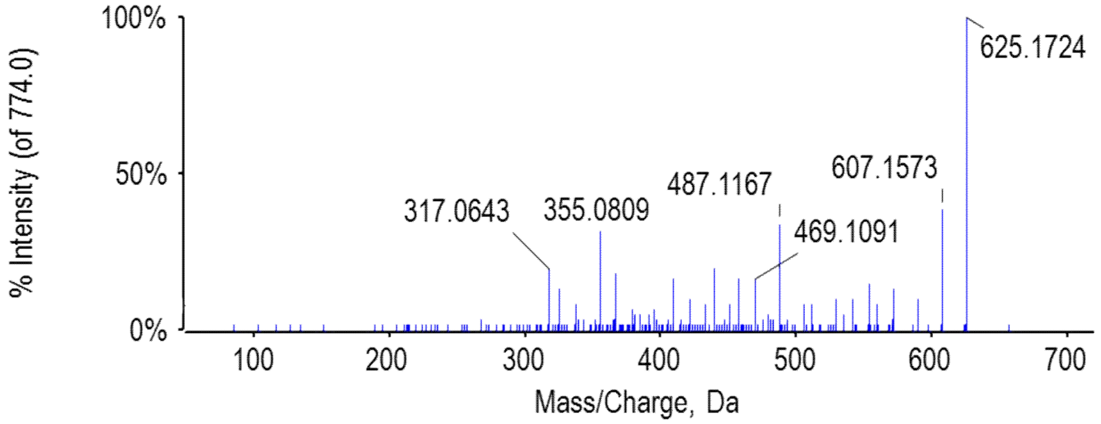

Spectrum from HJH_NEG.wiff, -TOF MS^2 (50 - 1500) from 9.791 min
Precursor: 623.2 Da CE=-35

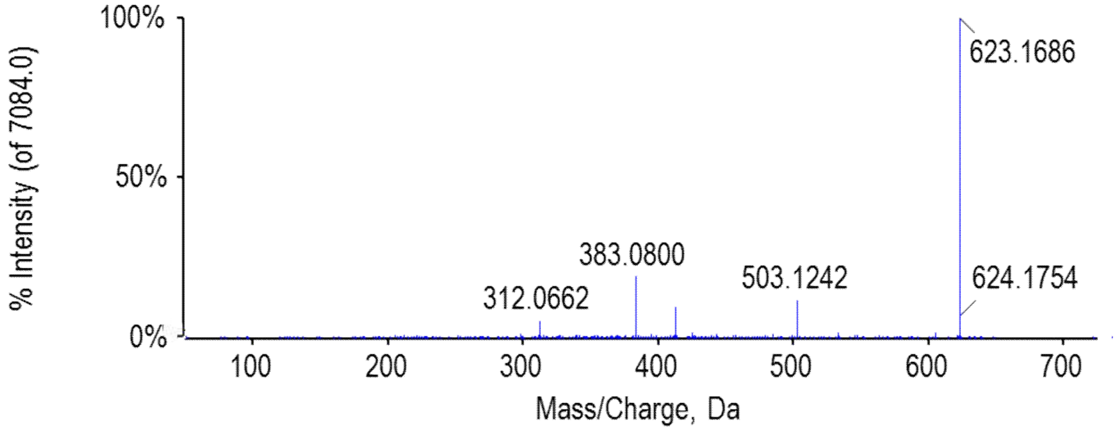

F11. Neodiosmin (RT=12.2 min)

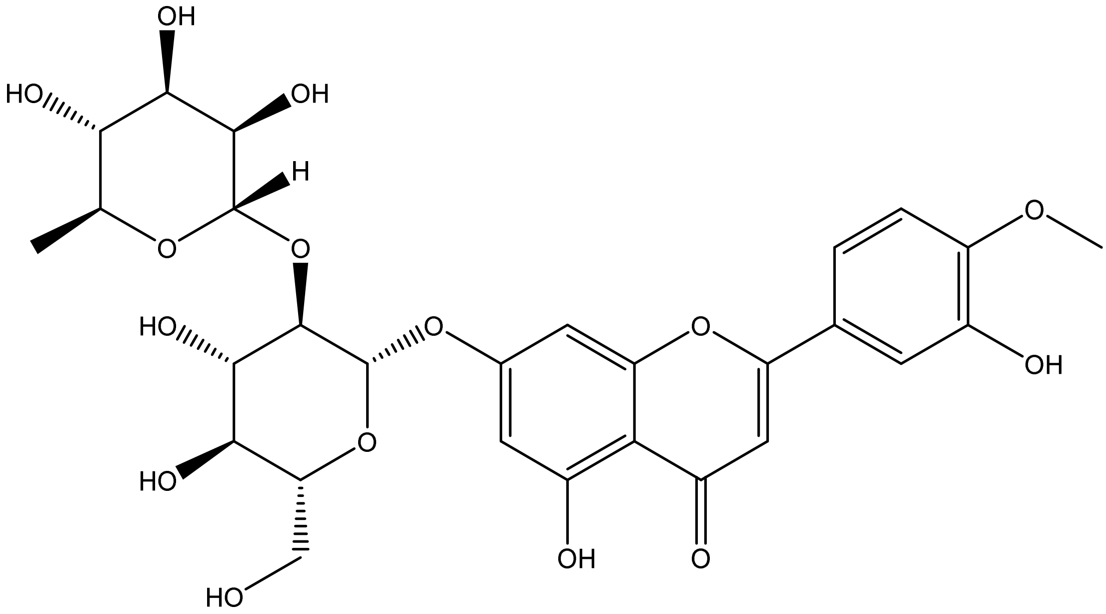

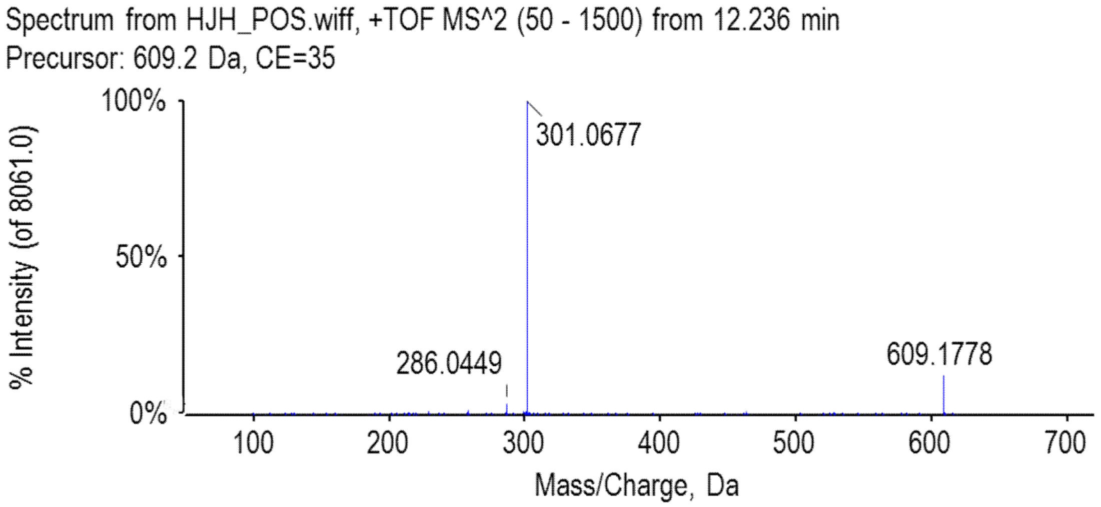

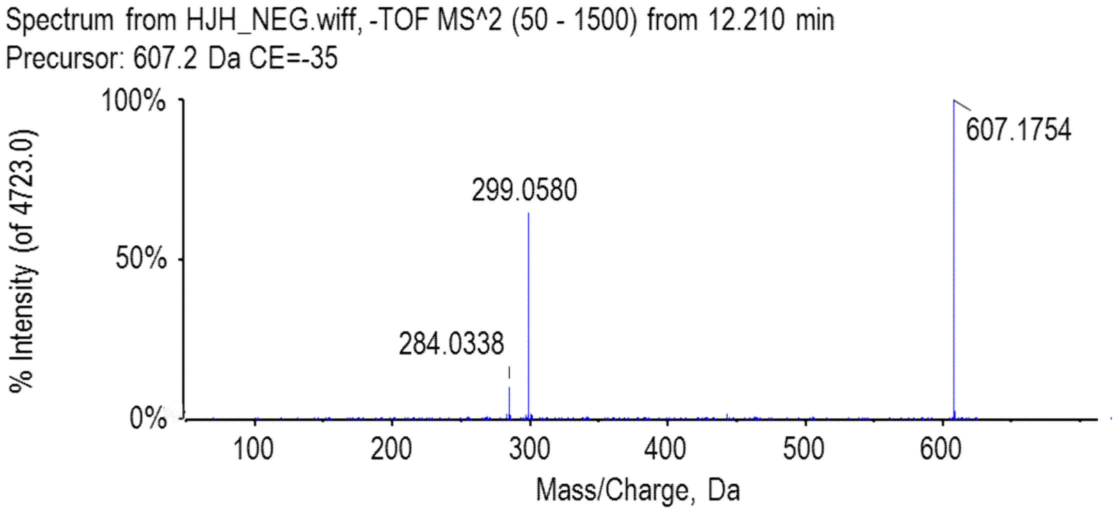

F12. Eriocitrin (RT=10.2 min)

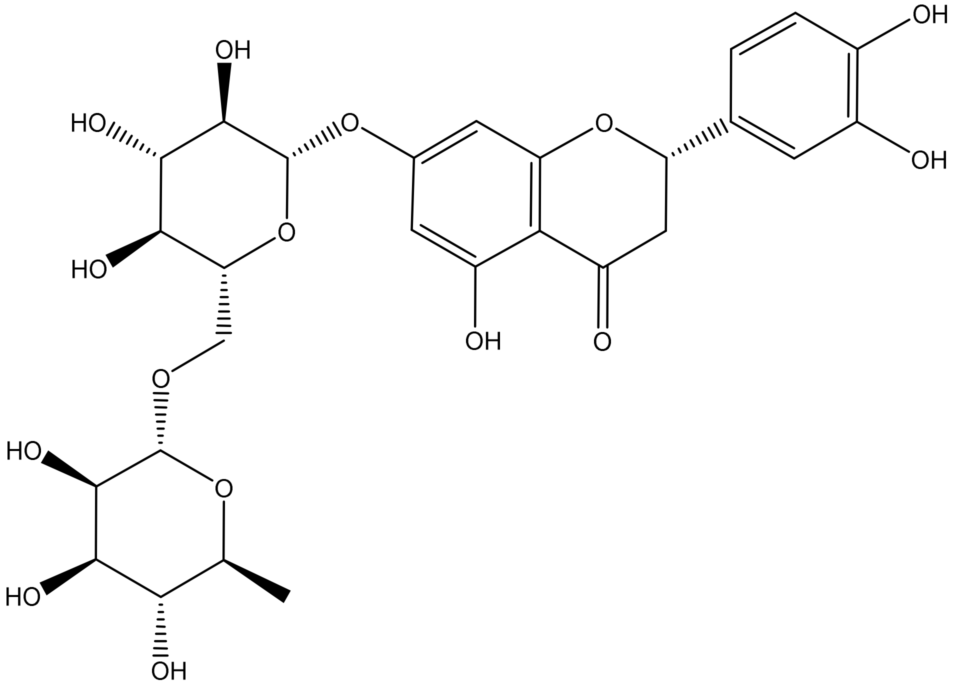

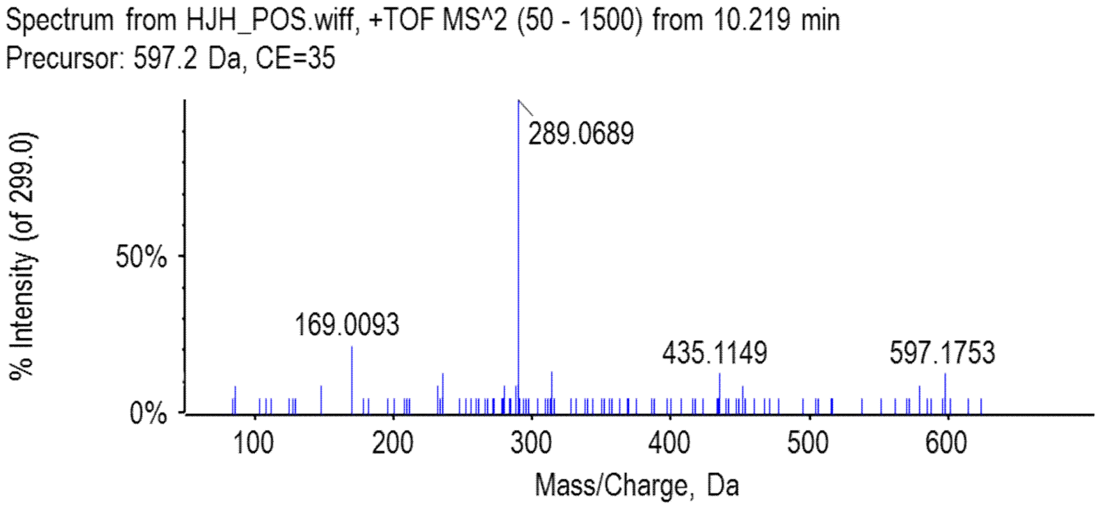

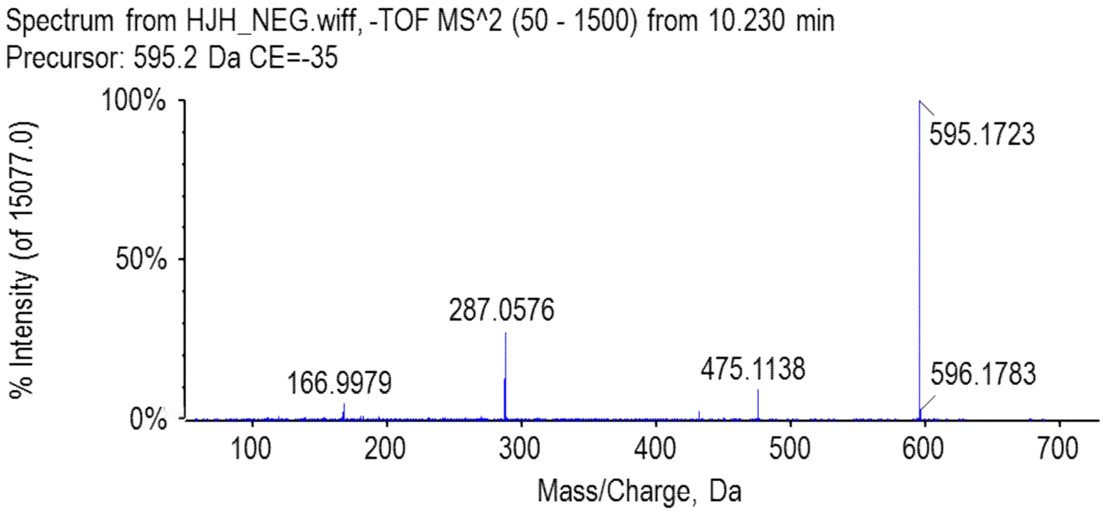

F13. Neoeriocitrin (RT=10.7 min)

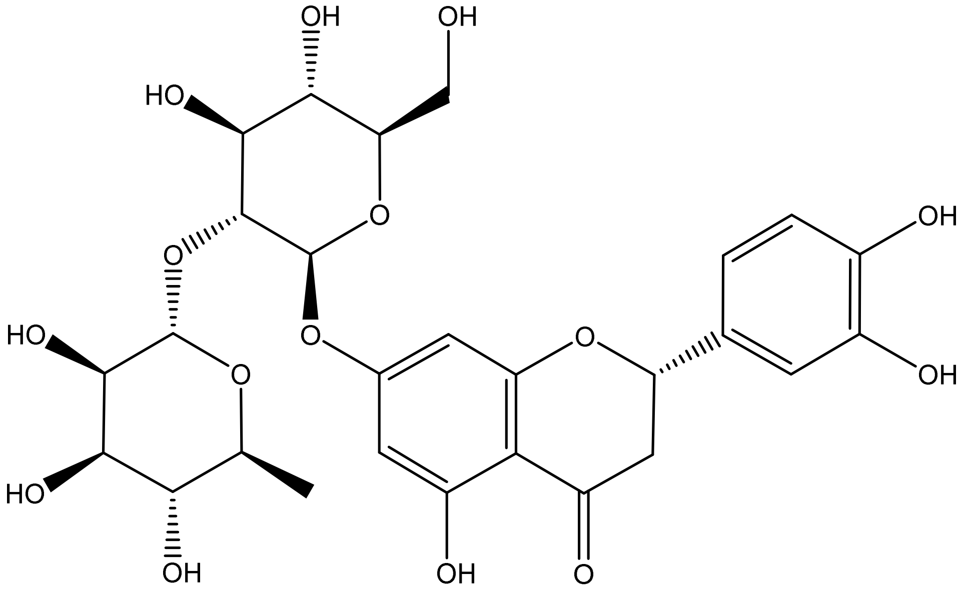

Spectrum from HJH_POS.wiff, +TOF MS^2 (50 - 1500) from 10.659 min
Precursor: 597.2 Da, CE=35

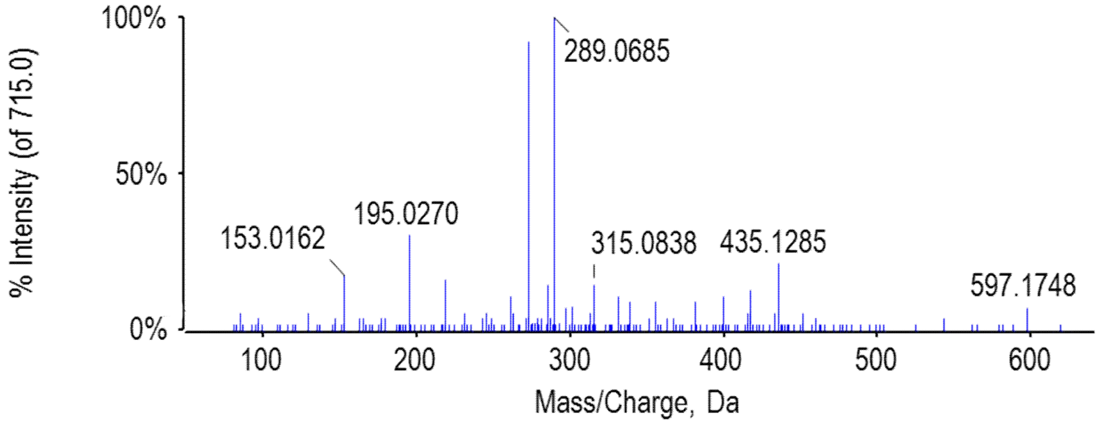

Spectrum from HJH_NEG.wiff, -TOF MS^2 (50 - 1500) from 10.627 min
Precursor: 595.2 Da CE=-35

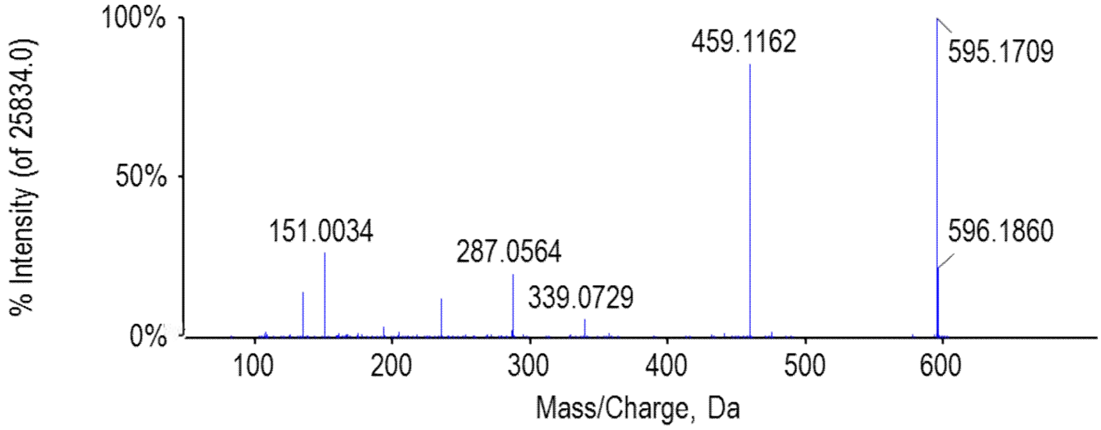

F14. Luteolin-6-C-glucoside (RT=10.5 min)

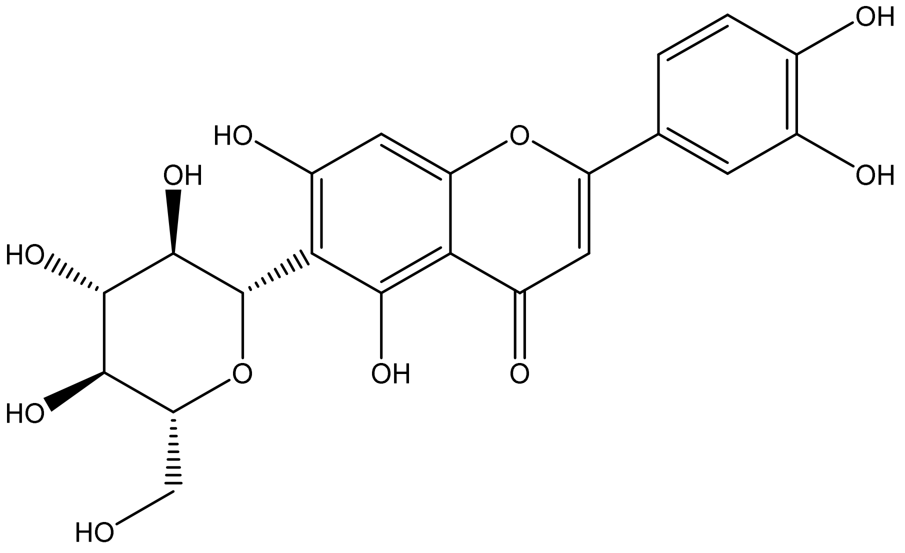

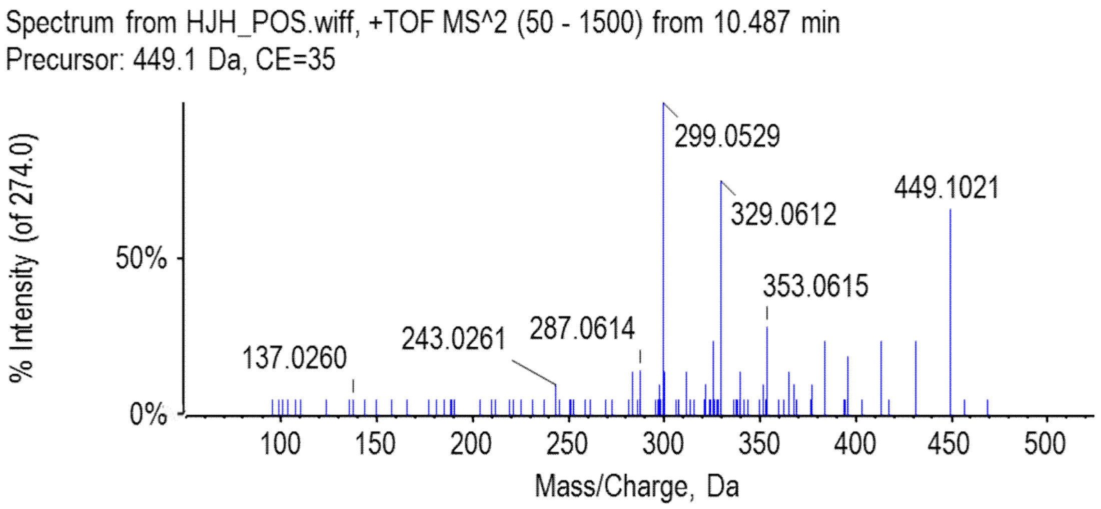

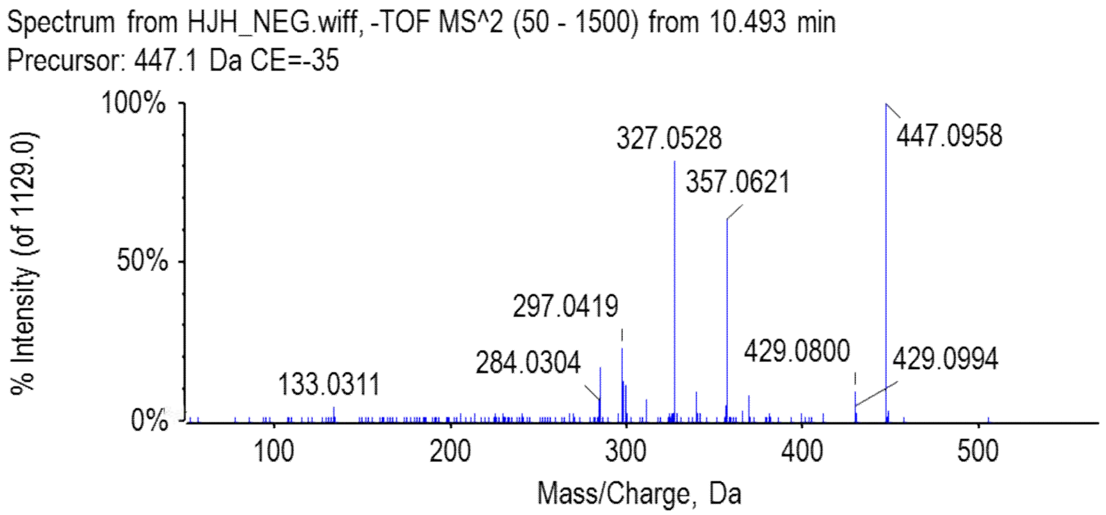

F15. Veronicastroside (RT=11.9 min)

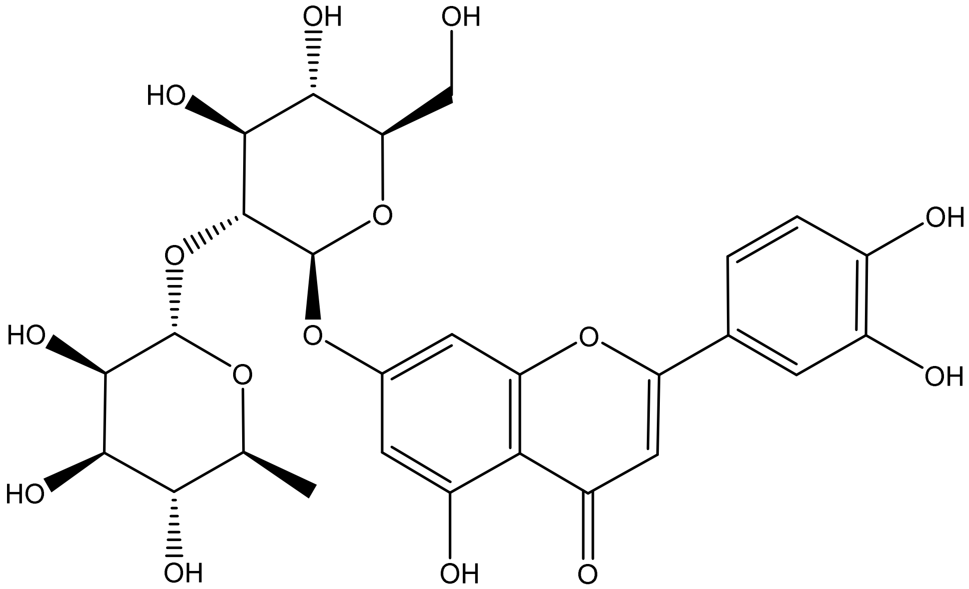

Spectrum from HJH_POS.wiff, +TOF MS^2 (50 - 1500) from 11.861 min
Precursor: 595.2 Da, CE=35

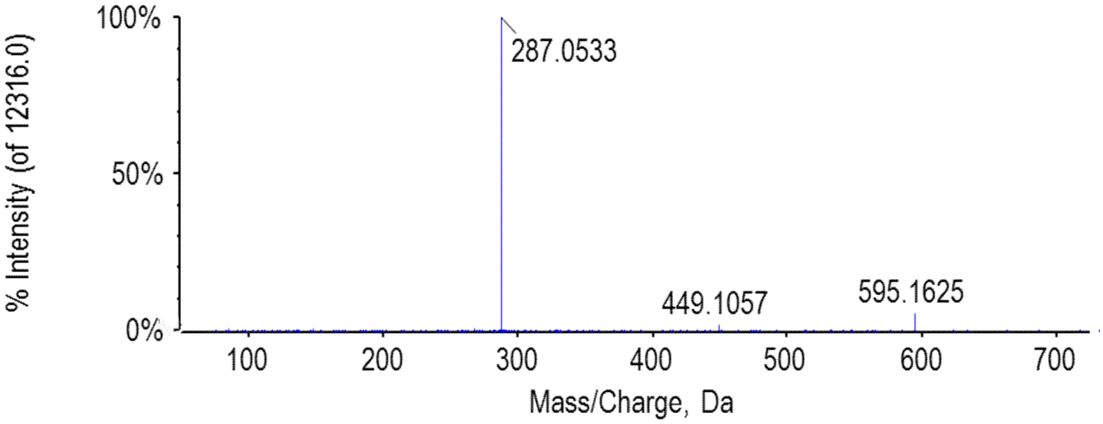

Spectrum from HJH_NEG.wiff, -TOF MS^2 (50 - 1500) from 11.887 min
Precursor: 593.2 Da CE=-35

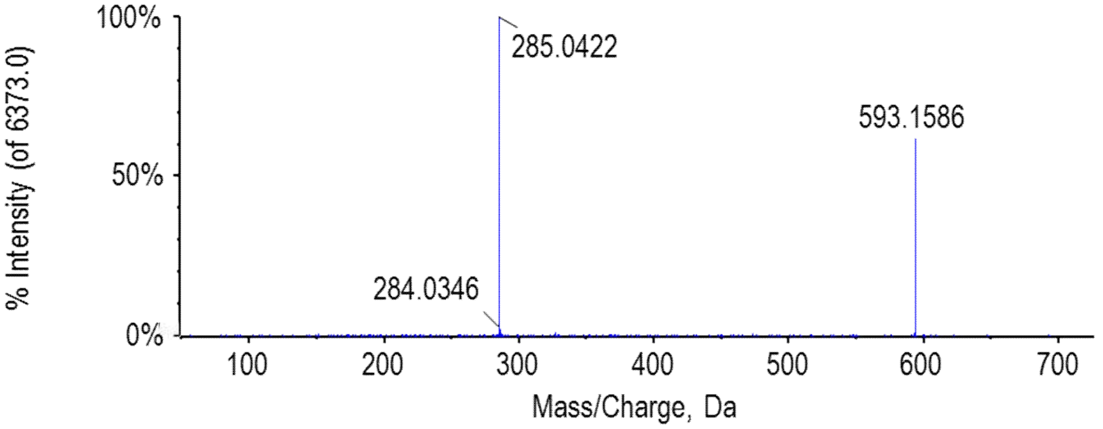

F16. Hesperidin (RT=11.6 min)

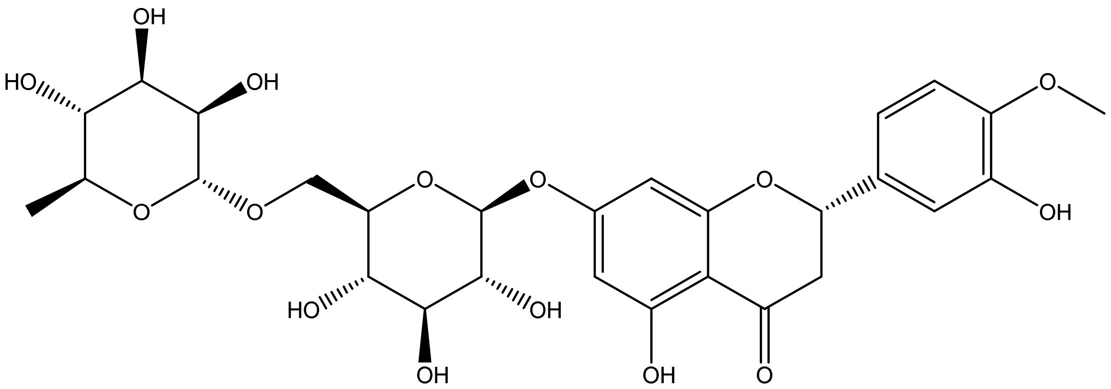

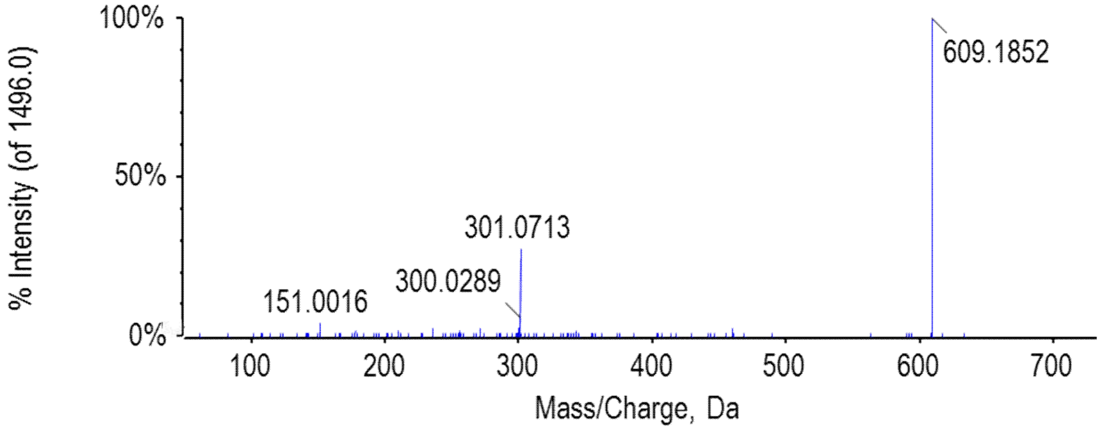

F17. Poncirin (RT=13.9 min)

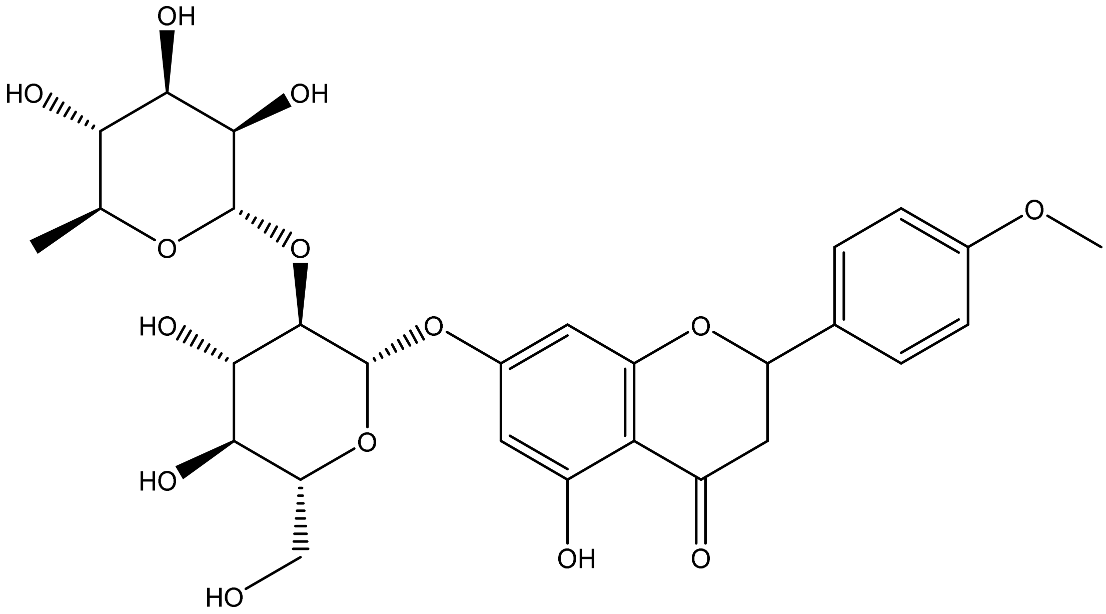

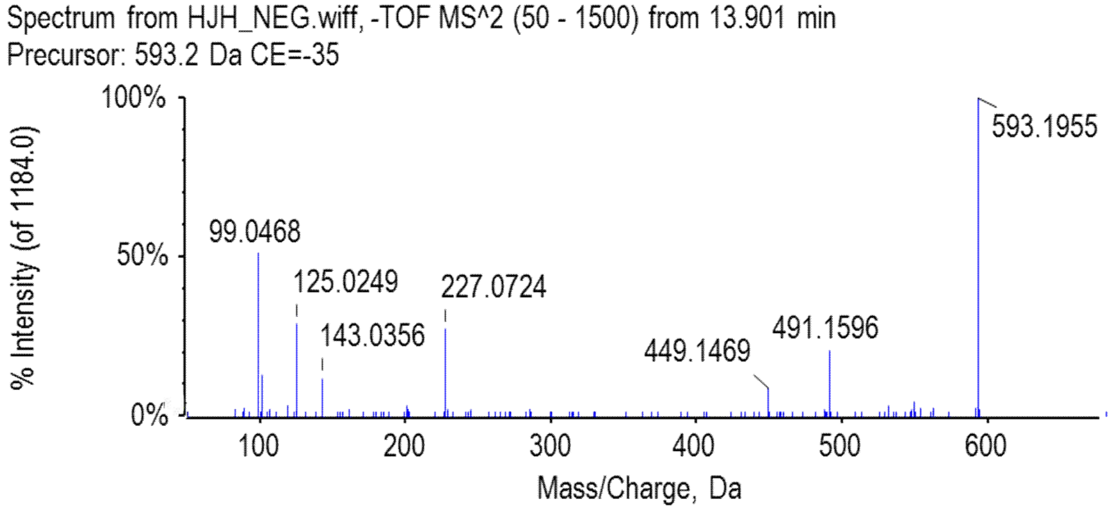

F18. Kaempferol (RT=14.7 min)

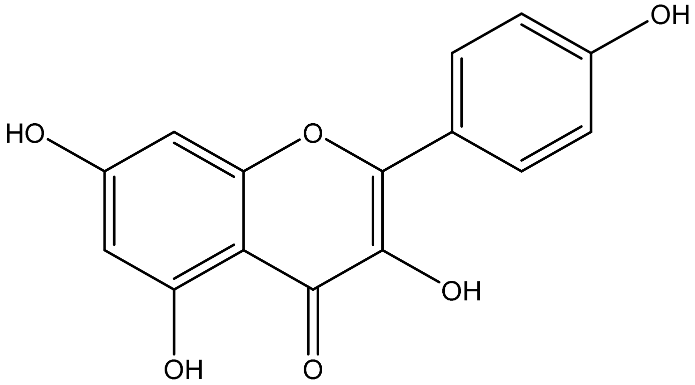

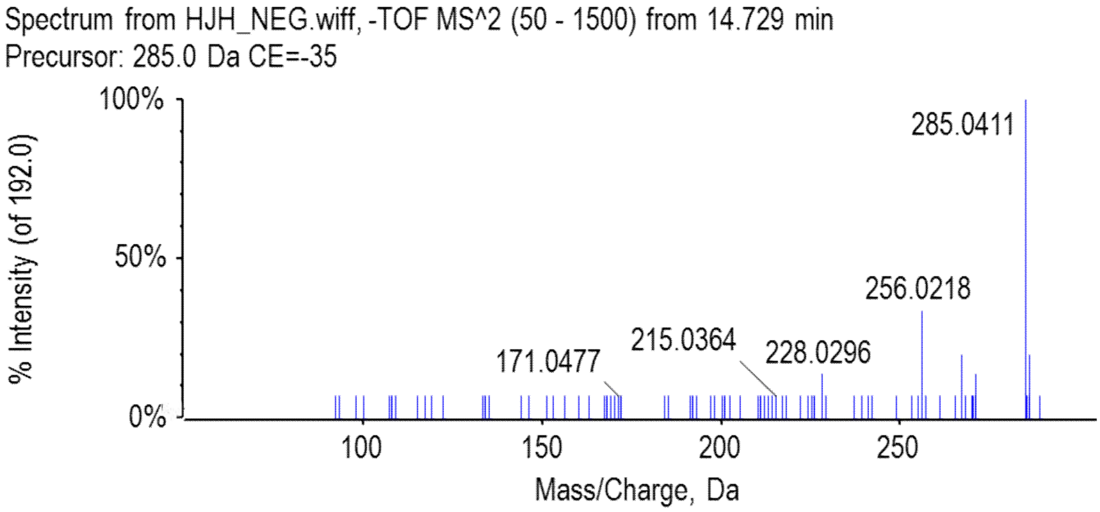

Part II. Structures and product ion spectra of identified metabolites in urine after the consumption of 250 mL Exocarpium *Citri grandis* extract

M1. Naringenin (RT=13.6 min)

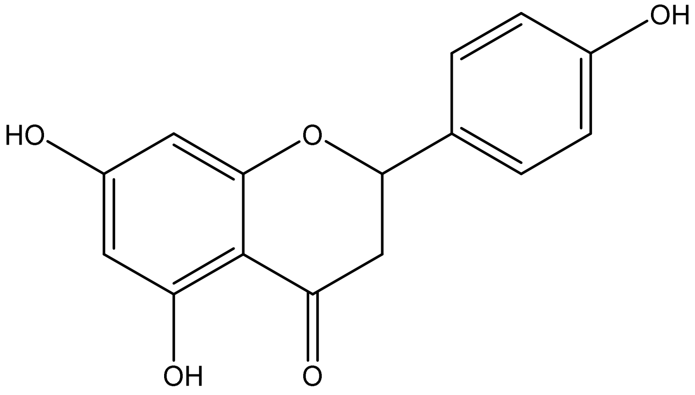

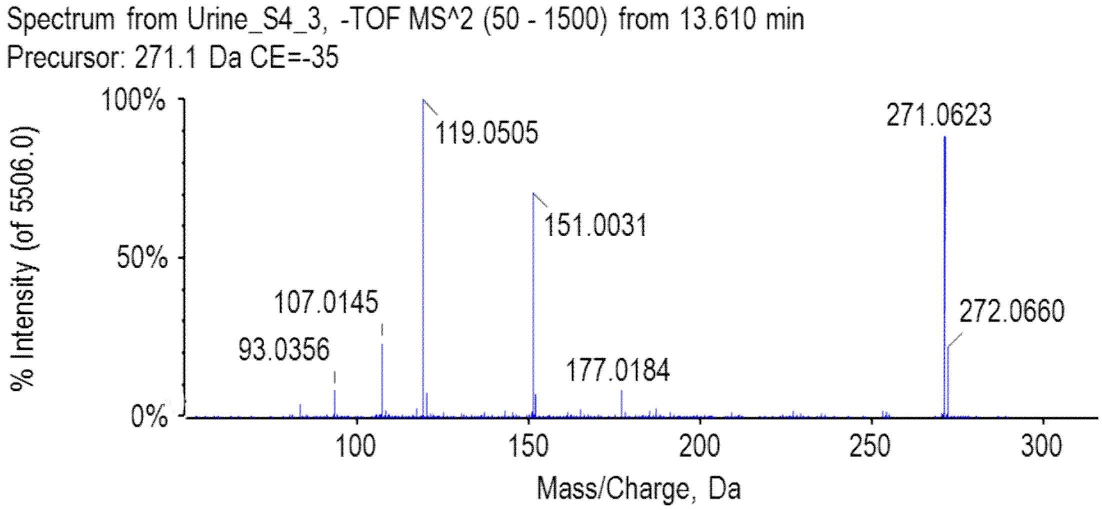

M2. Naringenin-4′,7-O-diglucuronide (RT=8.8 min)

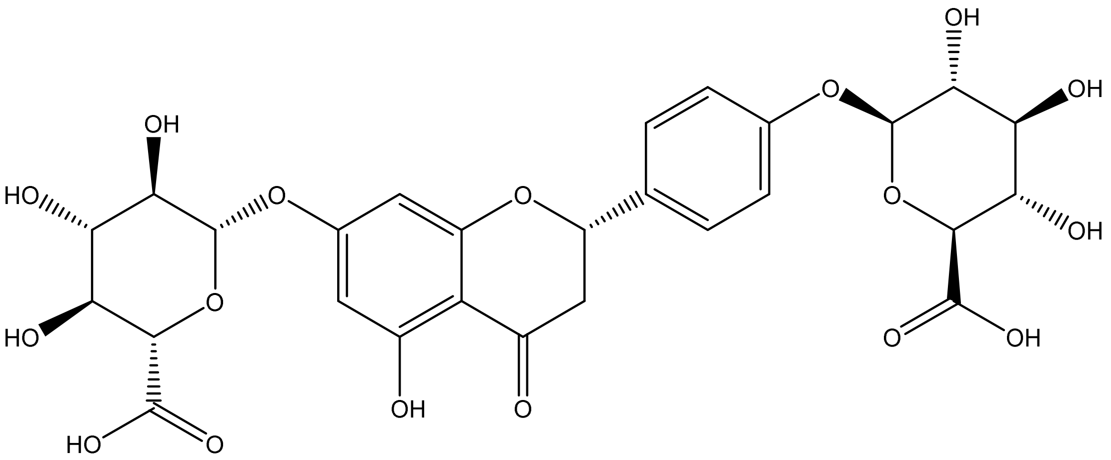

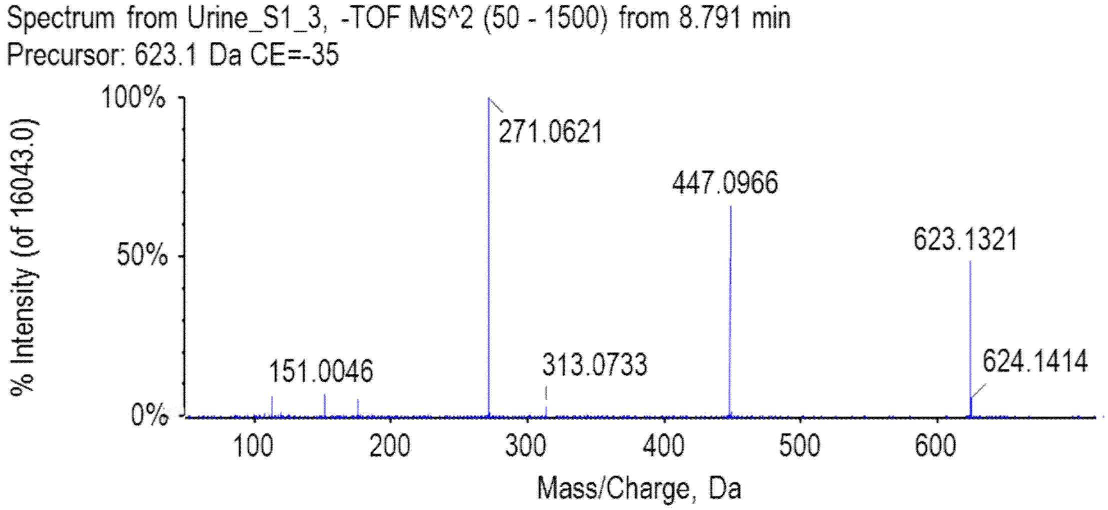

M3. Naringenin-5,7-O-diglucuronide (RT=9.4 min)

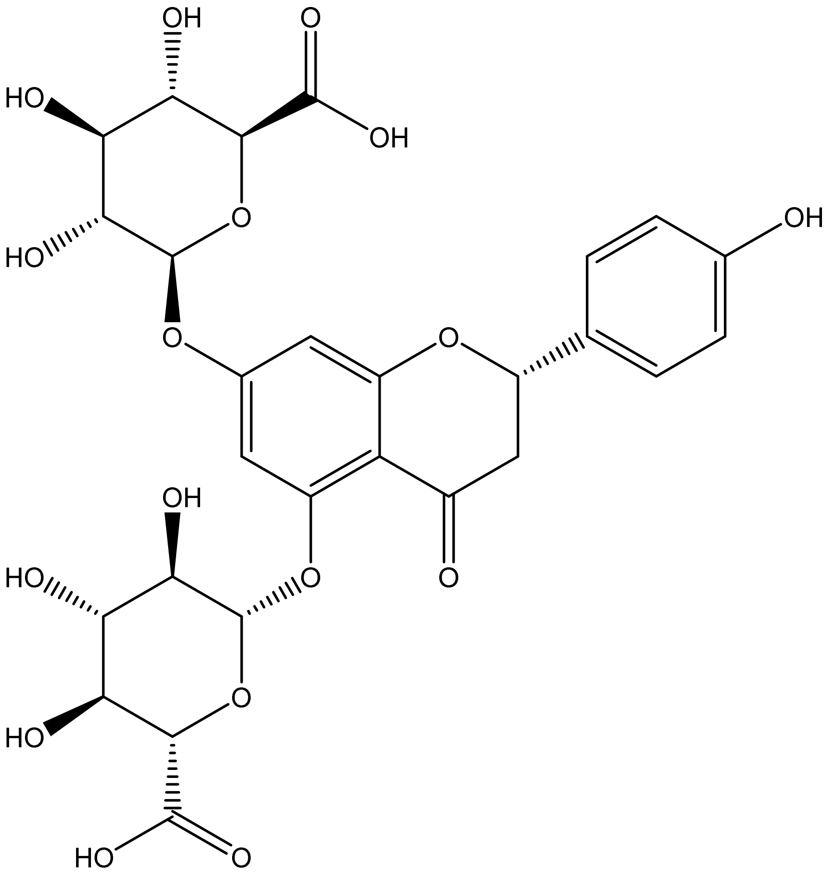

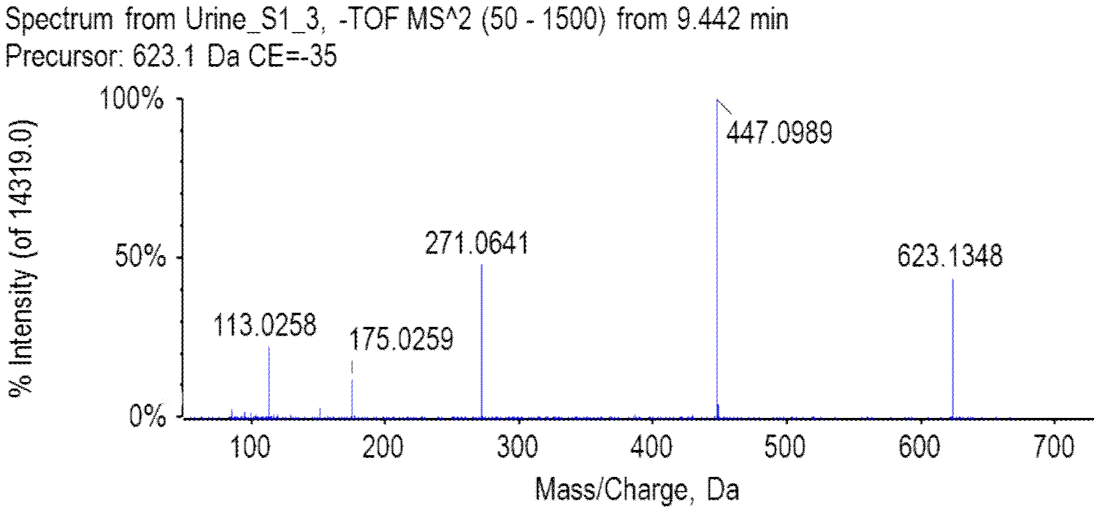

M4. Naringenin-4′,5-O-diglucuronide (RT=10.7 min)

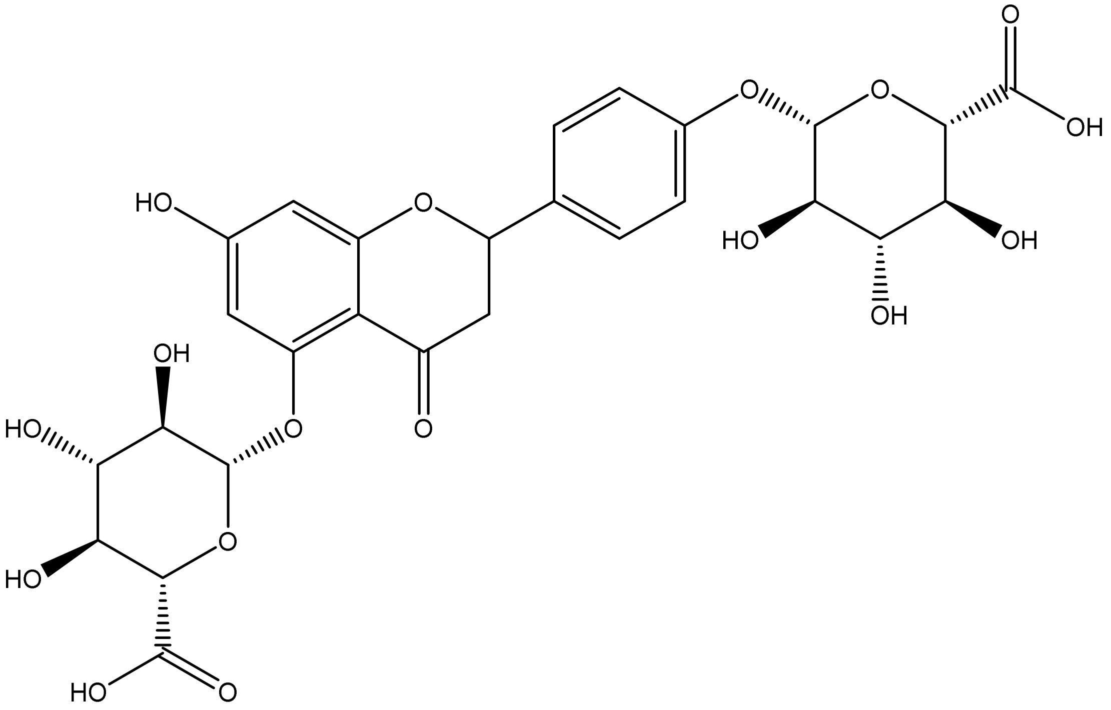

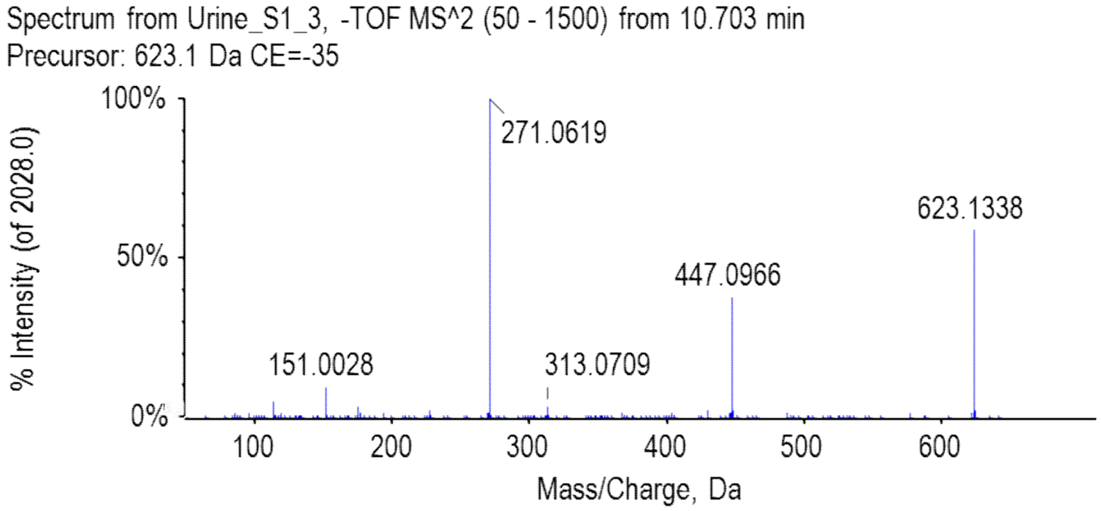

M5. Naringenin-O-glucoside-O-sulfate (RT=9.3 min)

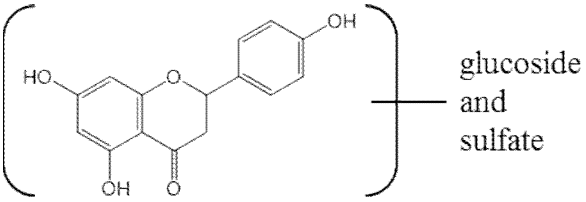

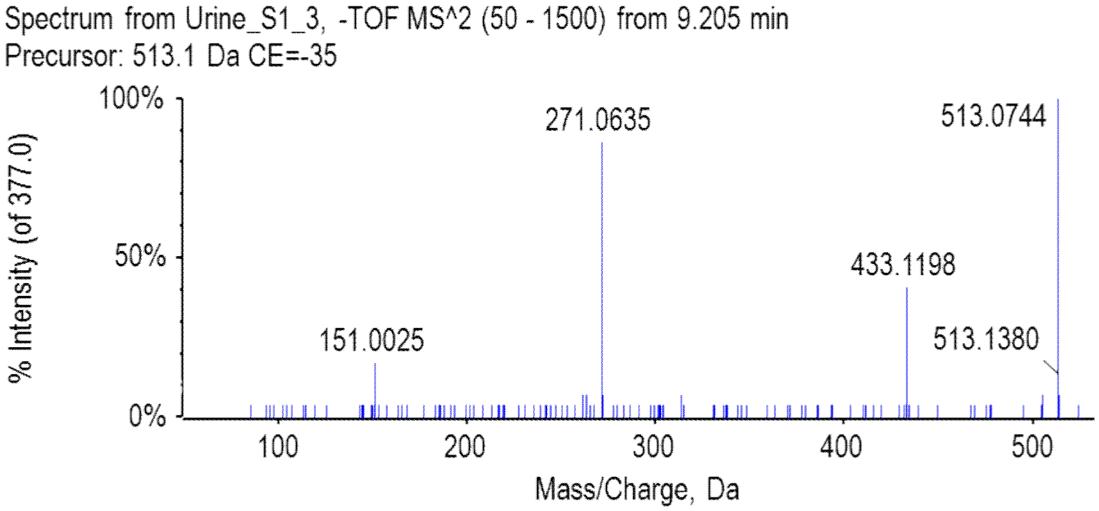

M6. Naringenin-O-glucoside-O-sulfate (RT=9.9 min)

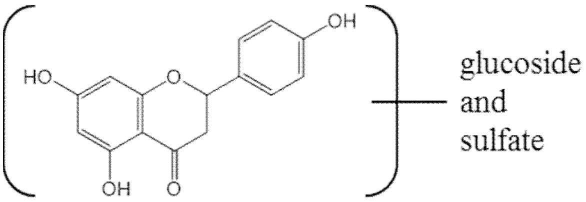

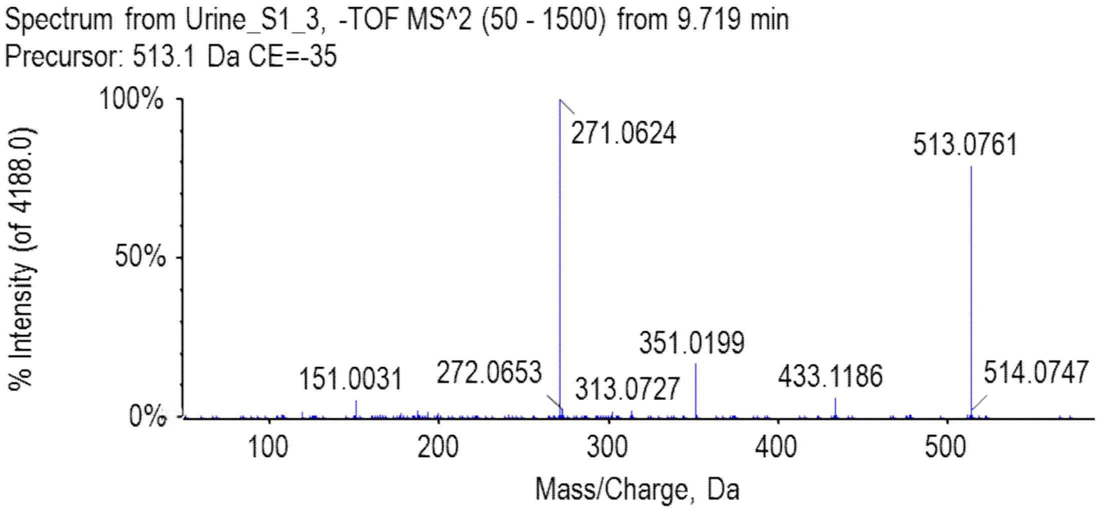

M7. Naringenin-O-glucoside-O-glucuronide (RT=9.4 min)

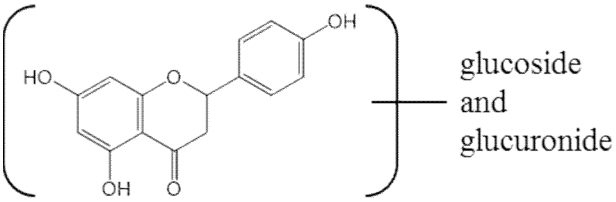

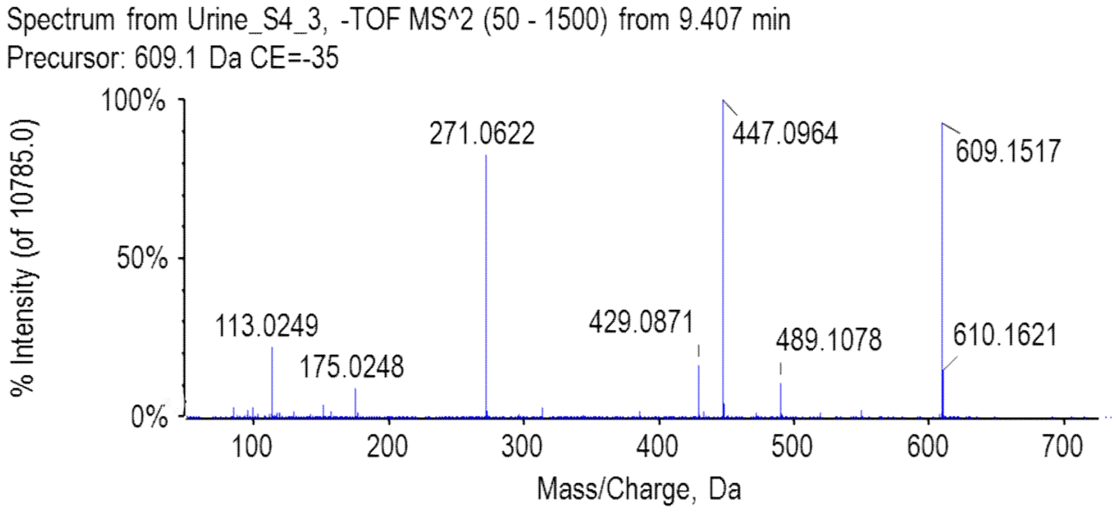

M8. Naringenin-O-glucuronide-O-sulfate (RT=10.1 min)

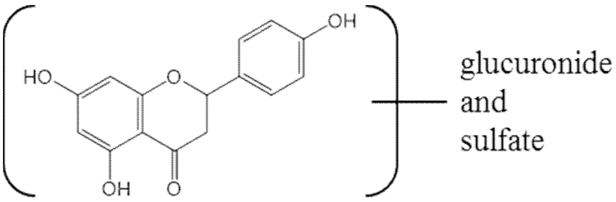

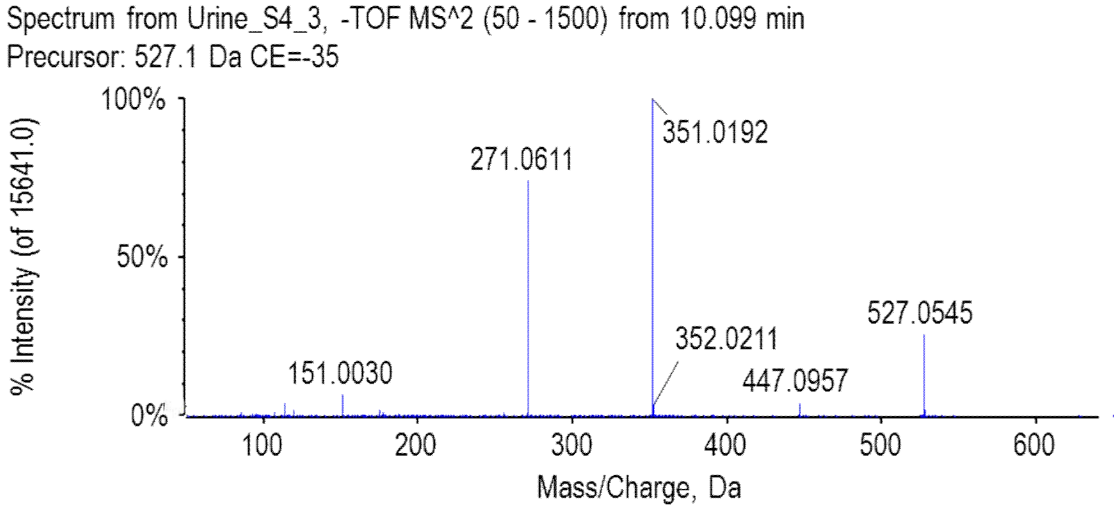

M9. Naringenin-4’-O-sulfate (RT=10.3 min)

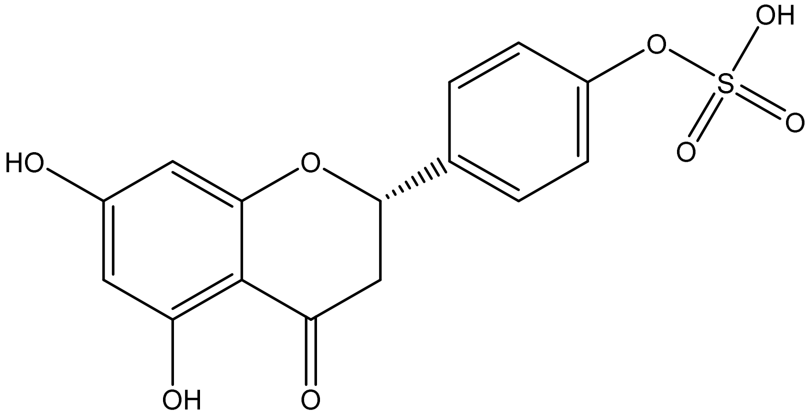

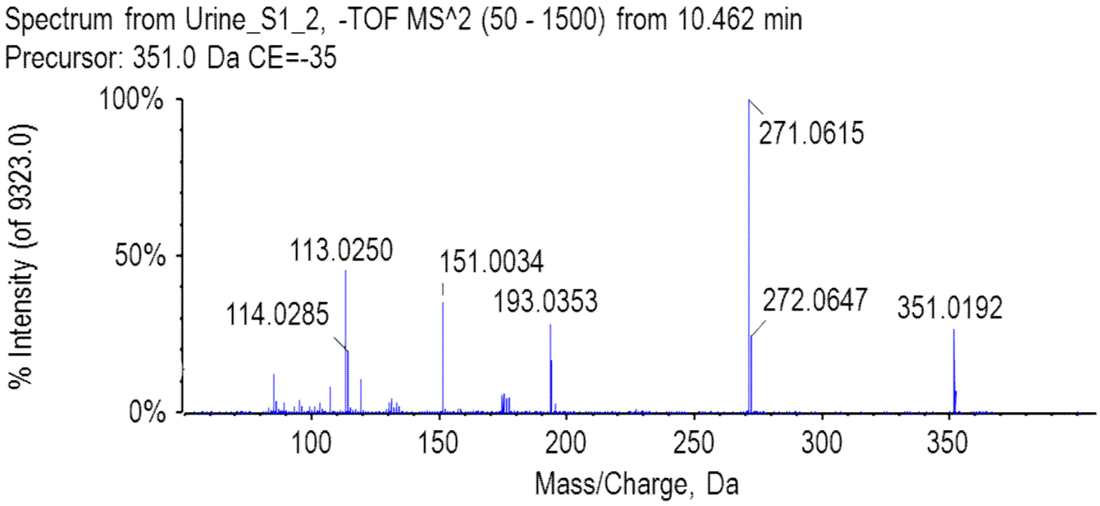

M10. Naringenin-7-O-sulfate (RT=12.3 min)

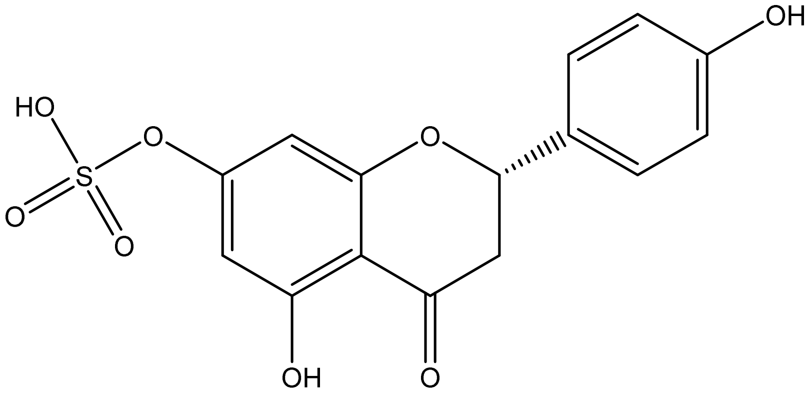

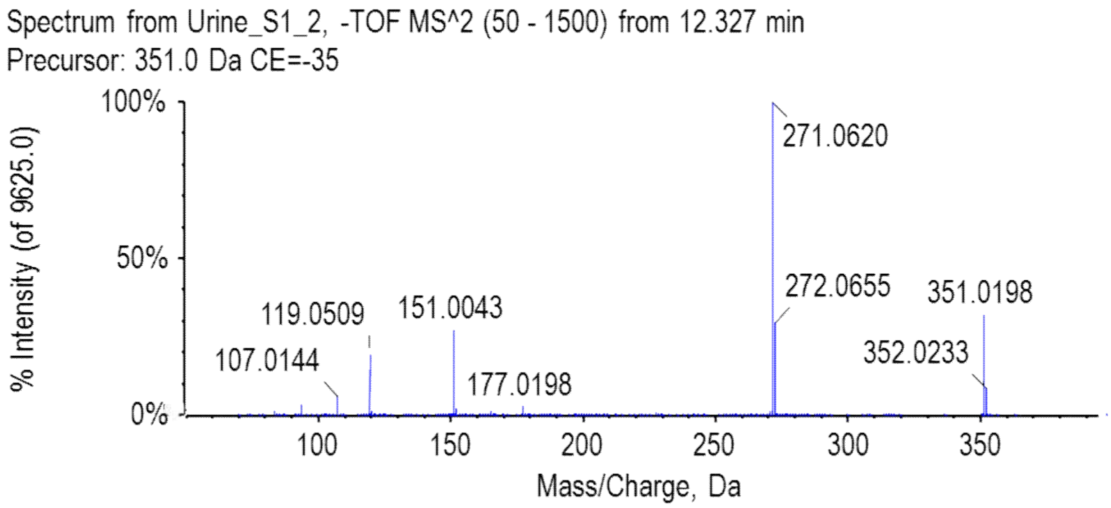

M11. Naringenin-5-O-glucuronide (RT=10.7 min)

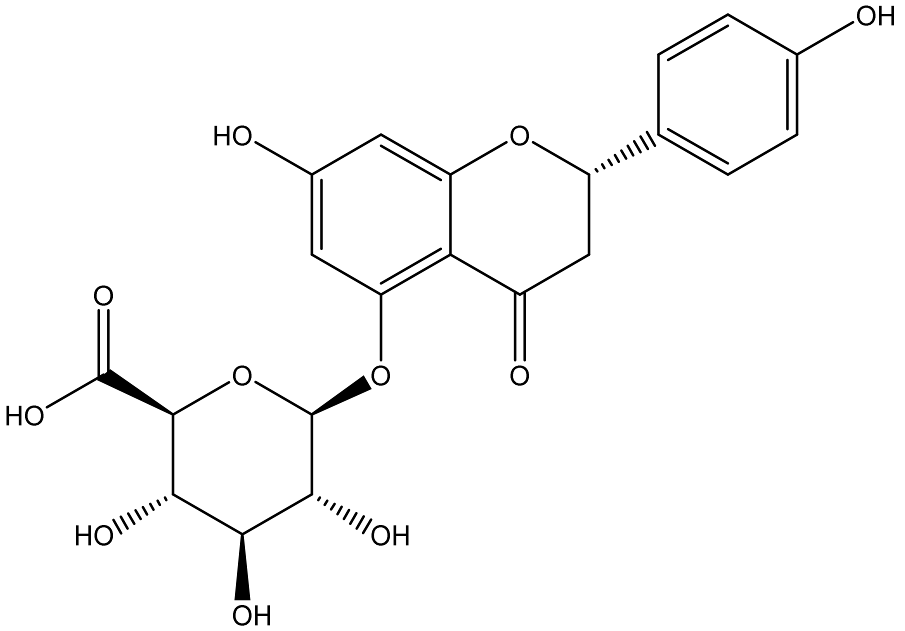

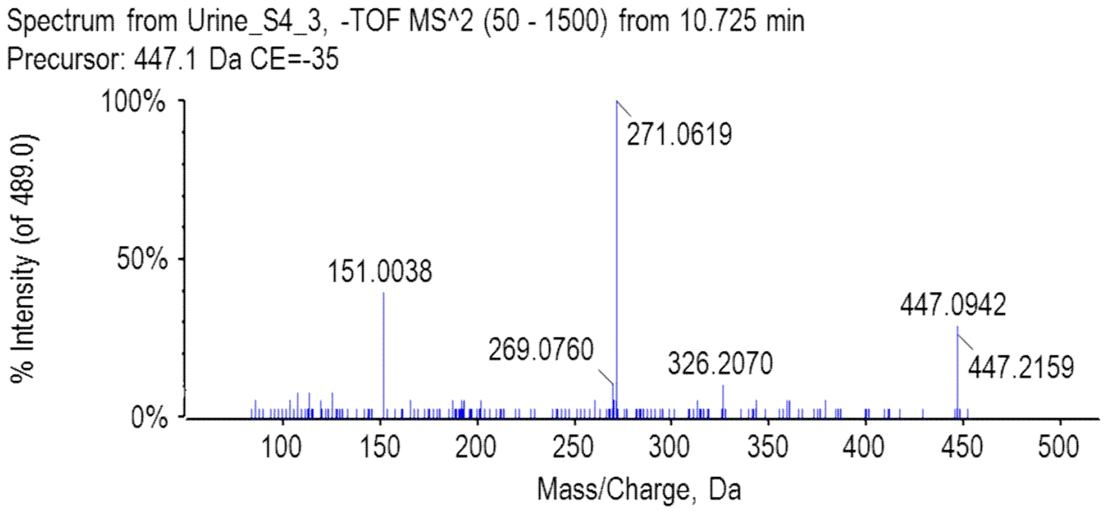

M12. Naringenin-7-O-glucuronide (RT=11.4 min)

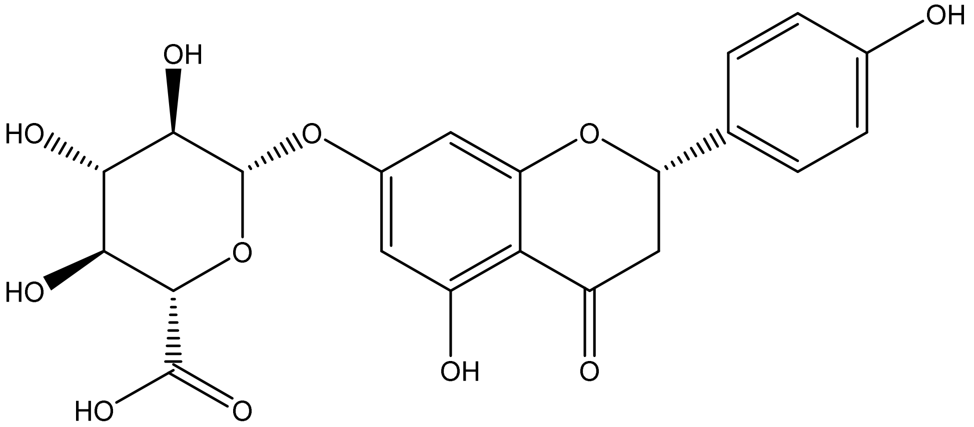

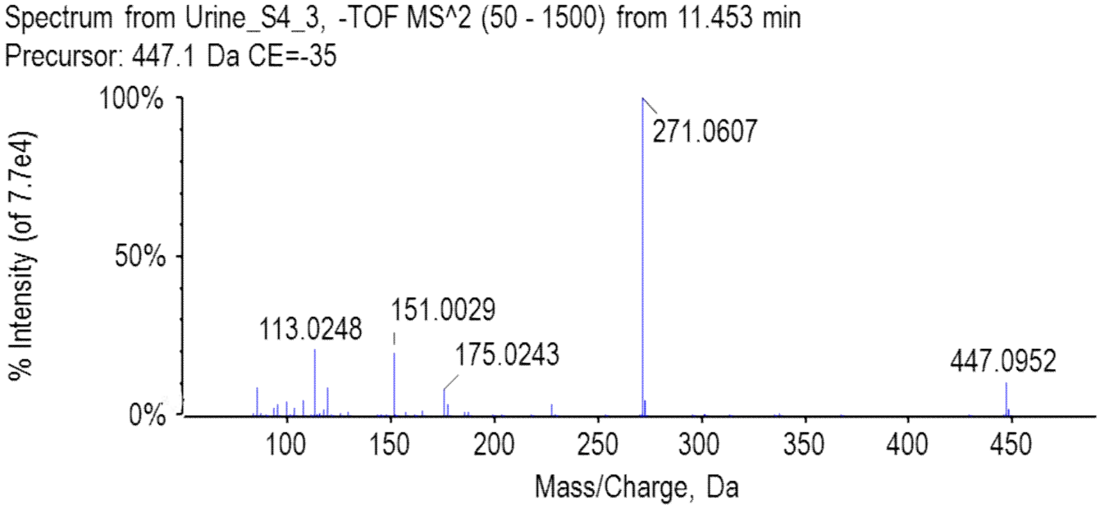

M13. Naringenin-4'-O-glucuronide (RT=11.7 min)

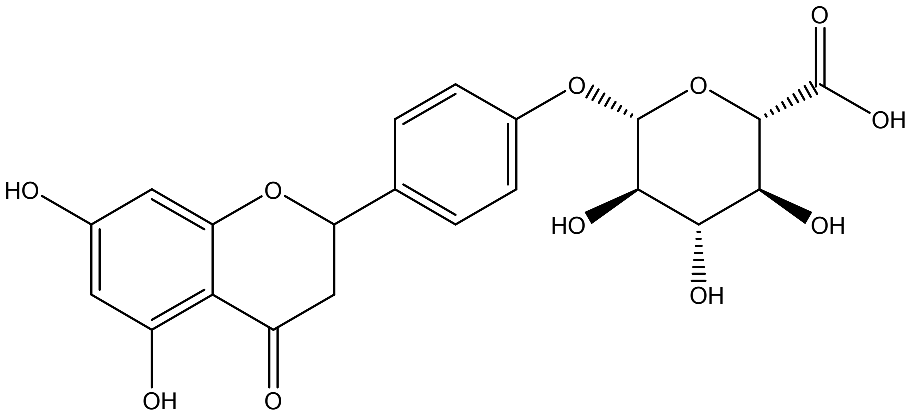

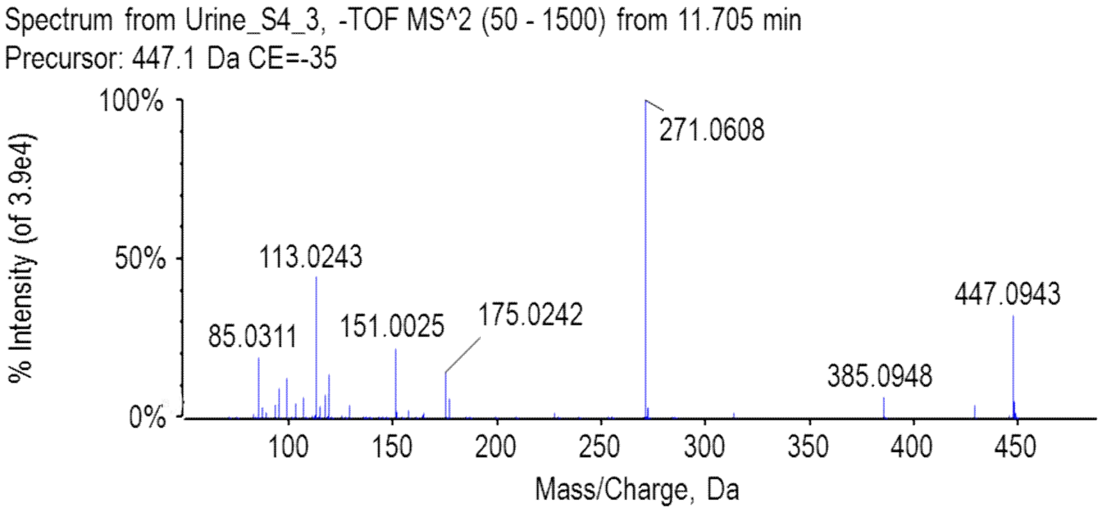

M14. Hesperetin-3'-O-sulfate (RT=10.4 min)

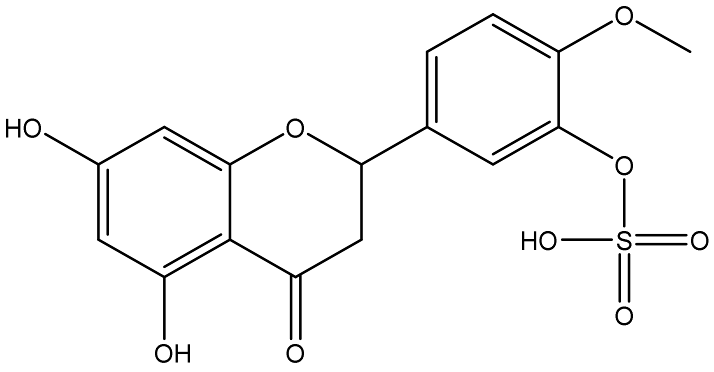

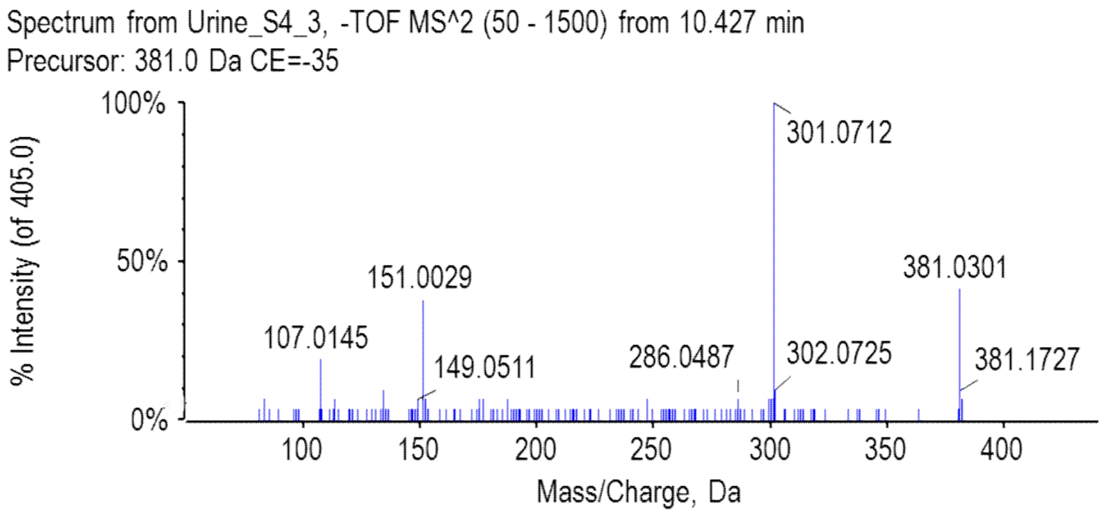

M15. Hesperetin-7-O-sulfate (RT=12.6 min)

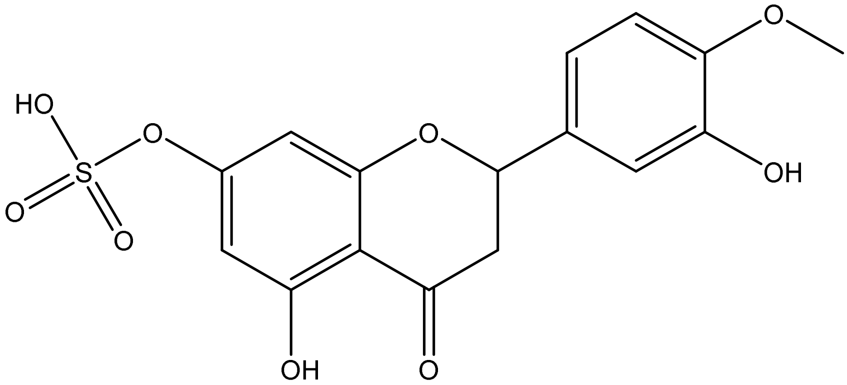

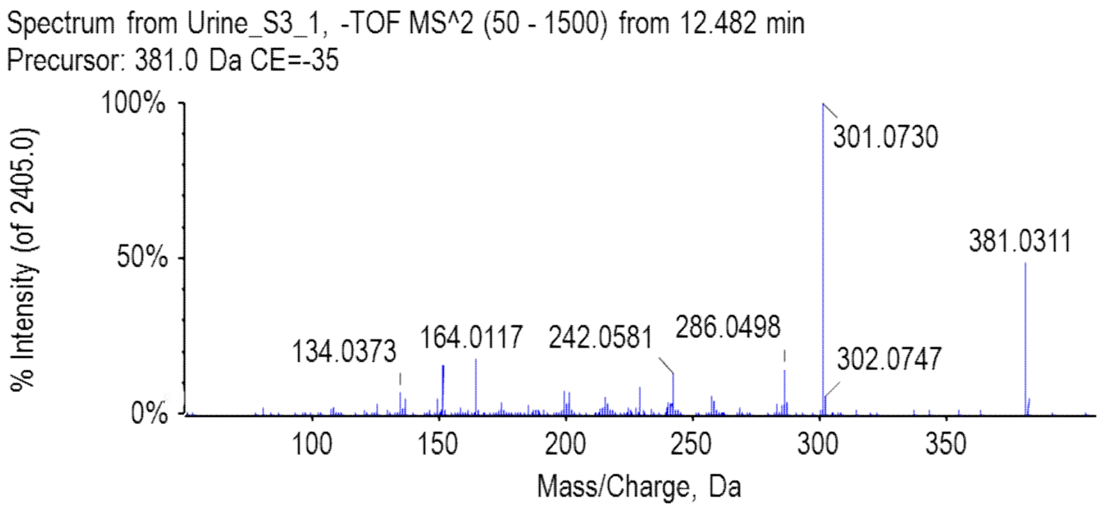

M16. Hesperetin-7-O-glucuronide (RT=11.8 min)

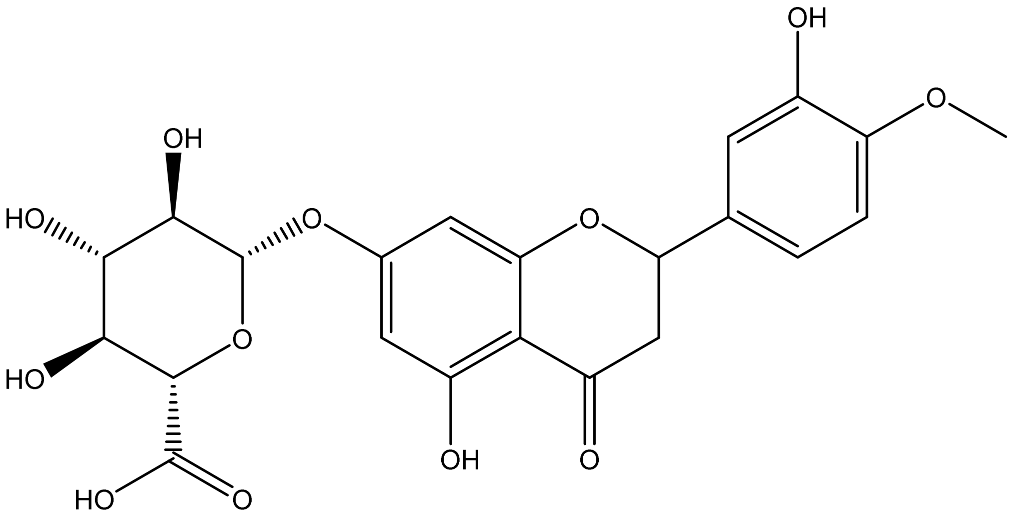

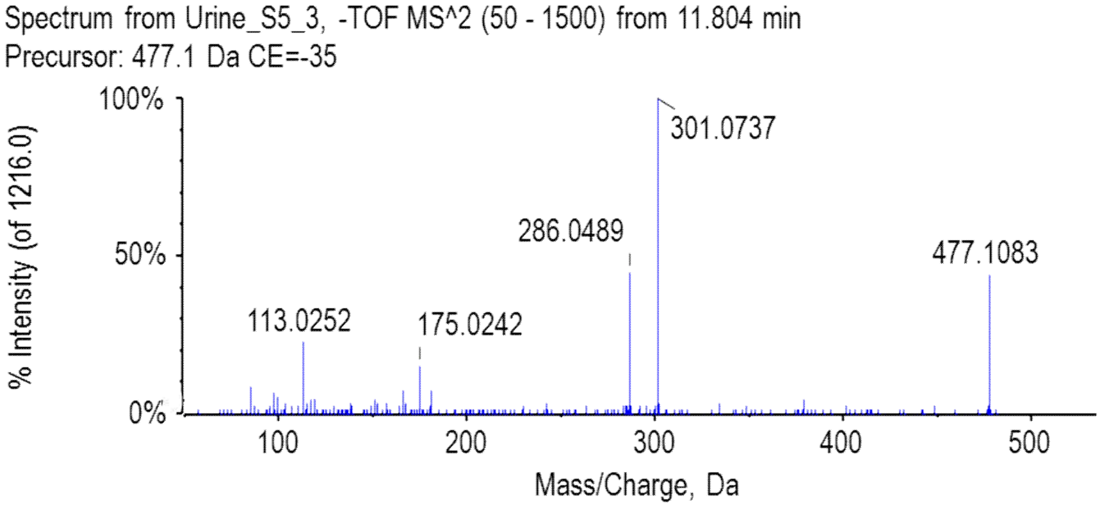

M17. Hesperetin-3'-O-glucuronide (RT=12.3 min)

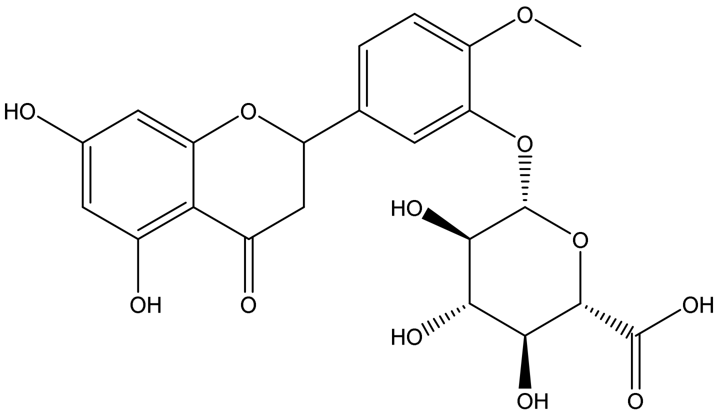

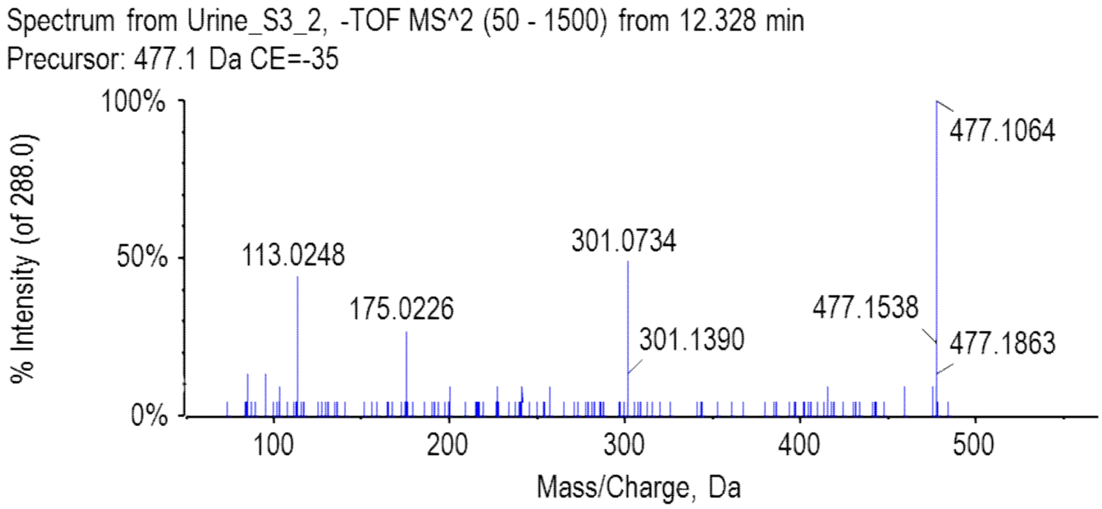

M18. Eriodictyol-O-glucuronide (RT=10.9 min)

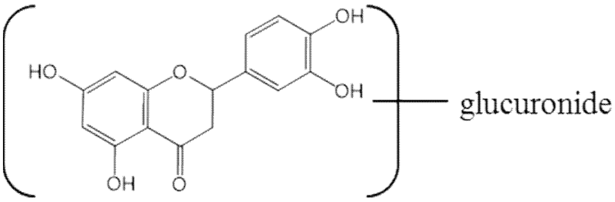

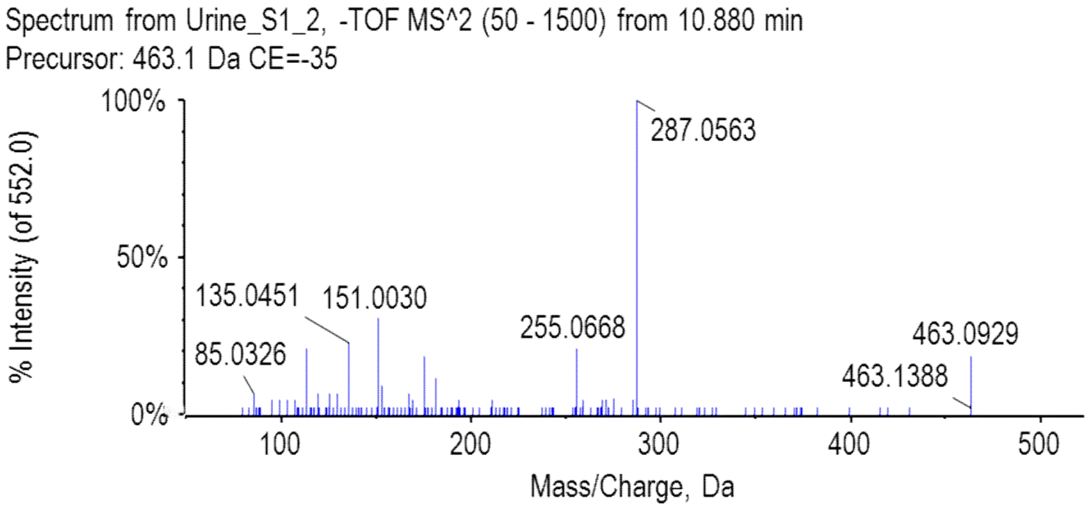

M19. Eriodictyol-O-sulfate (RT=12.5 min)

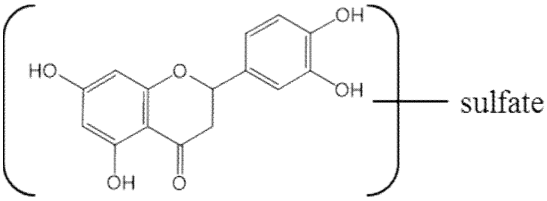

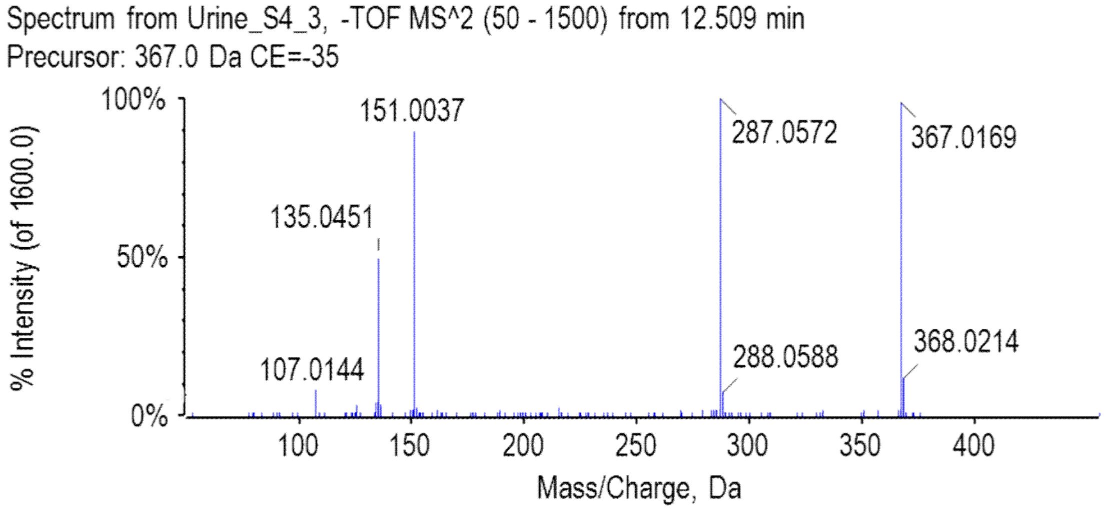

M20. Apigenin-O-glucuronide (RT=12.6 min)

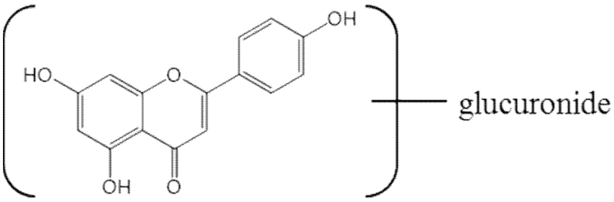

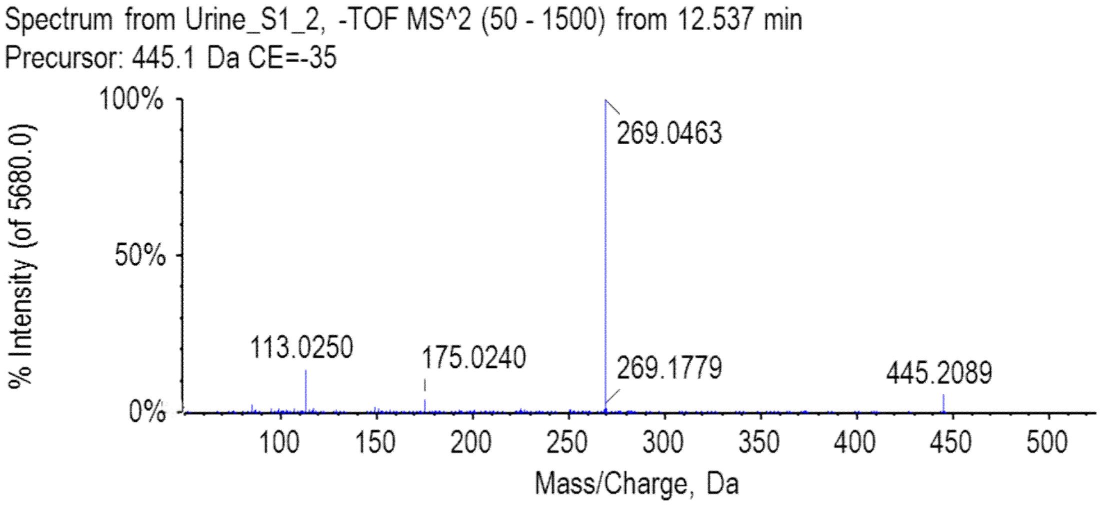

Part III. Detailed information, structures, and product ion spectra of identified phenolic catabolites in urine after the consumption of 250 mL Exocarpium *Citri grandis* extract

Table 1. UFLC-Q-TOF-MS/MS based identifications of phenolic catabolites in human urine collected 0-48 h after 250 mL Exocarpium *Citri grandis* extract consumption.

No.	Identified metabolites	Formula	RT (min)	$[M-H]^-$	Fragment ions in negative (−) ion mode [a]
	Phenylpropanoid acid derivatives				
C1	Ferulic acid or Isoferulic acid [b, c]	$C_{10}H_{10}O_4$	8.7	193.0515	178.0279$[M-H-CH_3]^-$, 149.0617$[M-H-CO_2]^-$, 134.0375$[M-H-CH_3-CO_2]^-$
C2	Ferulic acid-4′-O-glucuronide	$C_{16}H_{18}O_{10}$	8.3	369.0865	193.0502$[M-H-GlcUA]^-$, 178.0269$[M-H-GlcUA-CH_3]^-$, 175.0688$[M-H-FC]^-$, 149.0058$[M-H-GlcUA-CO_2]^-$, 134.0377$[M-H-GlcUA-CH_3-CO_2]^-$, 113.0250$[M-H-FC-CO_2-H_2O]^-$, 85.0308$[M-H-FC-CO_2-H_2O-CO]$-
C3	Isoferulic acid-3′-O-glucuronide	$C_{16}H_{18}O_{10}$	9.5	369.0862	193.0501$[M-H-GlcUA]^-$, 178.0433$[M-H-GlcUA-CH_3]^-$, 175.0622$[M-H-IFC]^-$, 134.0378$[M-H-GlcUA-CH_3-CO_2]^-$, 113.0240$[M-H-IFC-CO_2-H_2O]^-$, 85.0303$[M-H-IFC-CO_2-H_2O-CO]$-
C4	Ferulic acid-4’-sulfate or Isoferulic acid-3’-sulfate	$C_{10}H_{10}O_7S$	8.8	273.0072	193.0503$[M-H-SO_3]^-$, 178.0267$[M-H-SO_3-CH_3]^-$, 149.0605$[M-H-SO_3-CO_2]^-$, 134.0363$[M-H-SO_3-CH_3-CO_2]^-$, 96.9612

C5	Caffeic acid-4′-sulfate or Caffeic acid-3′-sulfate	$C_9H_8O_7S$	8.6	259.1307	241.1221[M-H-H_2O]$^-$, 179.0354[M-H-SO_3]$^-$, 135.0446[M-H-SO_3-CO_2]$^-$
C6	Caffeic acid-4′-O-glucuronide or Caffeic acid-3′-O-glucuronide	$C_{15}H_{16}O_{10}$	9.2	355.1042	179.0709[M-H-GlcUA]$^-$, 175.0244[M-H-CA]$^-$, 135.0422[M-H-GlcUA-CO_2]$^-$, 113.0250[M-H-CA-CO_2-H_2O]$^-$, 85.0293[M-H-CA-CO_2-H_2O-CO]$^-$
C7	Feruloylglycine	$C_{12}H_{13}NO_5$	9.5	250.0723	232.0604[M-H-H_2O]$^-$, 206.0821[M-H-CO_2]$^-$, 191.0778[M-H-CO_2-CH_3]$^-$, 163.0625[M-H-CO_2-CH_3-CO]$^-$, 149.0458[M-H-CO_2-C_2H_3NO]$^-$, 134.0374[M-H-CO_2-C_2H_3NO-CH_3]$^-$, 100.0046
C8	3'-Hydroxycinnamic acid or 4'-Hydroxycinnamic acid	$C_9H_8O_3$	10.7	163.0354	119.0487[M-H-CO_2]$^-$, 93.0362[M-H-CO-C_2H_2]$^-$
	Phenylpropionic acid derivatives				
C9	3-(3’-Hydroxy-4’-methoxyphenyl) hydracrylic acid	$C_{10}H_{12}O_5$	11.6	211.0617	193.0494[M-H-H_2O]$^-$, 167.0708[M-H-CO_2]$^-$, 149.0662[M-H-H_2O-CO_2]$^-$, 123.0809[M-H-H_2O-CO_2-C_2H_2]$^-$, 107.0514[M-H-H_2O-CO_2-C_2H_2-CH_4]$^-$, 95.0495
C10	3-(3′-Methoxyphenyl) propionic	$C_{10}H_{12}O_7S$	8.6	275.0248	195.0664[M-H-SO_3]$^-$, 151.0765[M-H-SO_3-CO_2]$^-$,

	acid-4′-sulfate				136.0530[M-H-SO_3-CO_2-CH_3]$^-$, 108.0488[M-H-SO_3-CO_2-CH_3-C_2H_4]$^-$, 79.9596[M-H-SO_3-CO_2-CH_3-C_2H_4-CO]$^-$
C11	3-(4′-Methoxyphenyl) propionic acid-3′-sulfate	$C_{10}H_{12}O_7S$	9.0	275.0225	233.0966, 195.0656[M-H-SO_3]$^-$, 151.0755[M-H-SO_3-CO_2]$^-$, 149.0605[M-H-SO_3-HCOOH]$^-$, 136.0521[M-H-SO_3-CO_2-CH_3]$^-$, 119.0504, 108.0420[M-H-SO_3-CO_2-CH_3-C_2H_4]$^-$, 79.9593[M-H-SO_3-CO_2-CH_3-C_2H_4-CO]$^-$
C12	3-(3′-Methoxyphenyl) propionic acid-4′-O-glucuronide	$C_{16}H_{20}O_{10}$	8.7	371.1014	353.0888[M-H-H_2O]$^-$, 223.0615, 195.0658[M-H-GlcUA]$^-$, 175.0239[M-H-3MPPA]$^-$, 151.0698[M-H-GlcUA-CO_2]$^-$, 136.0465[M-H-GlcUA-CO_2-CH_3]$^-$, 135.0429[M-H-GlcUA-CO_2-CH_4]$^-$, 113.0242[M-H-3MPPA-CO_2-H_2O]$^-$, 85.0309[M-H-3MPPA-CO_2-H_2O-CO]$^-$
C13	3-(4′-Methoxyphenyl) propionic acid-3′-O-glucuronide	$C_{16}H_{20}O_{10}$	9.4	371.0998	353.0871[M-H-H_2O]$^-$, 195.0665[M-H-GlcUA]$^-$, 175.0247[M-H-4MPPA]$^-$, 151.0744[M-H-GlcUA-CO_2]$^-$, 136.0532[M-H-GlcUA-CO_2-CH_3]$^-$, 113.0251[M-H-4MPPA-CO_2-H_2O]$^-$,

					85.0312[M-H-4MPPA-CO_2-H_2O-CO]$^-$
C14	3-(4'-Hydroxyphenyl) propionic acid or 3-(3'-Hydroxyphenyl) propionic acid [c]	$C_9H_{10}O_3$	11.5	165.0522	137.0217[M-H-CO]$^-$, 93.0345[M-H-CO-C_2H_4O]$^-$
C15	3-(3′,4′-Dihydroxyphenyl) propionic acid	$C_9H_{10}O_4$	8.0	181.0467	163.0383[M-H-H_2O]$^-$, 135.0438[M-H-HCOOH]$^-$, 121.0288[M-H-HCOOH-CH_2]$^-$, 93.0346
C16	3-(3′-Hydroxyphenyl) propionic acid-4’-sulfate	$C_9H_{10}O_7S$	7.3	261.0080	217.0176[M-H-CO_2]$^-$, 181.0502[M-H-SO_3]$^-$, 137.0608[M-H-SO_3-CO_2]$^-$, 122.0374[M-H-SO_3-CO_2-CH_2]$^-$, 121.0299[M-H-SO_3-CO_2-CH_3]$^-$, 79.9594
C17	3-(4′-Hydroxyphenyl) propionic acid-3’-sulfate	$C_9H_{10}O_7S$	8.1	261.0080	181.0510[M-H-SO_3]$^-$, 137.0611[M-H-SO_3-CO_2]$^-$, 121.0300[M-H-SO_3-CO_2-CH_3]$^-$, 109.0255[M-H-SO_3-CO_2-C_2H_4]$^-$, 79.9591
C18	3-(Phenyl)-2-propenoic acid-4’-sulfate	$C_9H_8O_6S$	8.6	242.9963	163.0393[M-H-SO_3]$^-$, 119.0504[M-H-SO_3-CO_2]$^-$
C19	3-(Phenyl) propionic acid-4′-O-glucuronide	$C_{15}H_{18}O_9$	9.0	341.0890	323.0805[M-H-H_2O]$^-$, 227.1384, 175.0242[M-H-3PPA]$^-$, 165.0554[M-H-GlcUA]$^-$, 121.0661[M-H-GlcUA-CO_2]$^-$, 113.0234[M-H-3PPA-CO_2-H_2O]$^-$, 85.0312[M-H-3PPA-CO_2-H_2O-CO]$^-$
C20	3-(Phenyl) propionic	$C_{15}H_{18}O_9$	10.9	341.0920	175.0252[M-H-3PPA]$^-$, 165.0567[M-H-GlcUA]$^-$,

	acid-3′-O-glucuronide				137.0230[M-H-GlcUA-CO]$^-$, 113.0249[M-H-3PPA-CO_2-H_2O]$^-$, 92.0285[M-H-GlcUA-CO C_2H_5O]$^-$, 85.0322[M-H-3PPA-CO_2-H_2O-CO]$^-$
	Benzoic acid derivatives				
C21	Benzoic acid-4-sulfate or Benzoic acid-3-sulfate	$C_7H_6O_6S$	6.6	216.9816	137.0243[M-H-SO_3]$^-$, 93.0360[M-H-SO_3-CO_2]$^-$
C22	3-Hydroxybenzoic acid-4-sulfate	$C_7H_6O_7S$	6.7	233.0112	153.0201[M-H-SO_3]$^-$, 123.0544, 109.0307[M-H-SO_3-CO_2]$^-$
C23	4-Hydroxybenzoic acid-3-sulfate	$C_7H_6O_7S$	7.2	232.9940	153.0195[M-H-SO_3]$^-$, 109.0302[M-H-SO_3-CO_2]$^-$
	Phenylacetic Acid Derivatives				
C24	Hydroxyphenylacetic acid-4′-sulfate or Hydroxyphenylacetic acid-3′-sulfate	$C_8H_8O_6S$	7.0	230.9968	151.0395[M-H-SO_3]$^-$, 107.0506[M-H-SO_3-CO_2]$^-$
C25	3'-Methoxy-4'-hydroxyphenylacetic acid	$C_9H_{10}O_4$	9.5	181.0501	137.0600[M-H-CO_2]$^-$, 122.0375[M-H-CO_2-CH_3]$^-$, 94.0423[M-H-CO_2-CH_3-CO]$^-$
	Hydroxycarboxylic acid derivatives				

C26	3′-Methoxy-4′-hydroxymandelic acid	$C_9H_{10}O_5$	5.8	197.0443	137.0244[M-H-HCOOH-CH_2]$^-$, 108.0211[M-H-HCOOH-CH_2-CHO]$^-$
	Benzoylglycine Derivatives				
C27	Hippuric acid [b, c]	$C_9H_9NO_3$	8.9	178.0511	134.0613[M-H-CO_2]$^-$, 102.0353[M-H-C_6H_4]$^-$, 77.0426[M-H-$C_3H_3NO_3$]$^-$
C28	4’-Hydroxyhippuric acid	$C_9H_9NO_4$	7.3	194.0418	150.0531[M-H-CO_2]$^-$, 121.0297[M-H-CO_2-CH_3N]$^-$, 93.0348[M-H-CO_2-CH_3N-CO]$^-$
C29	3’-Hydroxyhippuric acid	$C_9H_9NO_4$	10.3	194.0434	150.0543[M-H-CO_2]$^-$, 121.0290[M-H-CO_2-CH_3N]$^-$, 93.0356[M-H-CO_2-CH_3N-CO]$^-$

[a] The losses are: FC=Ferulic acid; IFC= isoferulic acid; 3MPPA=3-(3′-methoxyphenyl) propionic acid; 4MPPA=3-(4′-methoxyphenyl) propionic acid; 3PPA=3-(phenyl) propionic acid, CA=caffeic acid.

[b] Confirmation in comparison with mass spectral library (Natural Products HR-MS/MS Spectral Library, Version 1.0, AB Sciex).

[c] Confirmation in comparison with authentic standards.

Structures, and product ion spectra of identified phenolic catabolites

C1. Ferulic acid or Isoferulic acid (RT=8.7 min)

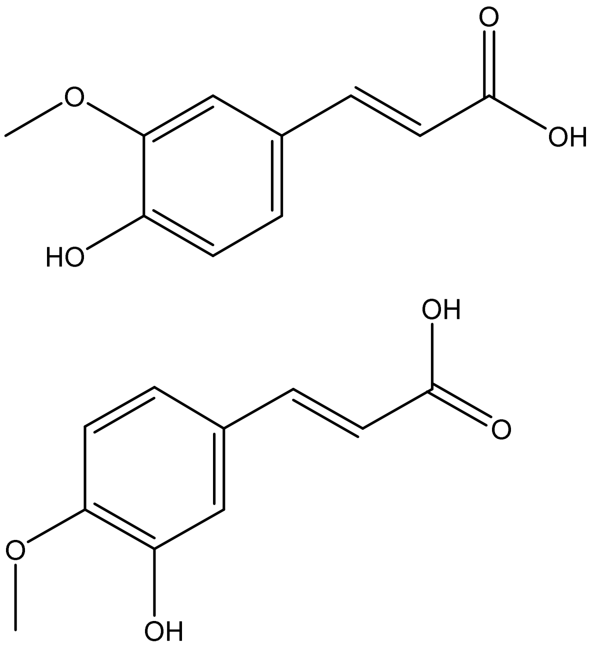

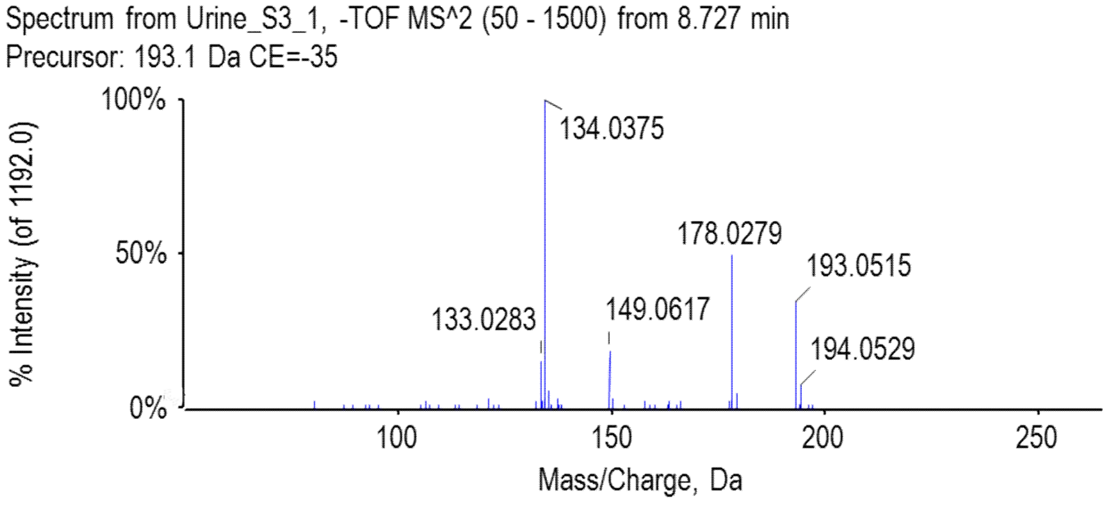

C2. Ferulic acid-4′-O-glucuronide (RT=8.3 min)

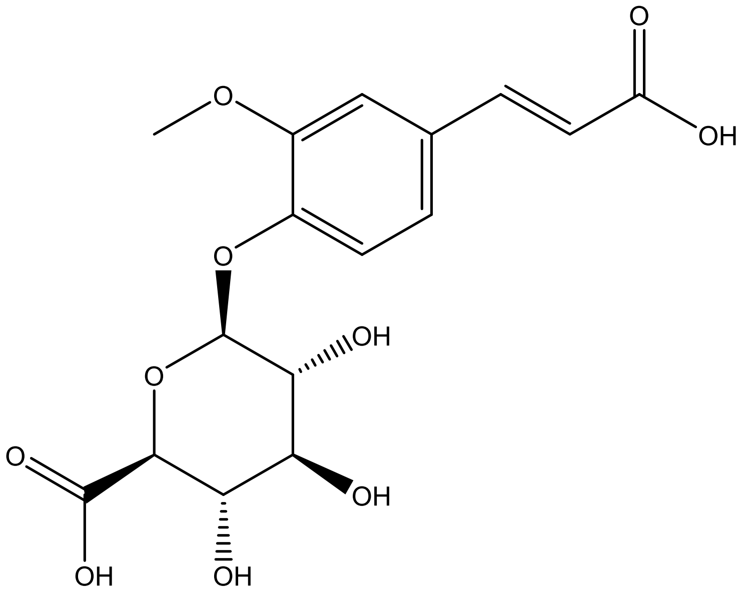

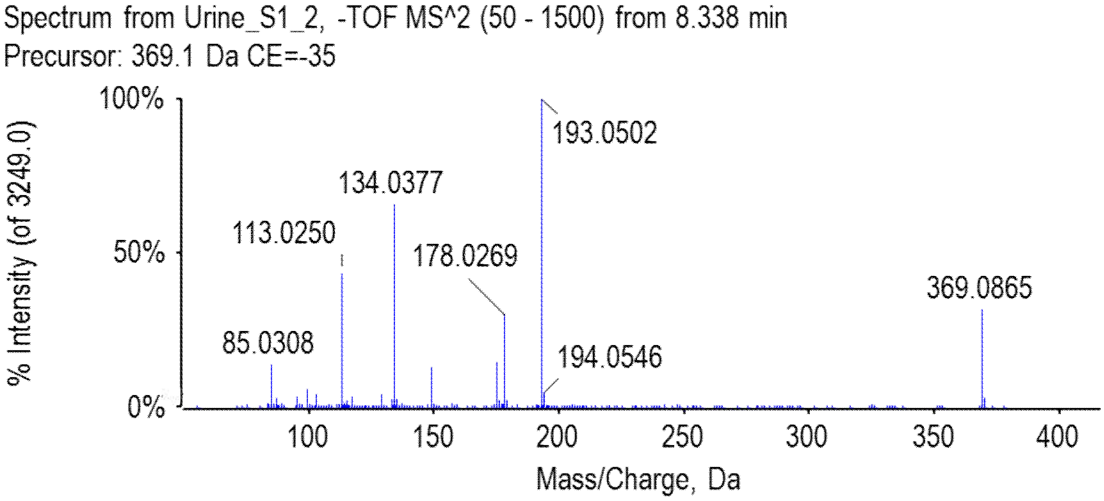

C3. Isoferulic acid-3′-O-glucuronide (RT=9.5 min)

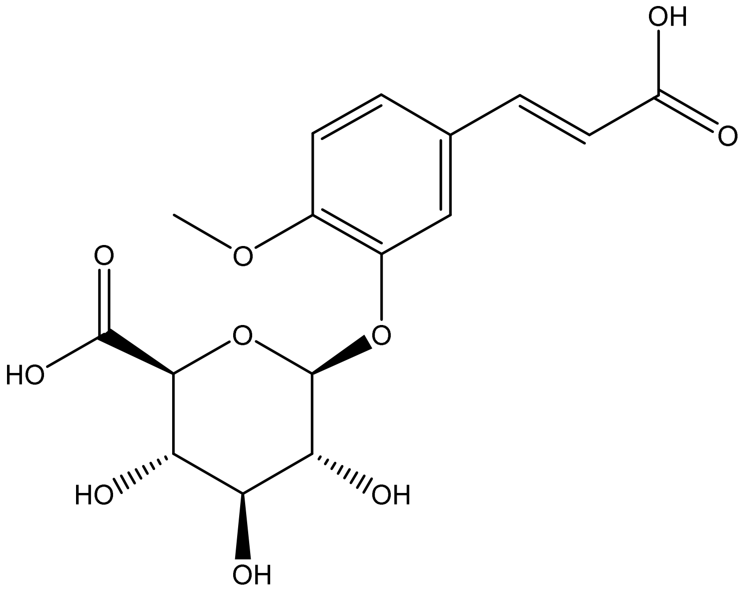

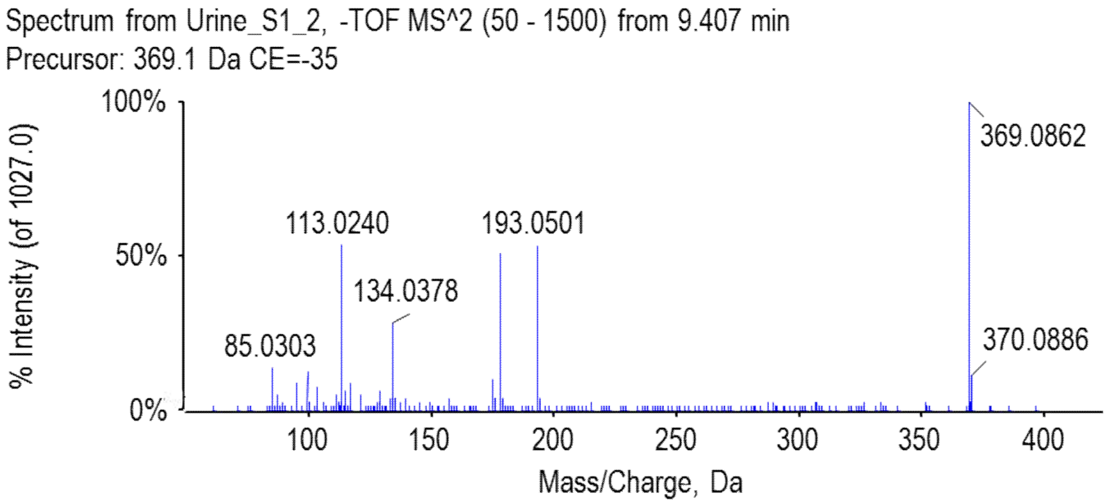

C4. Ferulic acid-4'-sulfate or Isoferulic acid-3'-sulfate (RT=8.8 min)

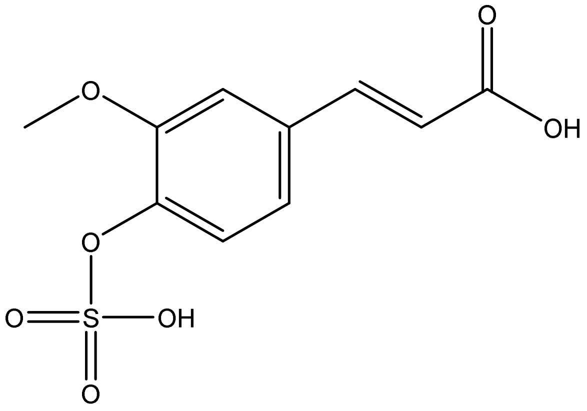

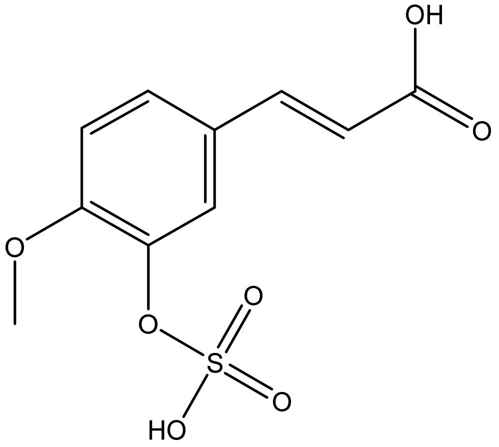

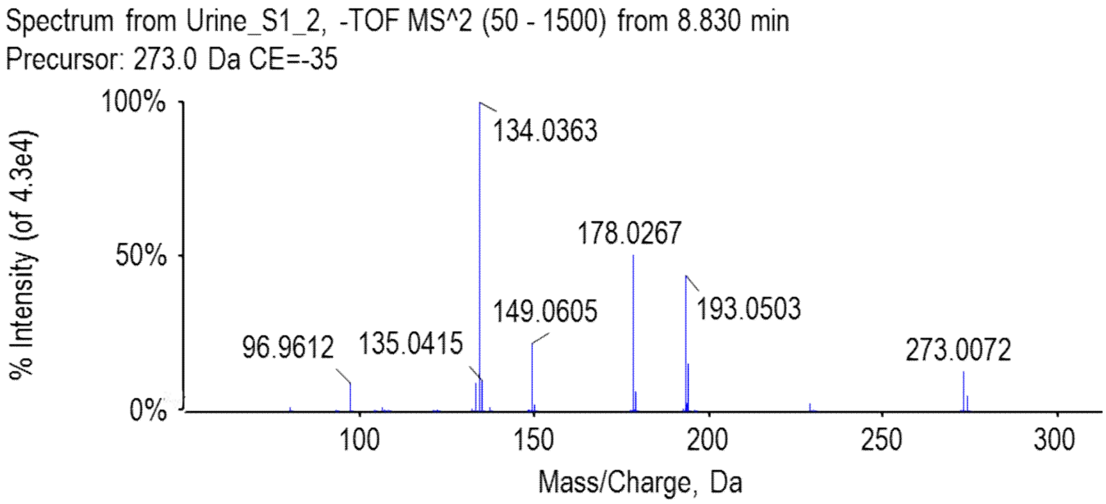

C5. Caffeic acid-4′-sulfate or Caffeic acid-3′-sulfate (RT=8.6 min)

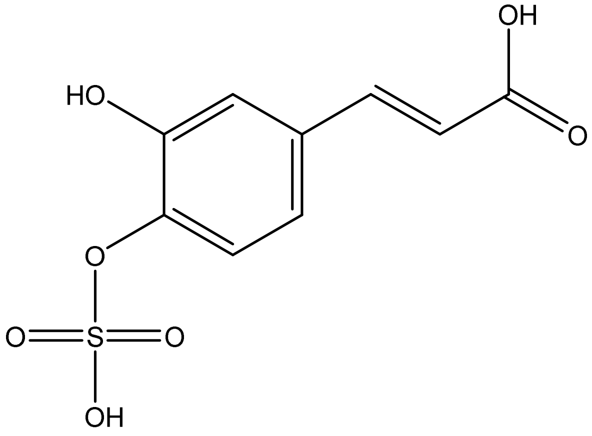

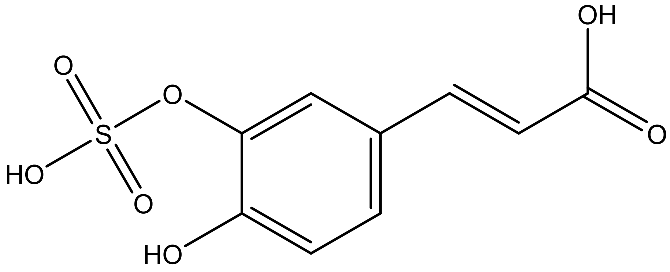

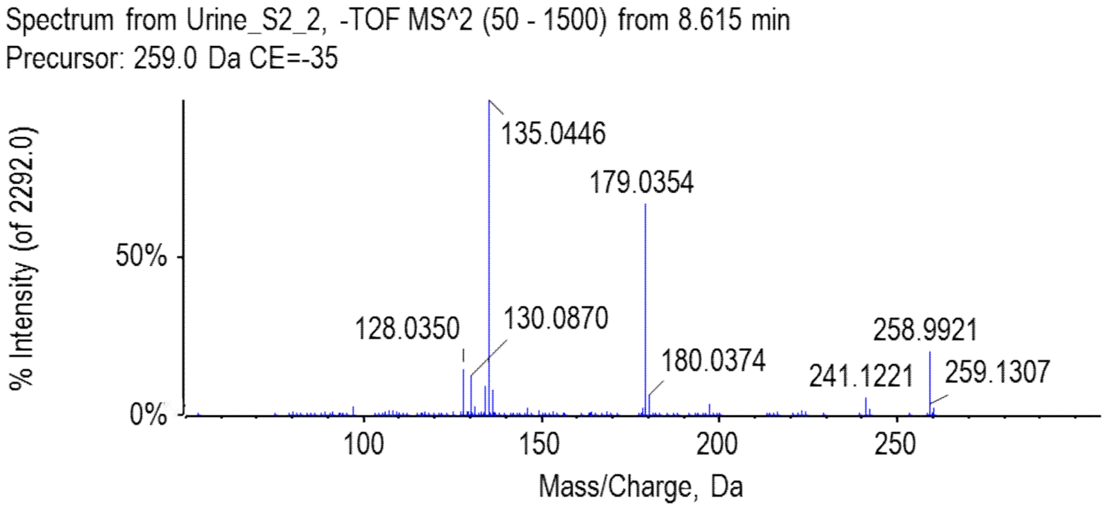

C6. Caffeic acid-4′-O-glucuronide or Caffeic acid-3′-O-glucuronide (RT=9.2 min)

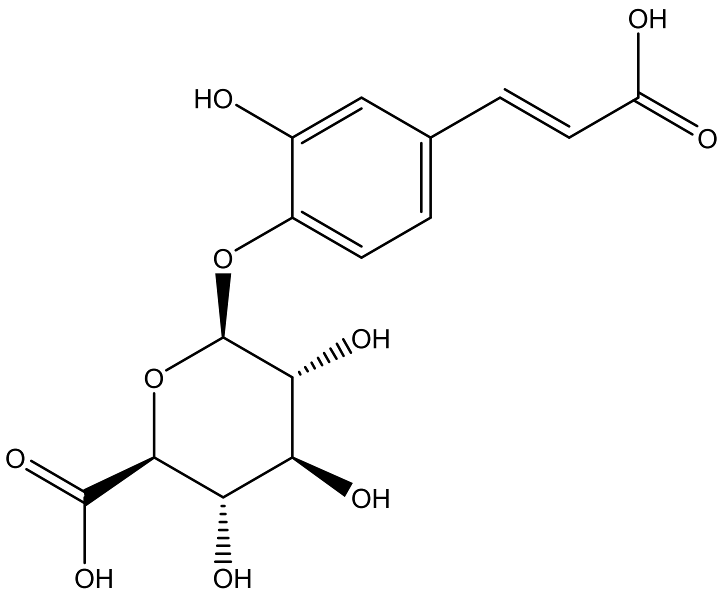

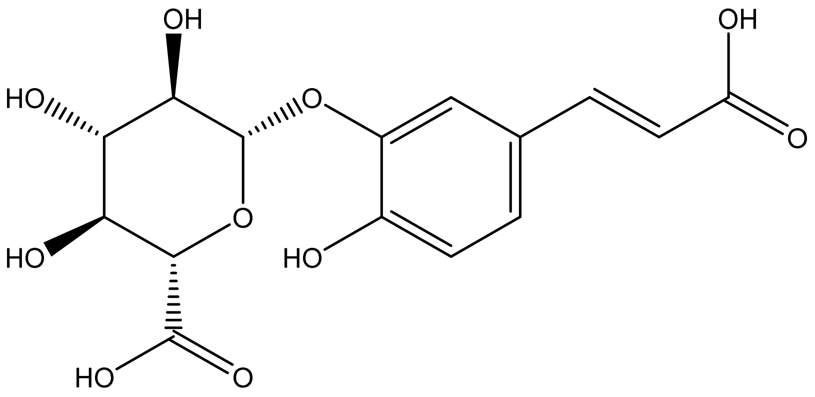

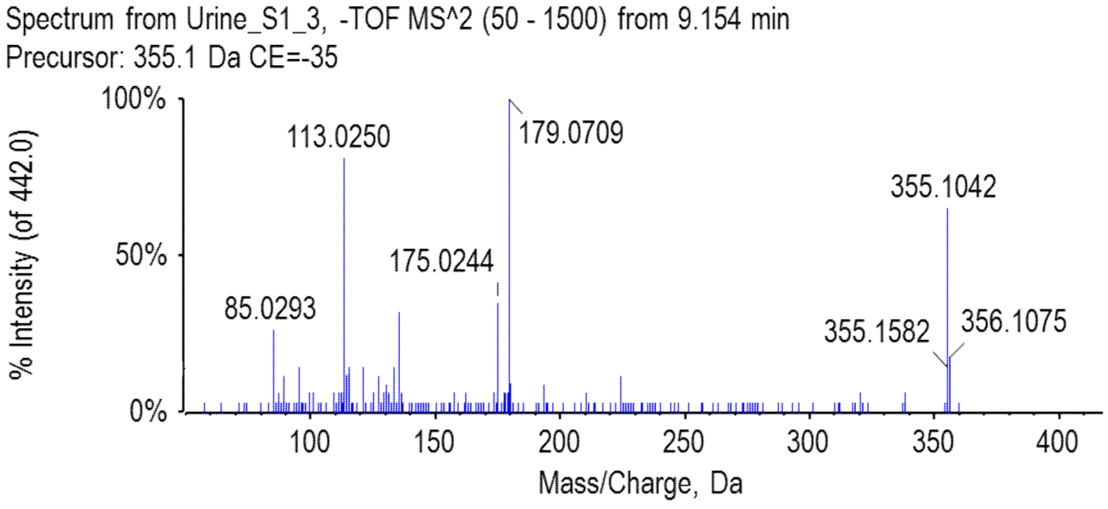

C7. Feruloylglycine (RT=9.5 min)

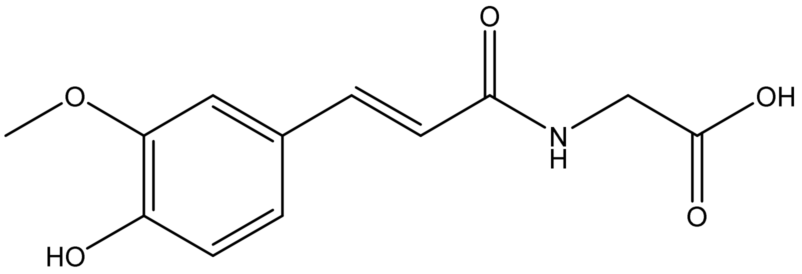

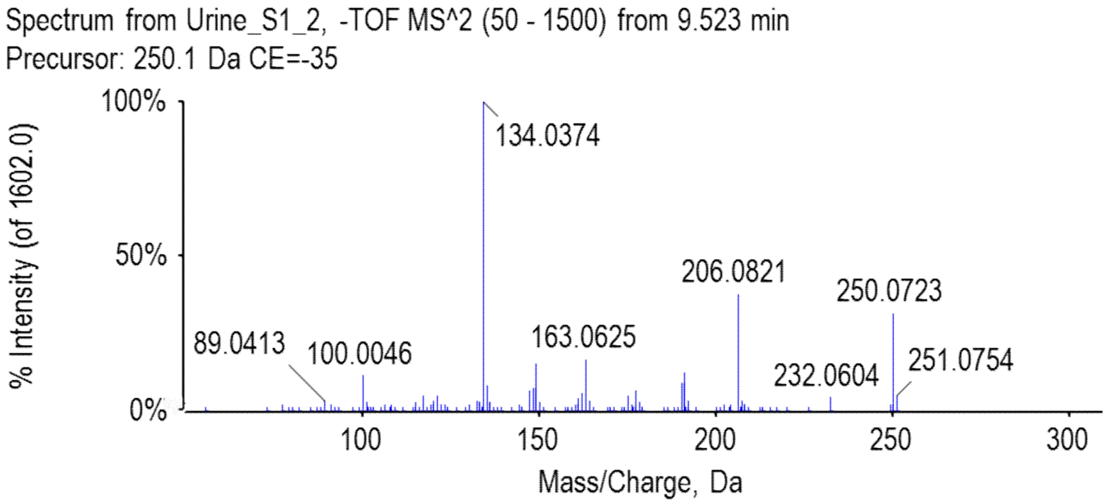

C8. 3'-Hydroxycinnamic acid or 4'-Hydroxycinnamic acid (RT=10.7 min)

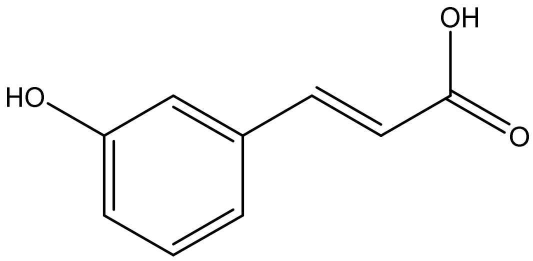

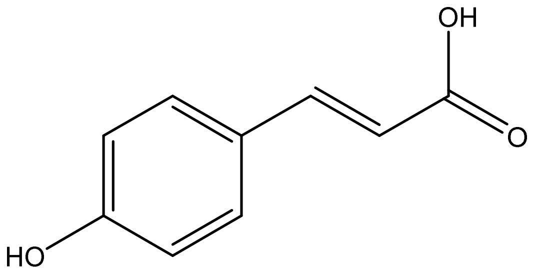

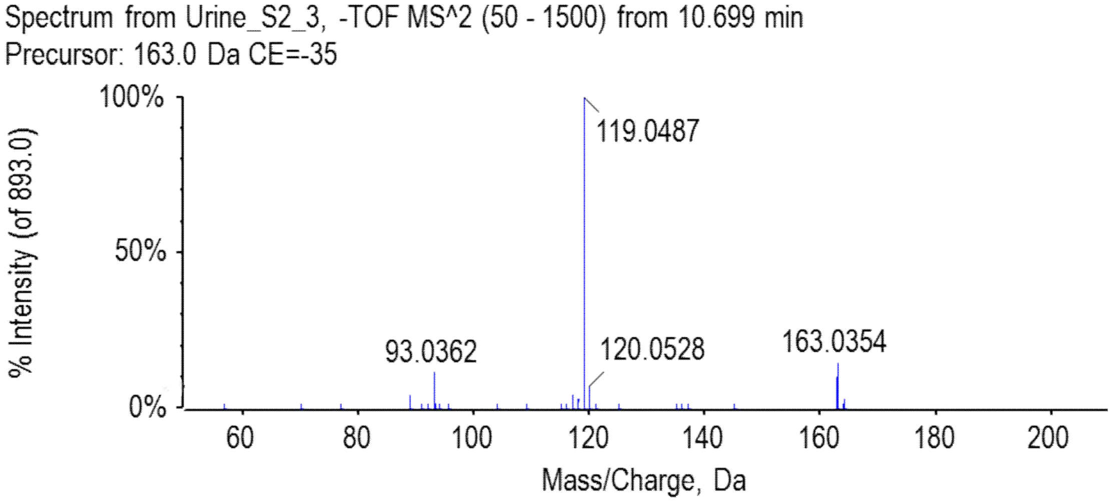

C9. 3-(3'-Hydroxy-4'-methoxyphenyl) hydracrylic acid (RT=11.6 min)

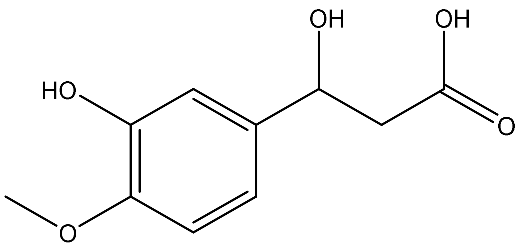

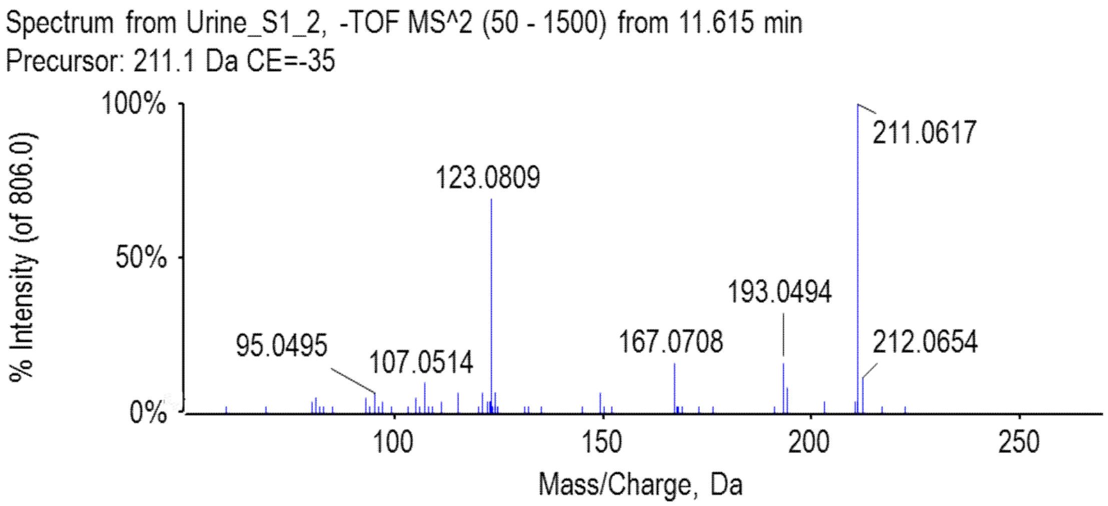

C10. 3-(3′-Methoxyphenyl) propionic acid-4′-sulfate (RT=8.6 min)

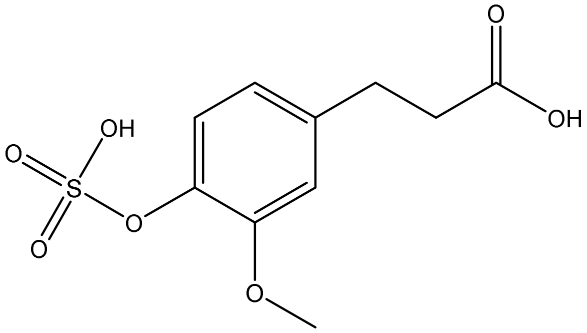

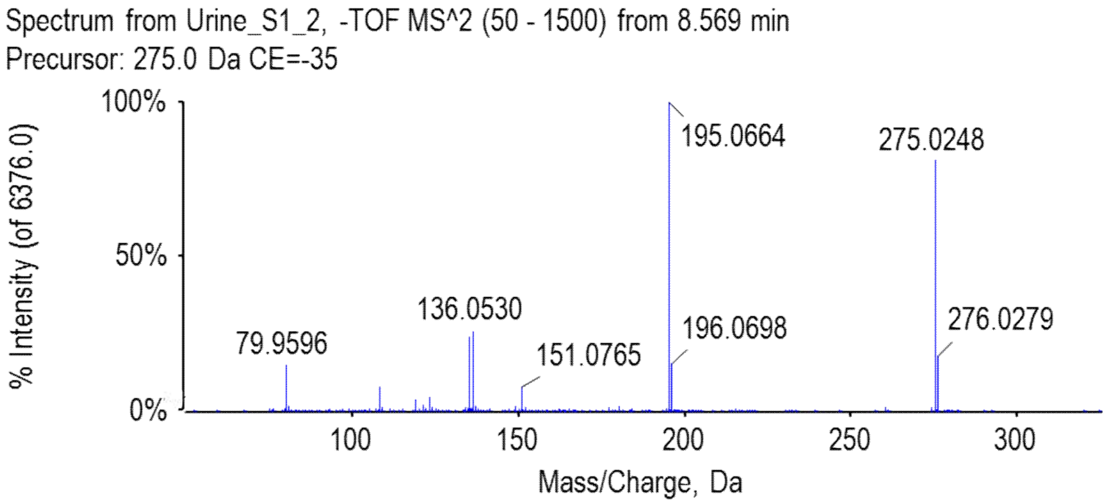

C11. 3-(4′-Methoxyphenyl) propionic acid-3′-sulfate (RT=9.0 min)

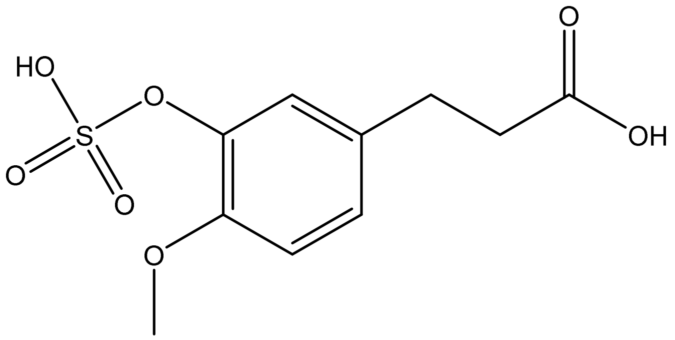

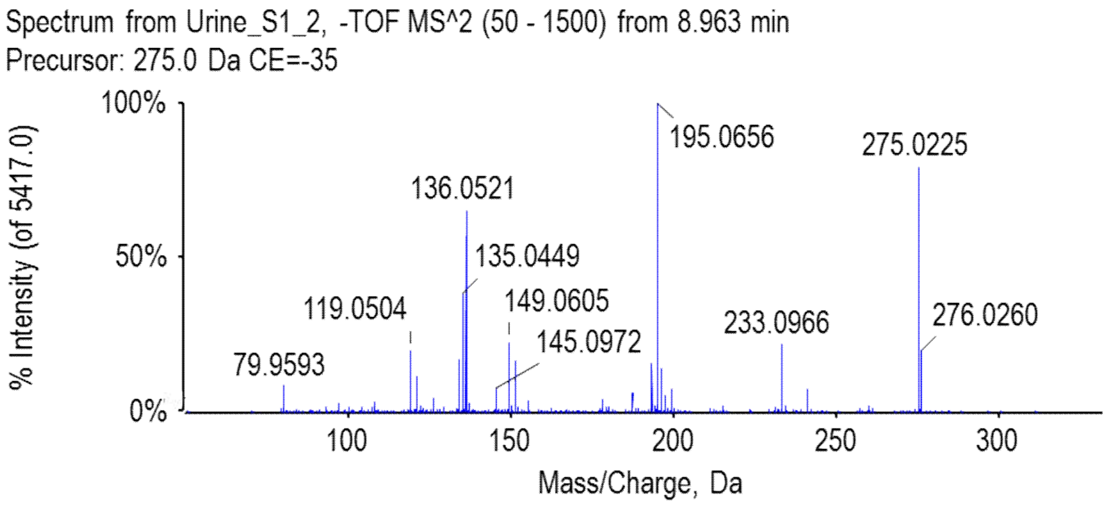

C12. 3-(3′-Methoxyphenyl) propionic acid-4′-O-glucuronide (RT=8.7 min)

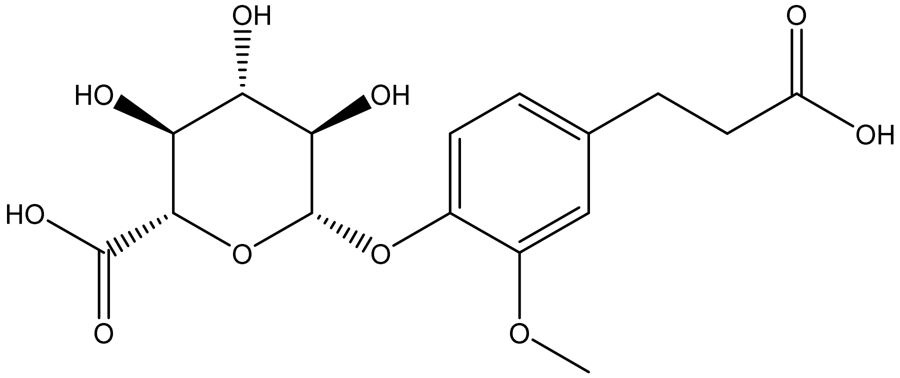

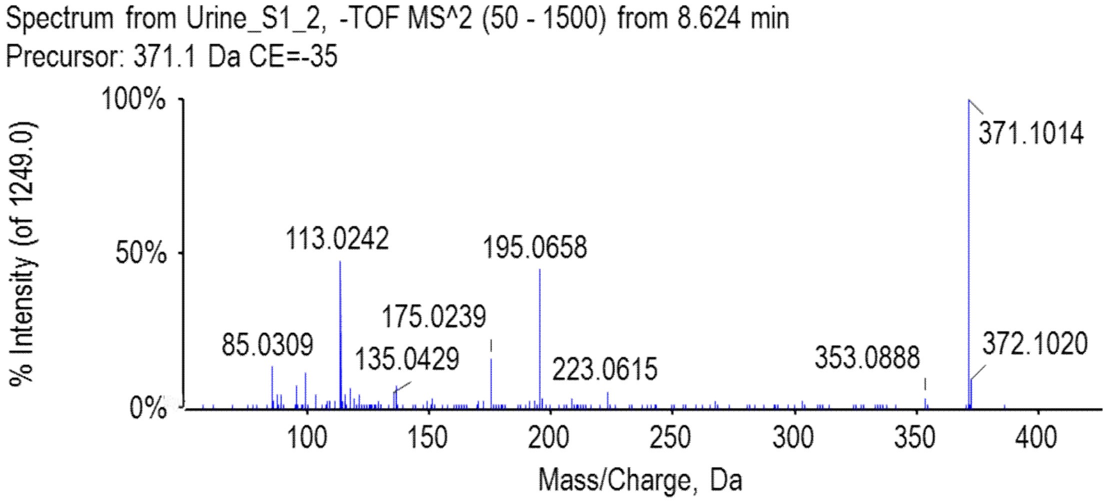

C13. 3-(4′-Methoxyphenyl) propionic acid-3′-O-glucuronide (RT=9.4 min)

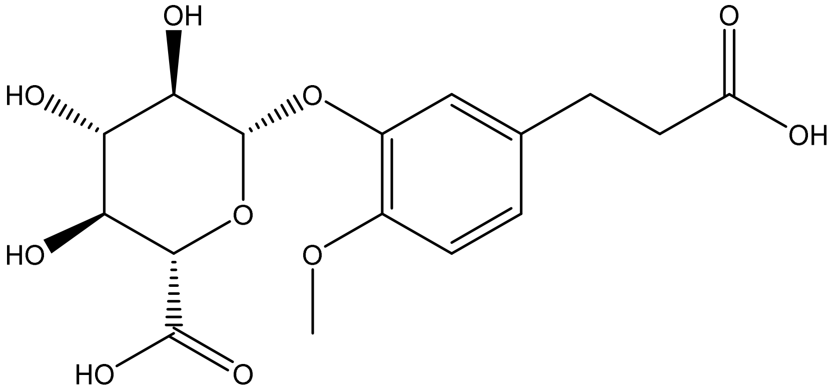

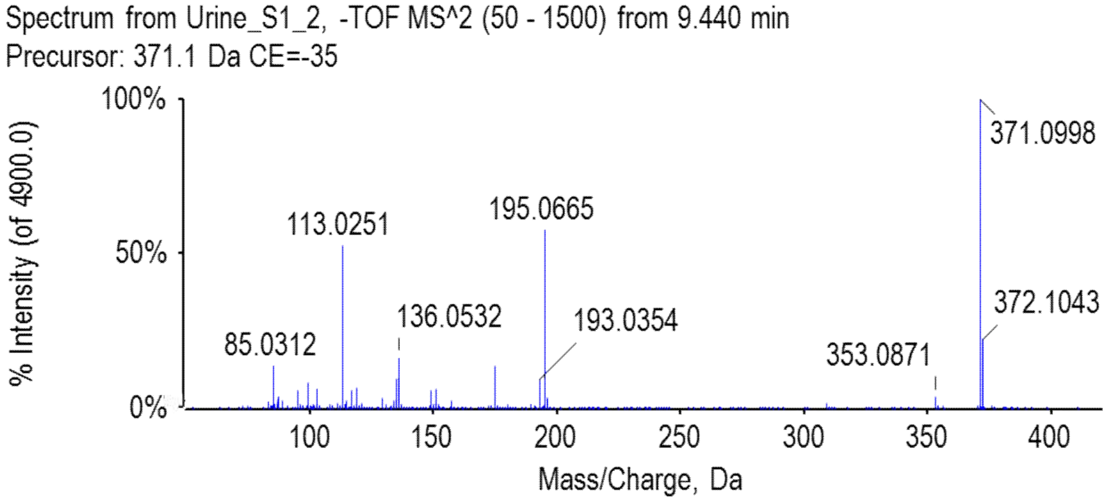

C14. 3-(4'-Hydroxyphenyl) propionic acid or 3-(3'-Hydroxyphenyl) propionic acid (RT=11.5 min)

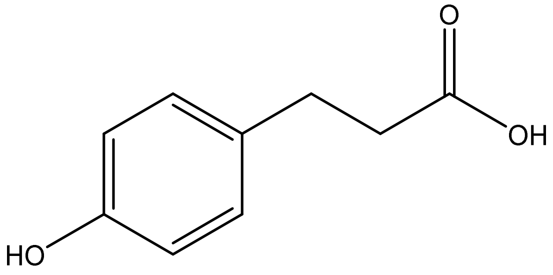

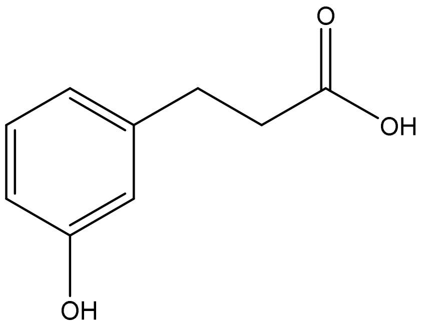

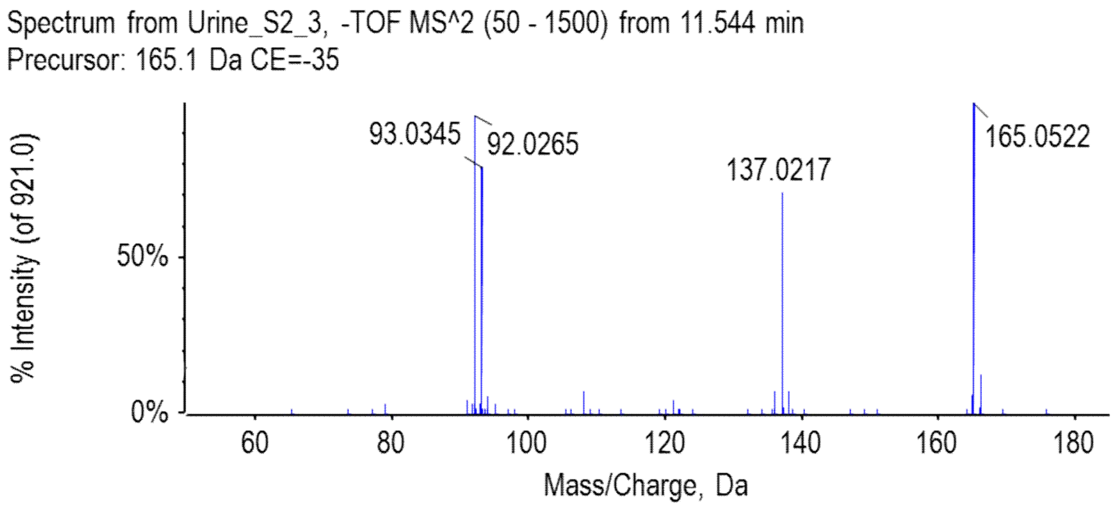

C15. 3-(3′,4′-Dihydroxyphenyl) propionic acid (RT=8.0 min)

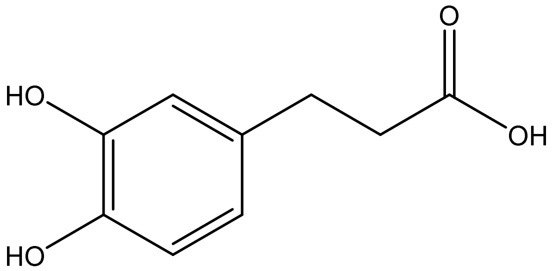

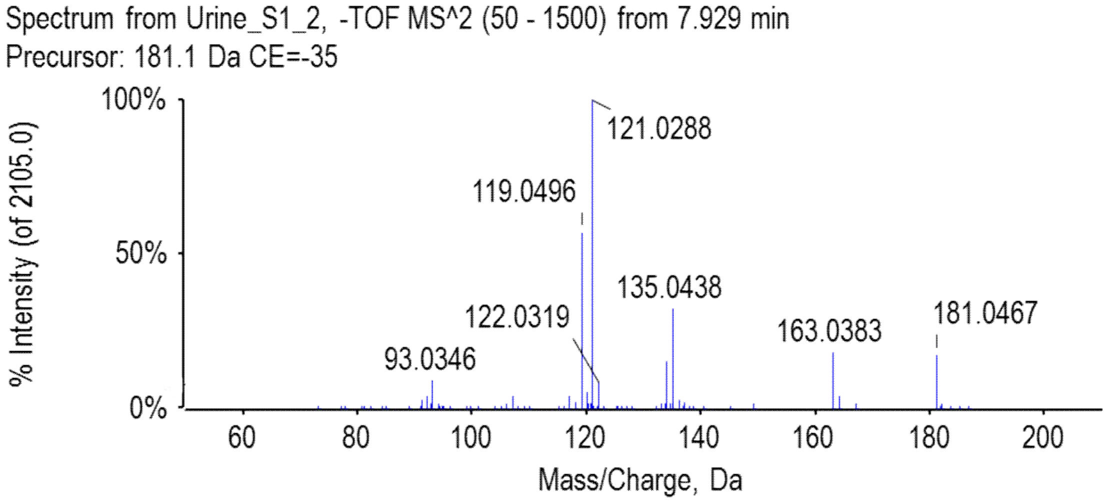

C16. 3-(3′-Hydroxyphenyl) propionic acid-4’-sulfate (RT=7.3 min)

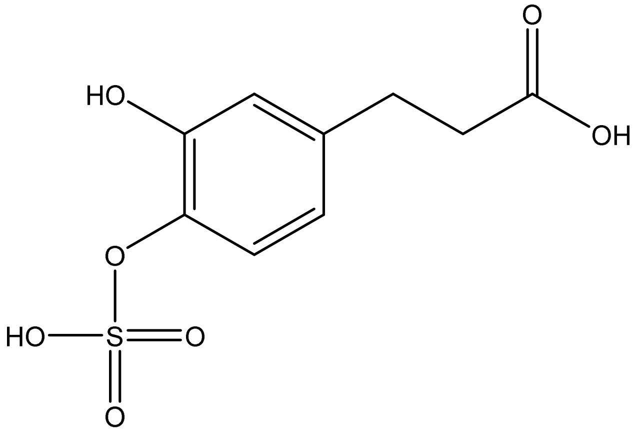

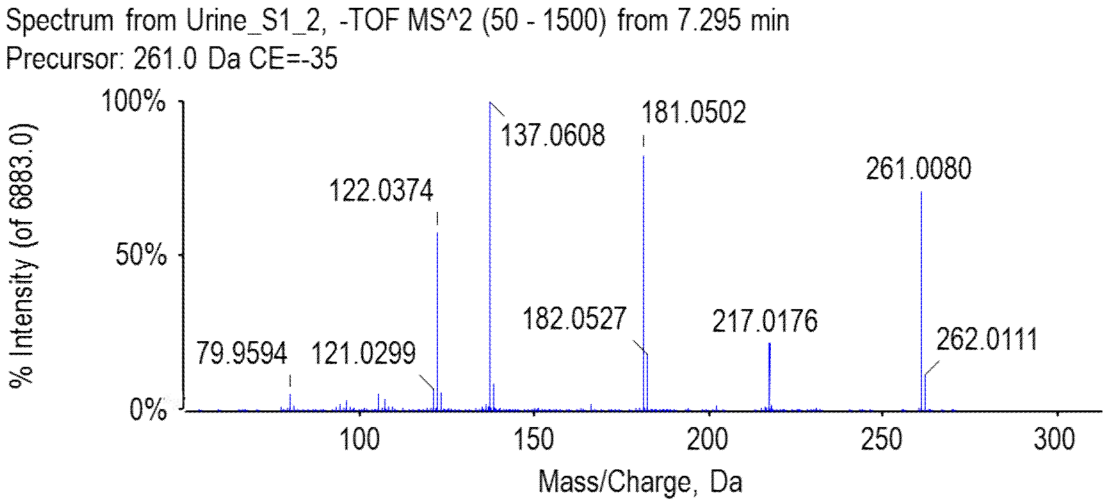

C17. 3-(4′-Hydroxyphenyl) propionic acid-3’-sulfate (RT=8.1 min)

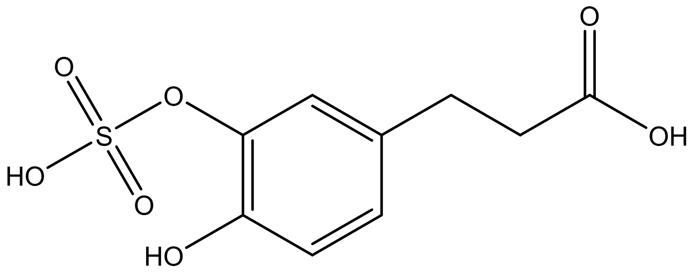

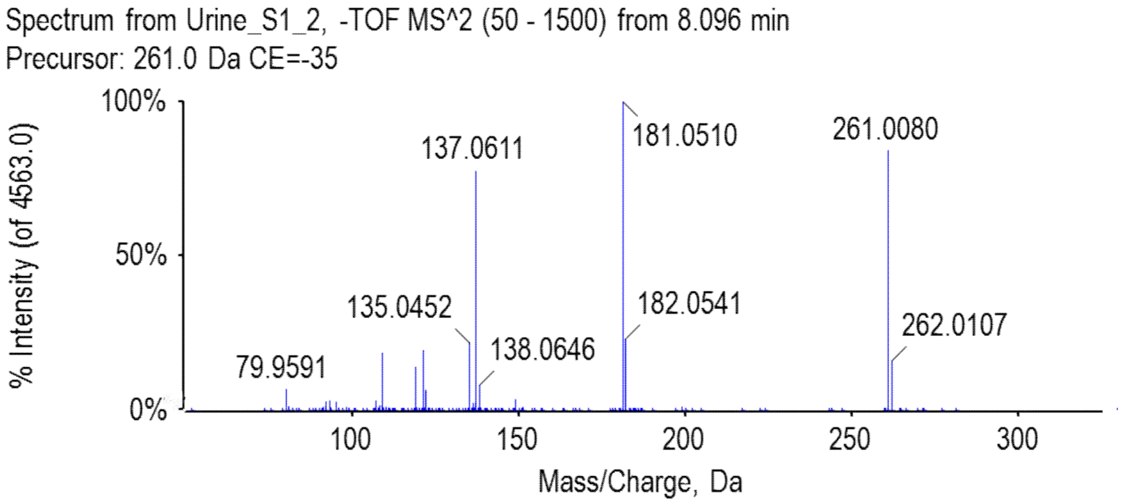

C18. 3-(Phenyl)-2-propenoic acid-4'-sulfate (RT=8.6 min)

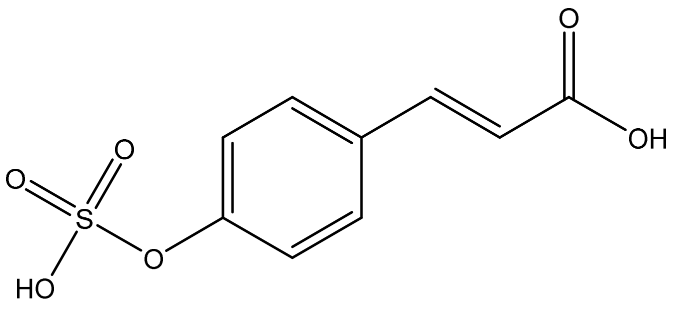

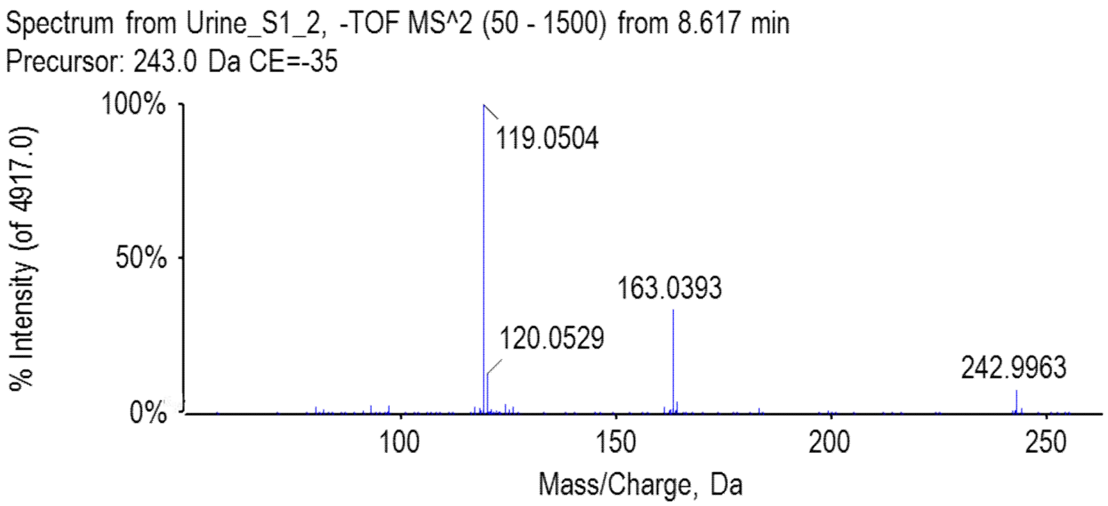

C19. 3-(Phenyl) propionic acid-4′-O-glucuronide (RT=9.0 min)

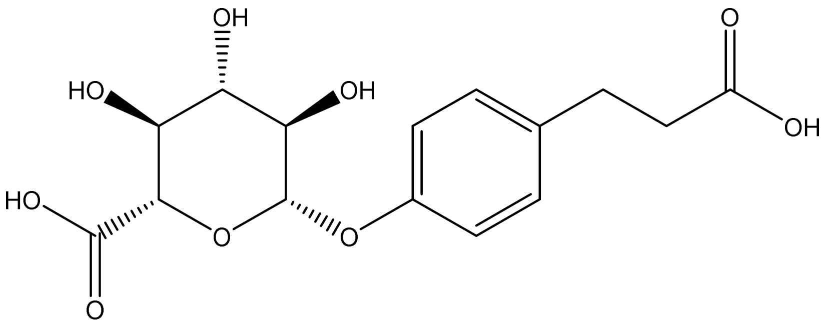

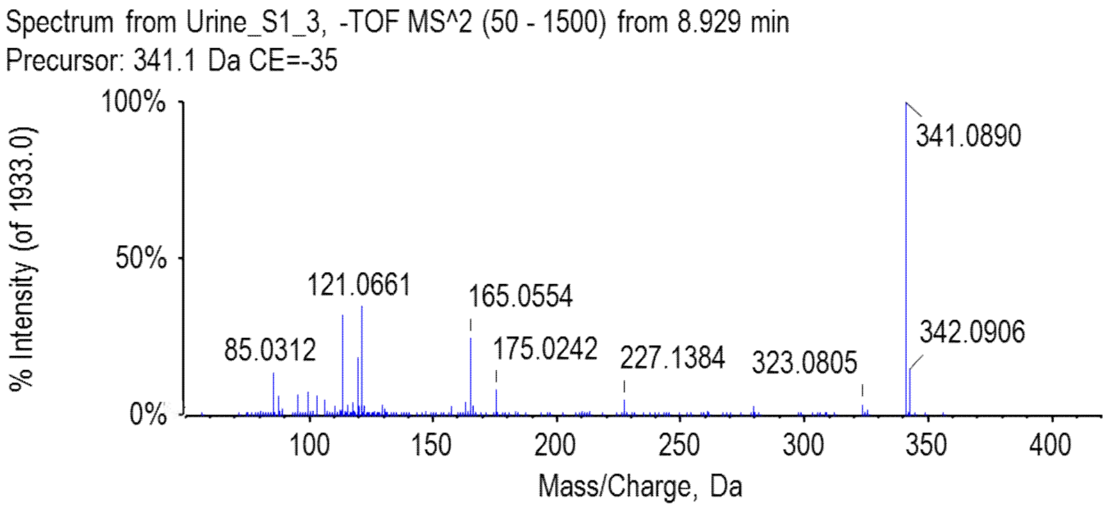

C20. 3-(Phenyl) propionic acid-3′-O-glucuronide (RT=10.9 min)

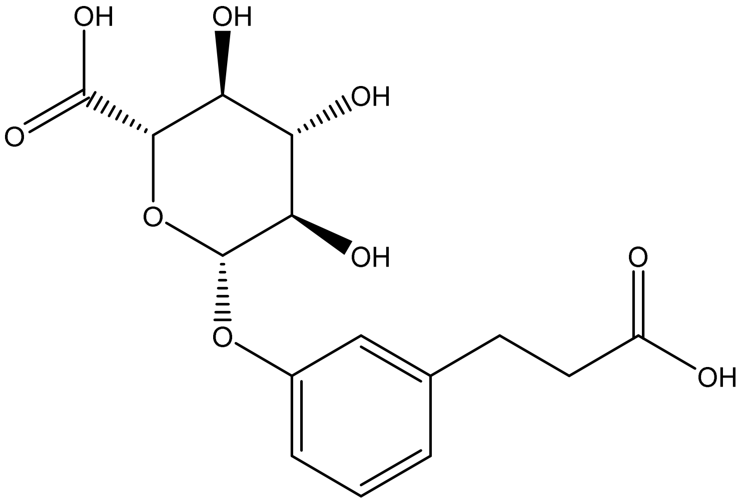

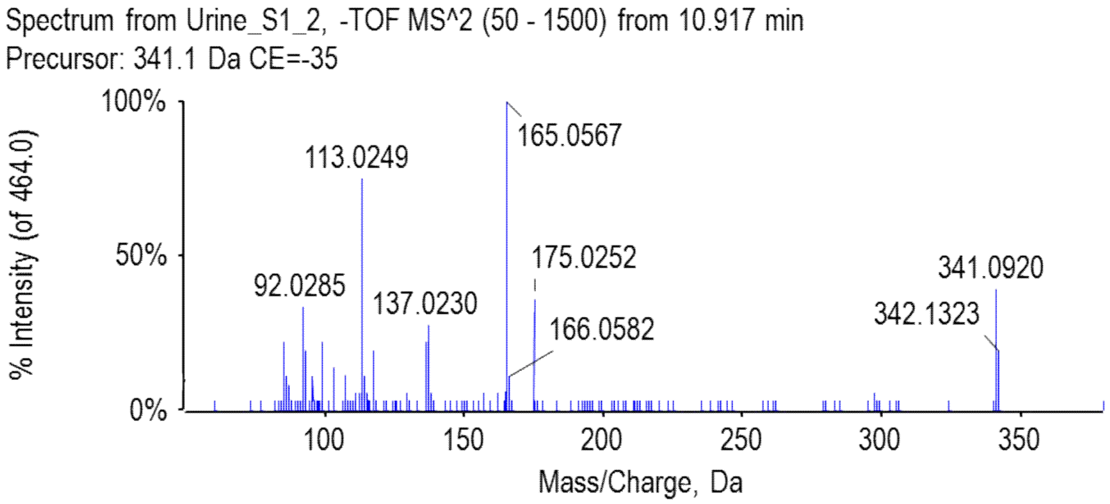

C21. Benzoic acid-4-sulfate or Benzoic acid-3-sulfate (RT=6.6 min)

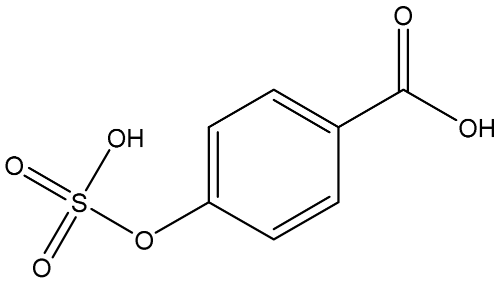

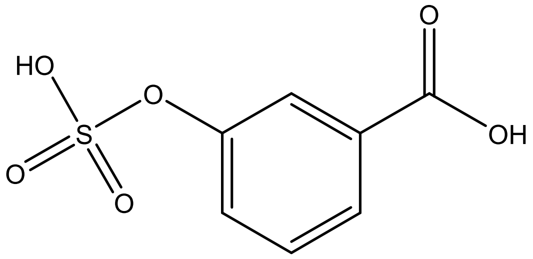

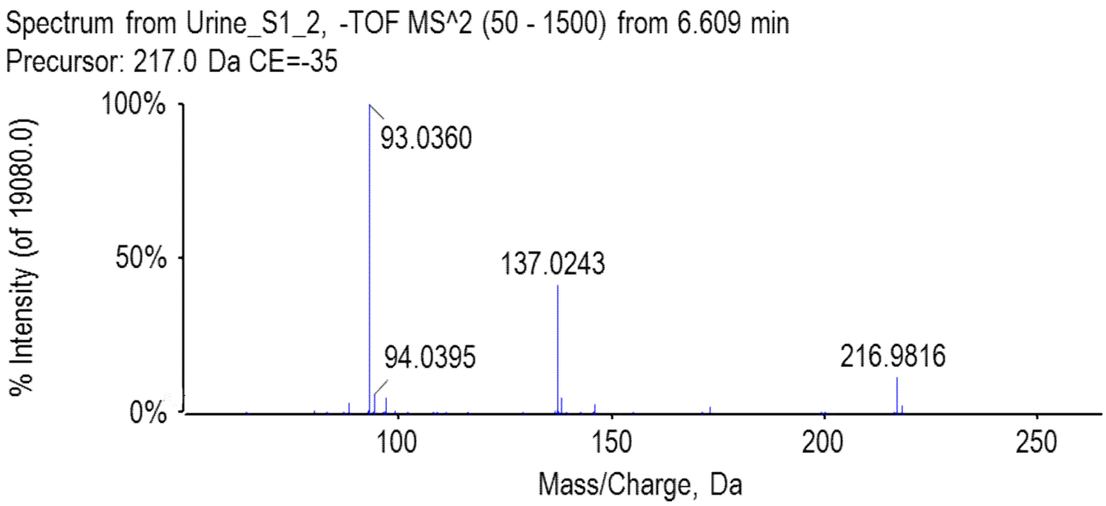

C22. 3-Hydroxybenzoic acid-4-sulfate (RT=6.7 min)

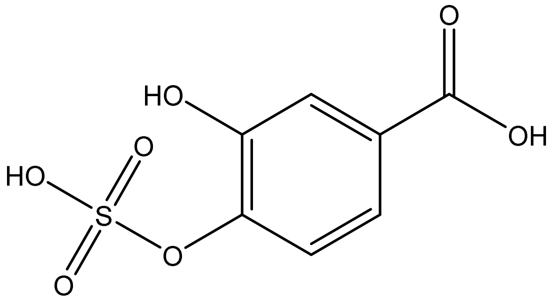

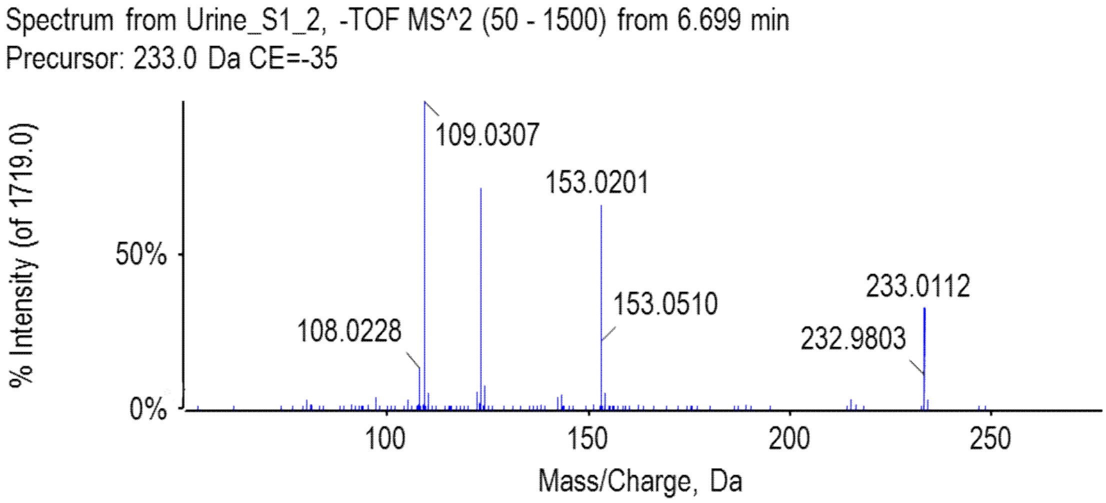

C23. 4-Hydroxybenzoic acid-3-sulfate (RT=7.2 min)

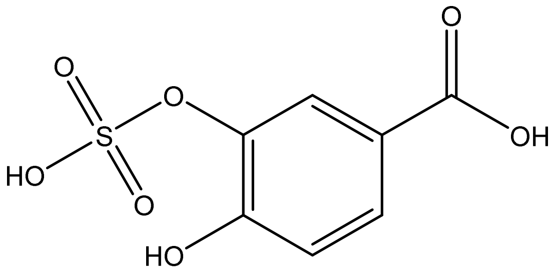

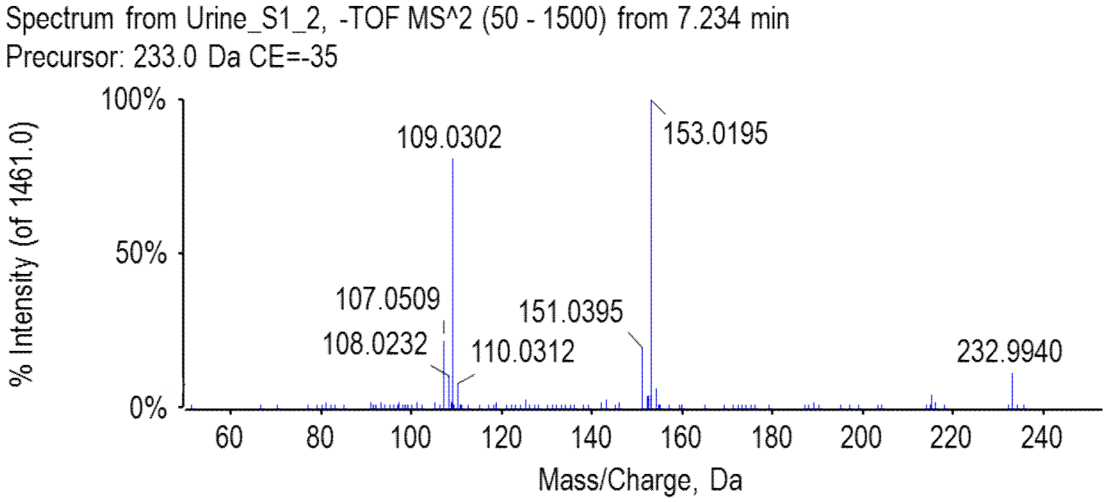

C24. Hydroxyphenylacetic acid-4′-sulfate or Hydroxyphenylacetic acid-3′-sulfate (RT=7.0 min)

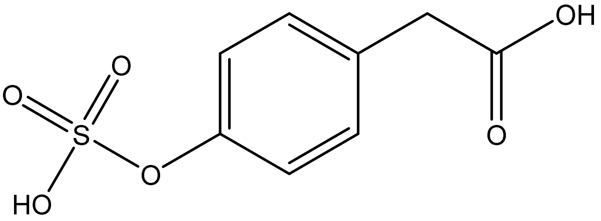

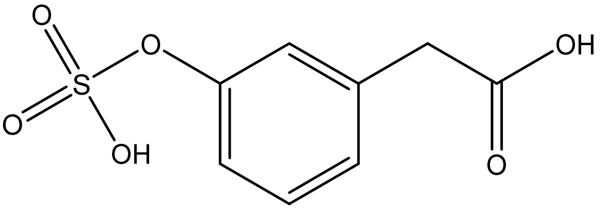

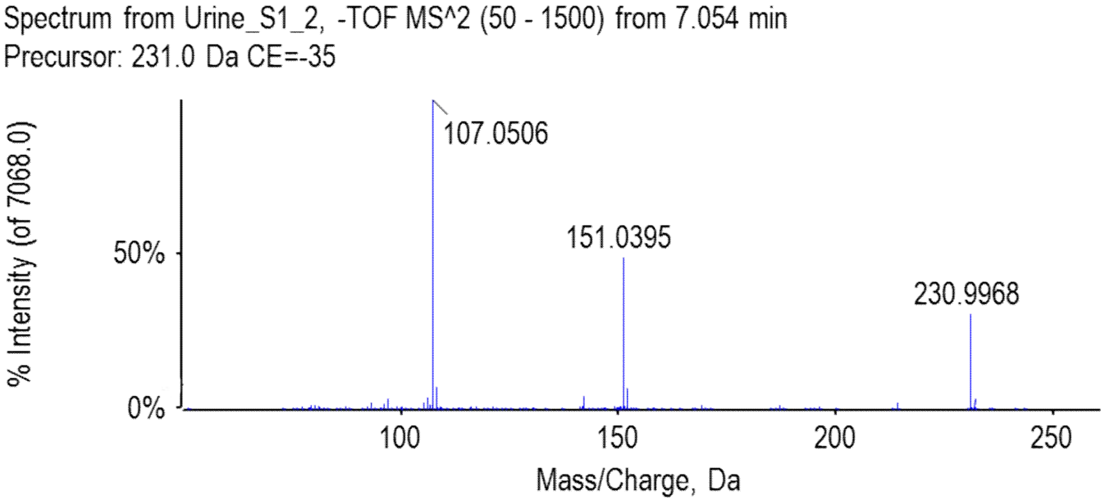

C25. 3'-Methoxy-4'-hydroxyphenylacetic acid (RT=9.5 min)

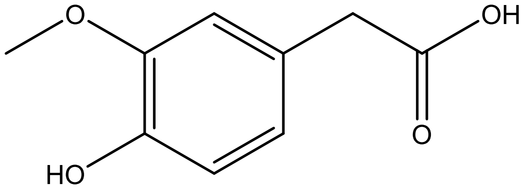

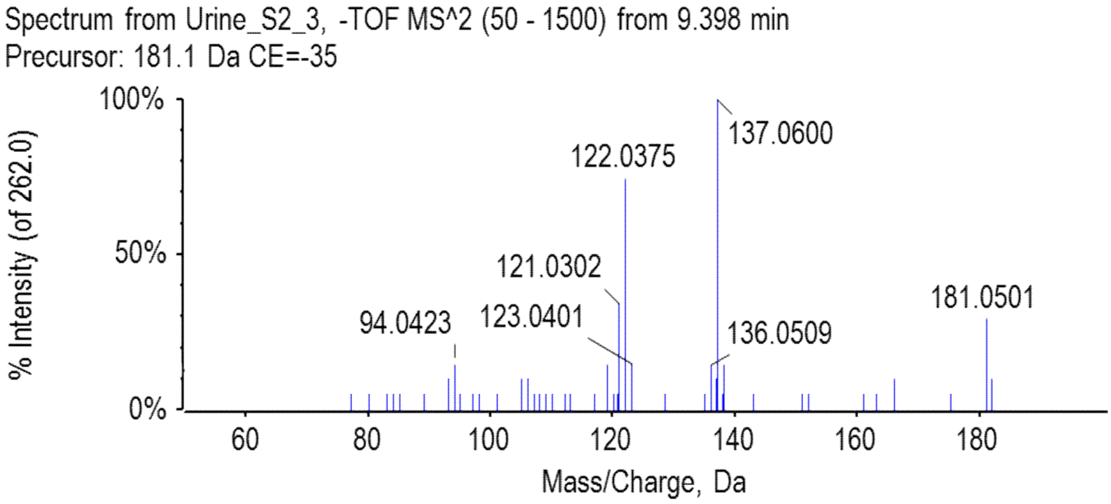

C26. 3′-Methoxy-4′-hydroxymandelic acid (RT=5.8 min)

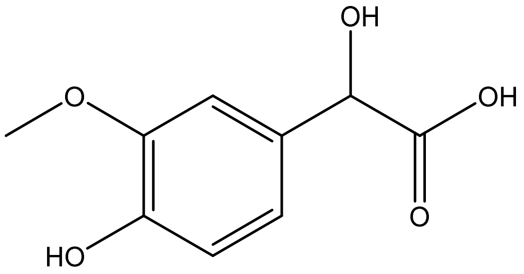

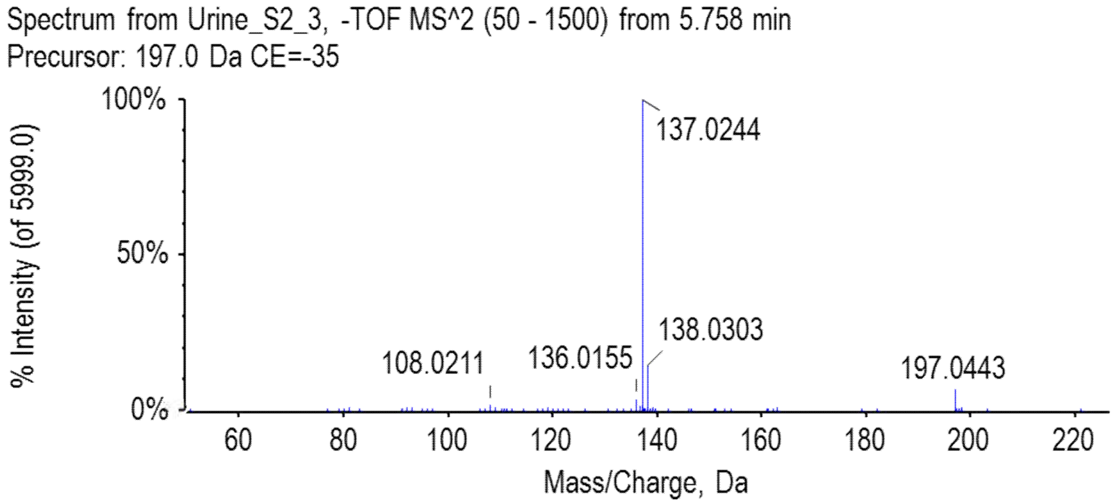

C27. Hippuric acid (RT=8.9 min)

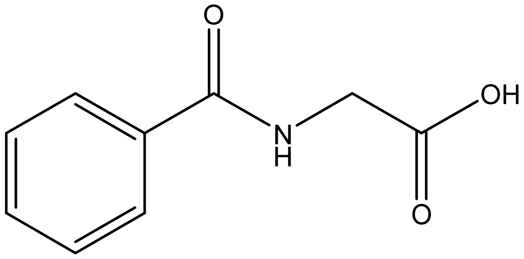

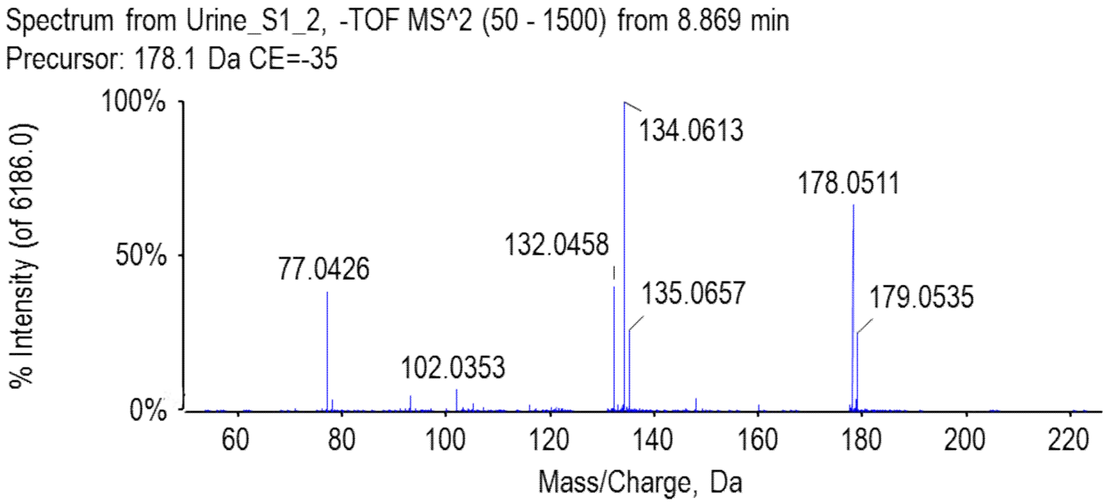

C28. 4’-Hydroxyhippuric acid (RT=7.3 min)

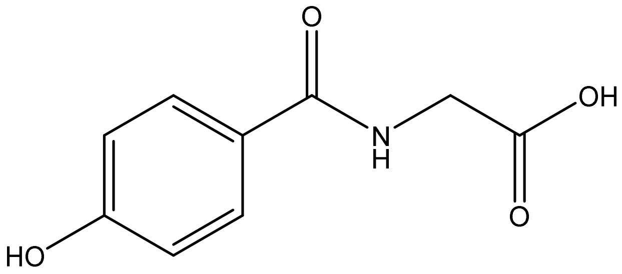

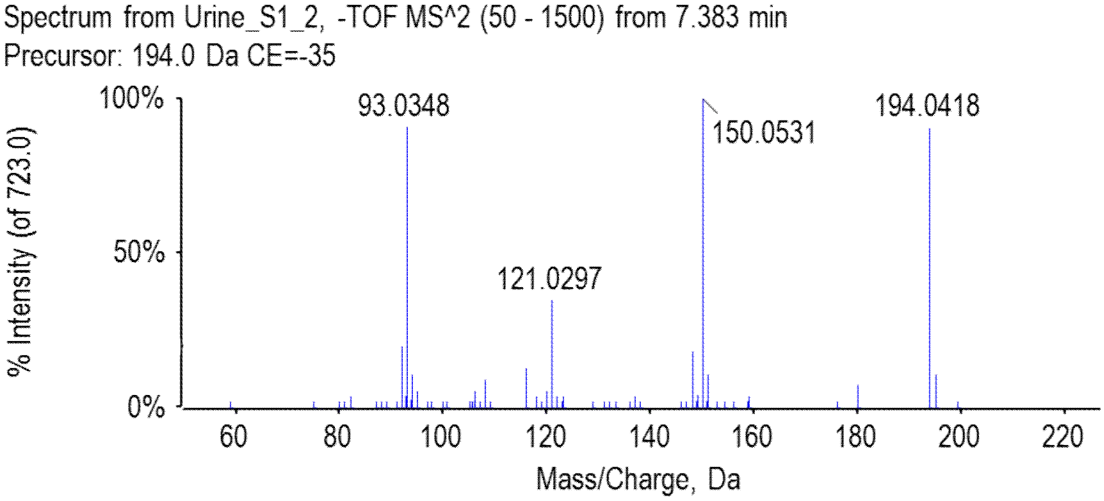

C29. 3'-Hydroxyhippuric acid (RT=10.3 min)

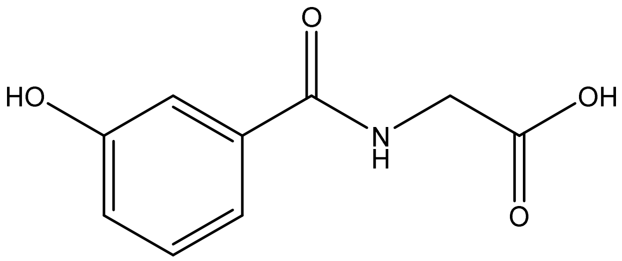

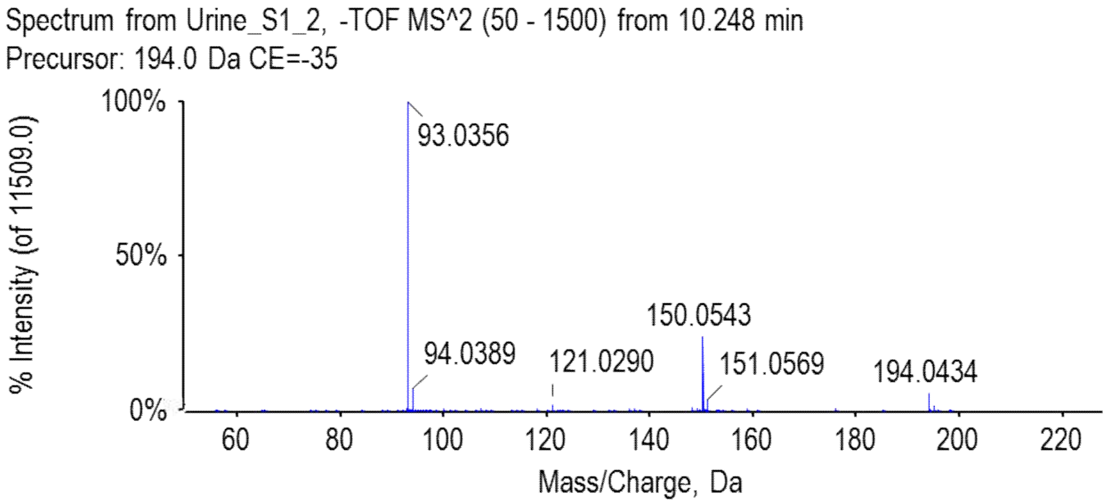

Part IV. Structures and product ion spectra of naringin-d4 (stable isotope labeled internal standard)

Naringin-d4 (RT=11.4 min)

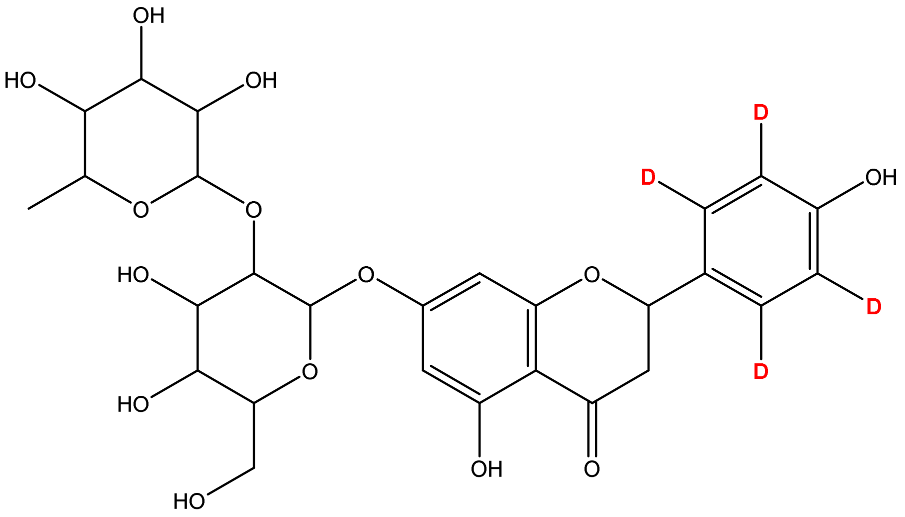

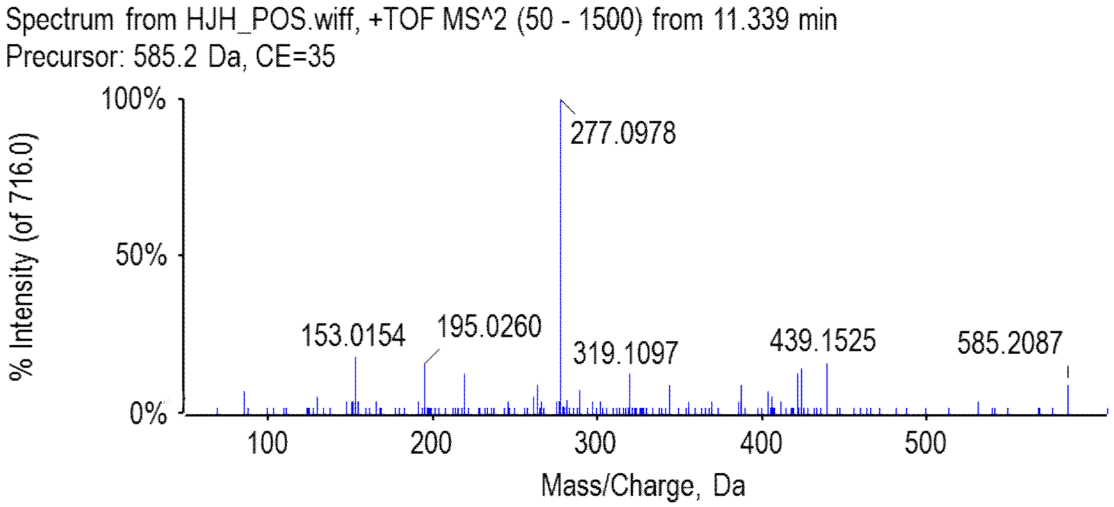

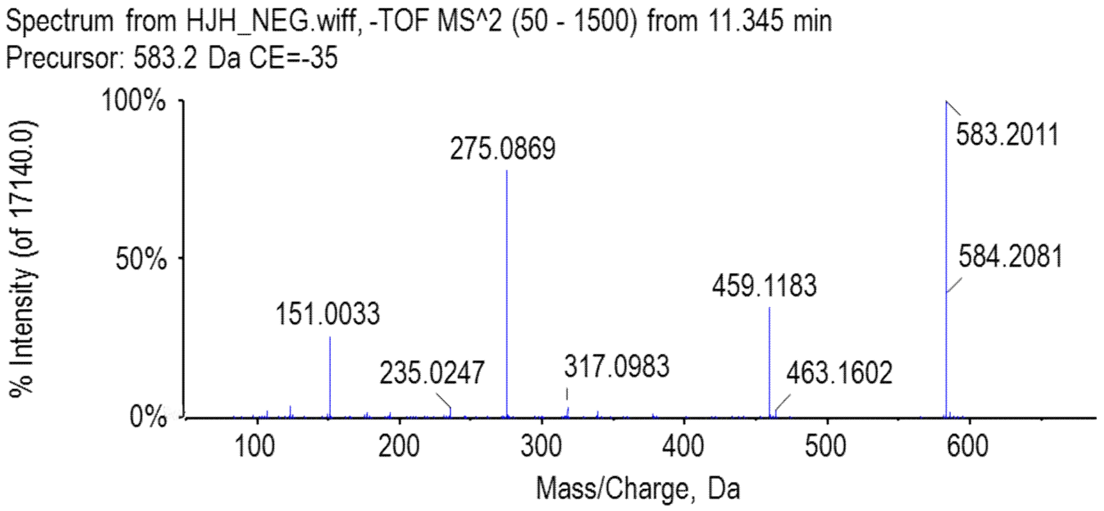

Supplement: Supplementary file 1 [file molecules-23-00895-s001.pdf]
